# Predictive Registry Optimization of Molecular Adsorbates on Solid Surfaces

**DOI:** 10.1002/anie.1693717

**Published:** 2026-06-13

**Authors:** David A. Hofmeister, Christian E. Selzer, Laura zur Horst, Simon C. Rickert, Julia Kohn, Andreas Hansen, Stefan‐S. Jester, Sigurd Höger

**Affiliations:** ^1^ Kekulé‐Institut Für Organische Chemie Und Biochemie Rheinische Friedrich‐Wilhelms‐Universität Bonn Bonn Germany; ^2^ Mulliken Center for Theoretical Chemistry Rheinische Friedrich‐Wilhelms‐Universität Bonn Bonn Germany

**Keywords:** chirality, molecular spoked wheels, registry analysis, scanning tunneling microscopy

## Abstract

Understanding and predicting how large organic molecules adsorb on crystalline substrates is essential for designing functional surfaces in electronics, catalysis, and supramolecular chemistry. Standard quantum‐chemical approaches often fail for large π‐conjugated molecules due to size and conformational diversity. We present a physically intuitive, generalizable registry analysis that overcomes this by maximizing the overlap between surface‐oriented hydrogen atoms (“spikes”) and the periodic graphite hexagon centers (“pockets”) in a Monte Carlo‐like approach. Inspired by the alkane‐on‐graphite model, the framework extends to structurally complex architectures to assign adsorption geometries with high resolution, without costly computations. Registry scoring applied to STM data enabled determining the absolute conformation of atropisomers, demonstrating geometric registry as an applicable measure for predicting large‐molecule arrangements on surfaces.

## Graphical abstract

Local C–H “spikes” from a π‐conjugated backbone lock into graphene “pockets”. A geometric registry score turns images obtained by scanning tunneling microscopy at the solid/liquid interface into atomic‐scale adsorption models, enabling assignment of surface atropisomers in large π‐conjugated molecules.

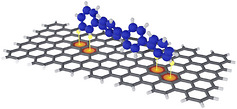

## 1 Introduction

π‐conjugated materials on solid surfaces are key building blocks for next‐generation electronics, optoelectronics, and energy conversion technologies, with applications ranging from organic field‐effect transistors and solar cells to sensors and catalytic interfaces [1, 2, 3]. In particular, two‐dimensional (2D) molecular assemblies offer a promising route to engineer functional surfaces with tailored nanopatterns, specific electronic properties, or chiral recognition sites [4, 5]. Achieving such functionality critically depends on understanding and controlling how organic molecules adsorb and self‐assemble on crystalline substrates. Graphite (HOPG) serves as a prototypical, atomically flat crystalline substrate and is the layered bulk form of graphene, making it a key platform for studying molecular adsorption phenomena [6]. Scanning tunneling microscopy (STM) is highly suited to experimentally probe these and often provides submolecular resolution, also at the solid/liquid interface under ambient conditions.

Self‐assembled monolayers (SAMs) of *n*‐alkanes on graphite exhibit well‐understood registry driven by a near‐perfect match of hydrogen atom footprints with the substrate lattice as described by Groszek [7, 8, 9, 10, 11]. In the energetically most favorable packing, the alkyl chains align parallel to both the substrate and to one another, with their surface‐facing hydrogen atoms registering in the centers of the hexagons of the uppermost graphene layer. Alkyl substituents are also applied to mediate and manipulate the physisorption of more complex sp‐ and sp^2^‐based carbon compounds, which can be viewed as bodies of certain sizes, shapes, and functionalities [5, 6, 12, 13].

**FIGURE 1:**
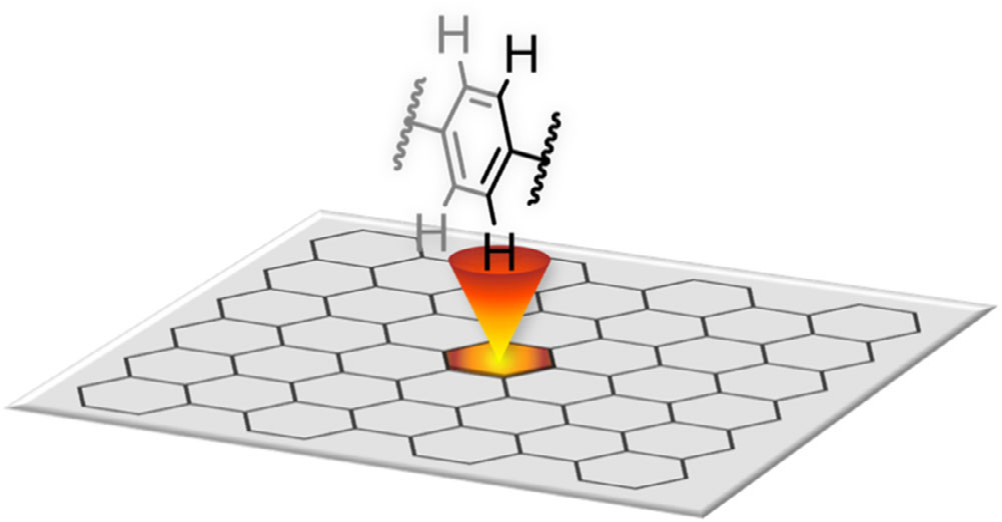
Schematic representation of a “spike” (surface‐oriented hydrogen atom) orienting toward a “pocket” (center of a graphene hexagon). Color distribution from light to dark colors refers to an increasing horizontal distance from the center of a graphene hexagon

However, the accurate theoretical modelling of the adsorption of large π‐conjugated systems remains a fundamental challenge. High‐level quantum chemical calculations, such as local explicitly correlated coupled cluster with large basis sets, are computationally prohibitive [14], whereas cost‐effective semi‐empirical (SQM) methods like GFN2‐xTB [15] remain too inaccurate, especially in the context of ranking conformers [16, 17, 18]. Here, we address this challenge by introducing a physically intuitive and computationally efficient registry analysis utilizing the overlap between surface‐oriented hydrogen atoms (“spikes”) and periodic centers of the substrate's hexagonal lattice (“pockets”, Figure 1). The analysis generalizes the classic alkane‐on‐graphite Groszek model to large π‐conjugated systems, and we apply it to **1**, a molecular spoked wheel (MSW) investigated by STM.

## 2 Results and Discussion

**SCHEME 1:**
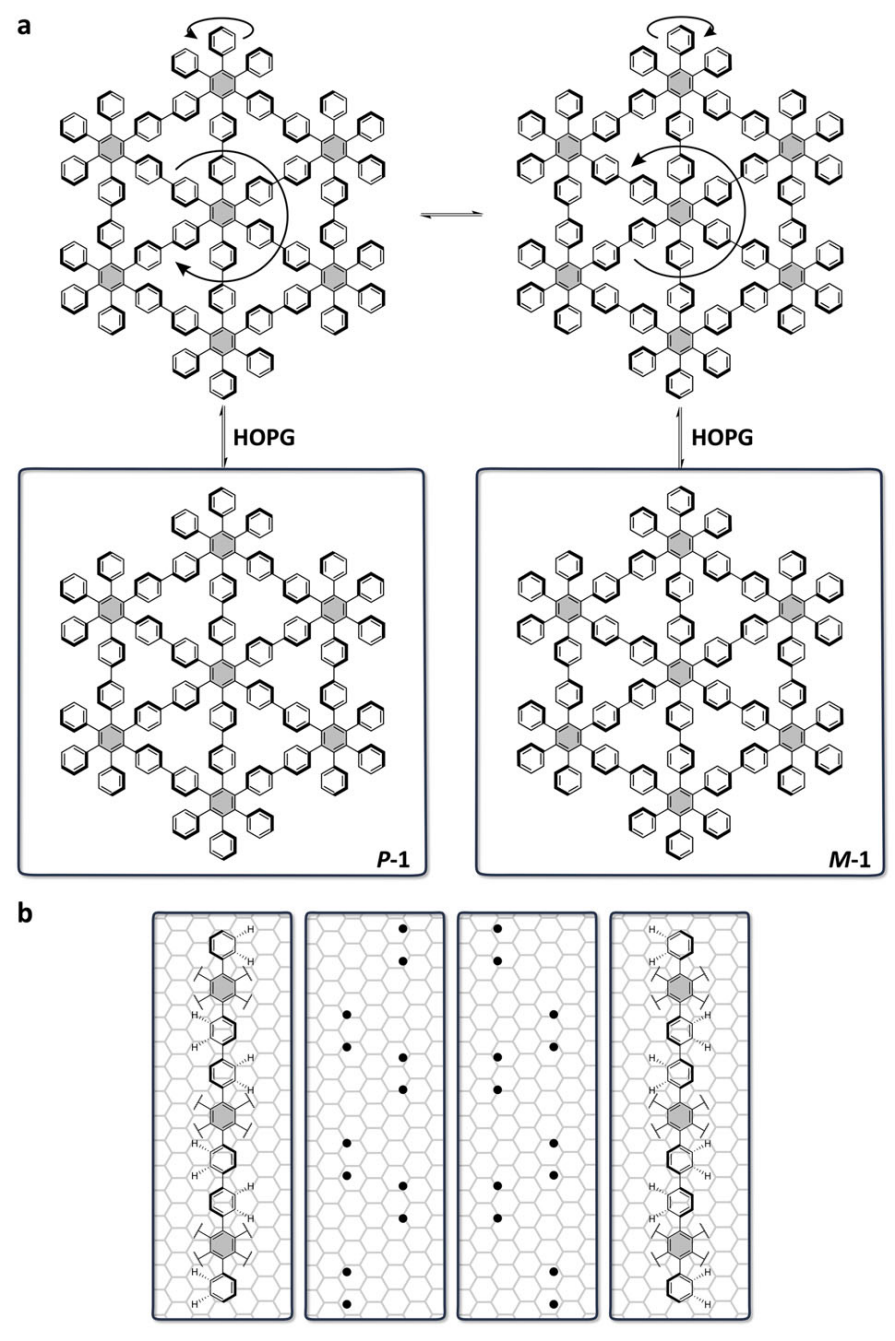
(a) Interconversion of the two enantiomers of **1** by rotation of the phenyl(ene) units about single bonds, and their adsorption onto HOPG; (b) nonaphenylene cutouts illustrating the enantiomorphic hydrogen‐atom contact patterns (“footprints”) of both atropisomers on the substrate. For clarity, other phenyl(ene) substituents are omitted; aromatic units oriented parallel to the molecular plane are shown in grey

We designed and synthesized the π‐conjugated *C*_6_‐symmetric all‐phenylene MSW **1** [19, 20, 21, 22], which is a cutout of graphenylene‐1 (Scheme 1) [23, 24, 25]. It matches the symmetry of the substrate and consists of seven covalently connected hexaphenylbenzene units in which each central aromatic ring is fixed in an orientation parallel to the molecular plane (Scheme 1, grey). The remaining 42 of the 49 aromatics adopt a more or less tilted orientation, with the six phenyl(ene) units around each center arranged in a propeller‐like fashion. The biphenylene bridges that connect these centers are twisted and this mechanically couples the orientations of all phenyl(ene) units across the entire molecule, significantly reducing the overall conformational freedom. As a result, changing the orientation of a single phenyl(ene) unit relative to the MSW plane induces a coupled flip of all other phenyl(ene) units. This leads therefore to two enantiomorphic conformations which rapidly interconvert in solution (Scheme 1a, top), as also supported by a Molecular Dynamics (MD) simulation on the GFN‐FF level of theory [26, 27, 28] (see an MD simulation in mov 1 in the Supporting Information). Viewing from one corner of the molecule to the opposite corner shows that the nine linearly connected phenyl(ene)s can adopt a right‐handed (*P*, Scheme 1a, left side) or left‐handed (*M*, Scheme 1a, right side) helix, interchangeable by a phenyl flip, as described above. However, when **1** is adsorbed on HOPG, the conformations are frozen in (Scheme 1a, bottom, further see an MD simulation in mov 2 in the Supporting Information). As a consequence, also the hydrogen‐atom footprints of the molecule depend on its specific conformations and are enantiomorphic to each other (Scheme 1b, nonaphenylene cutouts). Although homochiral packing is often favored on surfaces, it remains difficult to predict whether adsorbed enantiomers will segregate into homochiral domains, form racemic mixtures, or even be distinguishable at all by surface‐sensitive techniques [29].

**FIGURE 2:**
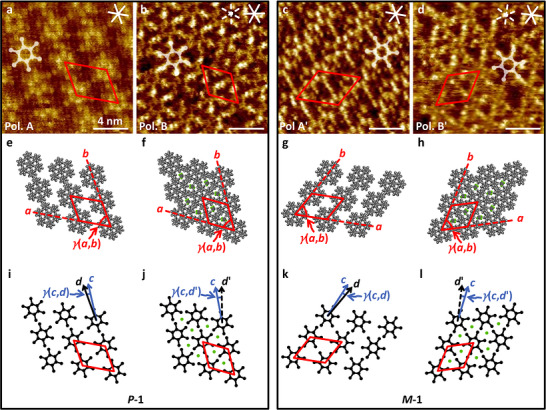
(a–d) STM images, (e–h) (supra‐) molecular models, and (i–l) schematic models of supramolecular nanopatterns of **1** at the solution/HOPG interface using TCB as solvent. (a, e, i) Polymorph A of *P*‐**1** (clockwise backbone rotation relative to the HOPG zigzag direction); (b, f, j) polymorph B of *P*‐**1** (counterclockwise backbone rotation relative to the HOPG armchair direction); (c, g, k) polymorph A’ of *M*‐**1** (counterclockwise backbone rotation relative to the HOPG zigzag direction); (d, h, l) polymorph B’ of *M*‐**1** (clockwise backbone rotation relative to the HOPG armchair direction). All STM images show an area of 15.0 × 15.0 nm^2^. The red, blue, as well as solid and dashed white (black) lines or arrows indicate unit cells (*a*, *b*), backbone orientations (*c*), as well as HOPG zigzag (*d*) and armchair (*d*’) directions, respectively. All samples were thermally annealed to 80°C for 20 s prior to STM imaging as a routine procedure in order to promote 2D self‐assembly. White schematic models in the STM images represent one molecule

At the solution/solid interface of **1** in 1,2,4‐trichlorobenzene (TCB) and highly oriented pyrolytic graphite (HOPG), SAMs are formed and were investigated by STM (Figure 2 and Supporting Information) at room temperature with the STM tip immersed into the solution [30]. Three distinguishable 2D crystalline polymorphs (A, B, and C) were observed, and a detailed STM discussion using overview STM images is given in Chapter 5 in the Supporting Information. Each of the repeating units (white overlaid schematic models) contains a dark center and a hexagonal‐ish rim, surrounded by six bright dot‐shaped image regions in the periphery. These are attributed to the central electronically decoupled phenylene hub, the six biphenylene units of the spokes, and the 18 peripheric phenyl substituents, respectively. The actual contrast correlates with the more or less effective local electronic coupling of the different building blocks with the underlying HOPG substrate. In polymorph A (Figure 2a,e,i), the three nonaphenylene subunits of **1** are oriented along *c* and tilted clockwise relative to the HOPG zigzag directions *d*, indicated by *γ*(*c*,*d*) = –(7 ± 2)° [21]. In contrast, in polymorph B (Figure 2b,f,j), the MSWs are oriented along *γ*(*c*,*d*’) = +(11 ± 2)° relative to the armchair direction *d*’. In polymorph C (see Supporting Information), molecules are oriented according to polymorph A but pack more densely. All polymorphs were imaged as homochiral domains, and we observed their respective enantiomeric packings (cf. Figure 2 right panel for pol. A’ and B’, and see Supporting Information for pol. C’). It is not clear whether *γ*(*c*,*d*) and *γ*(*c*,*d*’) correlate with the conformation of **1**. Moreover, the absolute conformation cannot be resolved directly by STM here.

**FIGURE 3:**
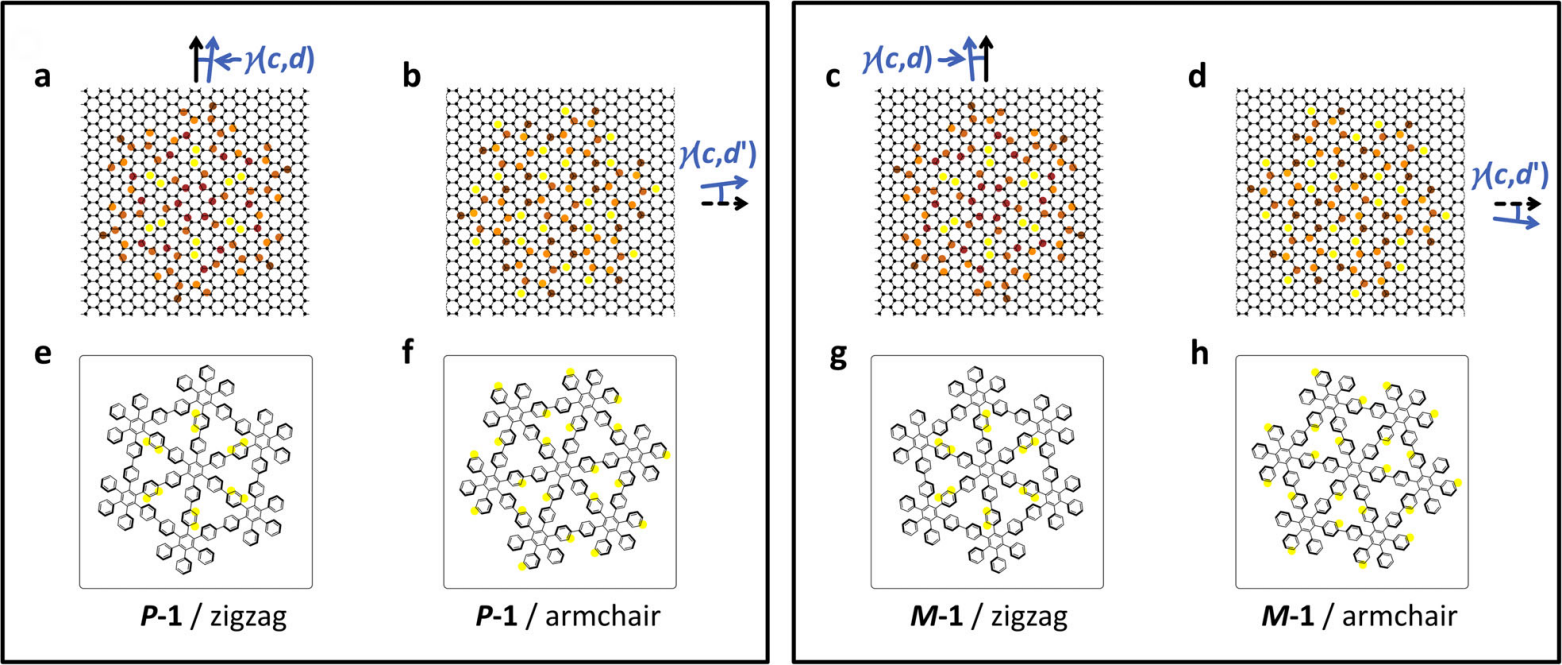
(a–d) 2D plots of optimized molecular registries of (a, b) *P*‐**1** and (c, d) *M*‐**1** on a graphene cutout; the color gradient (light to dark) indicates increasing distances between the hydrogen atoms and the centers of the hexagons with *a*_0_ = 2.46 Å, that is, the lattice vector of graphene; (e–h) chemical structures of the absolute conformations of *P*‐**1** and *M*‐**1**, as indexed to the domains, with registry positions highlighted in yellow (hydrogen‐to‐center distance ≤ 0.2 Å)

**FIGURE 4:**
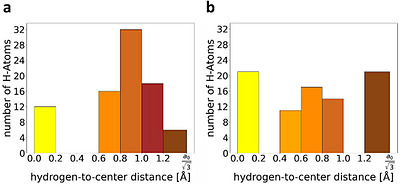
(a, b) Histograms showing the distribution of the distances between the hydrogen atoms of *P*‐**1** (and *M*‐1) and the centers of the hexagons in the graphene cutout, for the orientations relative to the (a) zigzag and (b) armchair axes

We aimed to gain a deeper understanding of the molecular registry with respect to the substrate, in order to rationalize the observed rotation of the molecular backbone directions *c* relative to the substrate's zigzag or armchair directions, *d* and *d*’. Consequently, we performed quantum chemical calculations of **1** adsorbed on a graphene sheet cutout at the GFN1‐xTB [31] level of theory (GFN1‐xTB was preferred over GFN2‐xTB [15] due to a better SCF convergence behavior). However, the relative energies of different adsorption positions and orientations of **1** on graphene differed by less than 5 kJ/mol, indicating no clear energetic preference within the method's accuracy. More accurate methods, such as local explicitly correlated coupled cluster with large basis sets are not feasible for systems of this size [14]. Therefore, we investigated a completely different approach to tackle this problem. The registry of *n*‐alkanes on HOPG is well understood and described by the Groszek model, in which the alkyl chains align along one of the HOPG main axes. In the favorable parallel alignment, the hydrogen atoms pointing toward the substrate (i.e., the relevant hydrogen atoms) interlock into the centers of the hexagons of the uppermost graphene layer. The hydrogen atom footprint of an *n*‐alkane follows a zigzag chain of the centers of adjacent graphene hexagons. In other words, the distances of the hydrogen atom footprints and the hexagon centers are minimized to a value close to zero [32]. In **1**, where solubilizing alkyl chains are absent, those hydrogen atoms of the tilted phenyl(ene) units that point toward the substrate do form a far more complex footprint (cf. Scheme 1), which also depends on the exact twist angle between the phenyl(ene) units. We propose that in the energetically favorable structure, alike in the Groszek model for *n*‐alkanes, for **1** the sum of the distances of all relevant hydrogen atoms to all relevant hexagon centers should reach a minimum. To test this hypothesis, we developed a Monte Carlo‐based registry optimization procedure [33]. It performs a screening of positions of the MSW relative to a graphene cutout, and for each position executes a query for relevant hydrogen atoms close to the nearest hexagon centers. The hydrogen atom footprints found within a 0.2 Å radius around each hexagon center are determined and summed up [34]. The molecule was placed above a graphene sheet in parallel orientation [35]. It was translated across the graphene surface in discrete steps, with each step involving a random lateral displacement sampled from a uniform distribution in the range of –0.5 to +0.5 Å. After each translational step, it was rotated around the surface normal in ±1° steps up to a maximum of ±30° to cover all non‐identical orientations with respect to the symmetry of HOPG [36]. For each such step, the sum of all hydrogen atom footprint centers of the molecule within a radius of 0.2 Å to the nearest hexagon centers of the graphene cutout was calculated. A complete run contains 50,000 of such steps as a standard value [37]. Then the position and orientation of the molecule on graphene with the largest sum of relevant hydrogen atom footprints was selected as a target geometry. For different input geometries (see Supporting Information), we determined the angle between the nonaphenylene axis of the molecule, which corresponds to the backbone orientation *c*, and the closest HOPG zigzag (or armchair) direction, *d* (or *d*’), both obtained from the STM measurement (Figure 2) [38]. The procedure was performed for conformers with different twist angles, *ϕ*, of the phenyl(ene) units, which were varied between 10° and 80° [39]. It yielded a correlation between the twist angle of the aromatic units in the molecule and the orientation of the nonaphenylene axis relative to the zigzag (or armchair) direction of the HOPG substrate, corresponding to the experimentally determined angle *γ*(*c*,*d*) from the STM measurements. For the enantiomer *P*‐**1** along the zigzag direction, these vary nonlinearly in a range from –7° to +4° for different twist angles (cf. Table S1) [40]. The experimental result of ±7° is calculated for a twist angle of 60°, which is similar to the value found for hexaphenyl benzene [41]. Aside from the orientations of –7° and +7° of the enantiomers *P*‐**1** and *M*‐**1** relative to the zigzag axis (Figure 3a,c), we also observed orientations of +11° and –11° relative to the armchair axis (Figure 3b,d). For *P*‐**1**/zigzag and *M*‐**1**/zigzag, we found 12 of the relevant hydrogen atoms (marked in yellow in Figure 3 a,c,e,g) with a footprint in a radius of 0.2 Å around the hexagon centers, while the other 72 relevant hydrogen atoms (marked in light, medium, and dark brown color) are closer to the carbon atoms of the graphene sheet [42]. A histogram of the spatial distribution of footprints is shown in Figure 4a. For *P*‐**1**/armchair and *M*‐**1**/armchair, we found 21 (9 more than for *P*‐**1**/zigzag or *M*‐**1**/zigzag) of the relevant hydrogen atoms with a footprint (marked in yellow in Figure 3b,d) in a radius of 0.2 Å around the hexagon centers, while the other 63 relevant hydrogen atoms (marked in light, medium, and dark orange/brown color) are closer to the carbon atoms of the graphene sheet, and a histogram is shown in Figure 4b. The histogram of the footprint reveals a large sum of hydrogen atom footprints in a hollow position (yellow bar), but a large number of relevant hydrogen atoms atop a graphene carbon as well (dark brown bar). This weakens the indicated preference of an orientation relative to armchair as only the raw registry score is higher than for the orientation relative to zigzag.

The experimentally obtained orientation of **1** relative to the underlying graphite gives therefore direct access to the overall conformation of all aromatics within **1**. Moreover, from the clockwise vs. counterclockwise direction of the rotation relative to the graphite axis, we can assign which enantiomer of **1** is observed in the respective STM image. In other words, the spatial resolution obtained by a combination of STM with registry optimization is far beyond the information obtained from the bright features in the STM images.

We successfully applied the registry optimization procedure to other new and previously reported MSWs with more complex geometries, and the results are discussed in detail in the Supporting Information. We expect that our procedure should be applicable to a broad range of molecules with a limited degree of freedom, containing spikes, such as surface‐oriented hydrogen atoms, and other substrates providing pockets. Further model refinements will also consider additional factors that influence the relative adsorbate‐substrate orientations, for example, the role of hydrogen spikes outside the 0.2 Å radius around the hexagon centers, and their exact orientations relative to the surface normal. Moreover, the efficiency of the method will allow us to extend the procedure to clusters of molecules to predict their supramolecular nanopatterns at solid surfaces.

## 3 Conclusion

We established a generalizable framework for predicting the adsorption geometry of large organic molecules on HOPG. By maximizing the contact between adsorbate hydrogen atoms facing toward the surface and the centers of the substrate hexagons in a Monte Carlo‐like approach, we achieve a physically intuitive registry model that avoids the need for expensive quantum chemical calculations. Inspired by historical models of *n*‐alkane adsorption, our method extends this concept to complex, π‐conjugated architectures. The correlation between theoretical registry scoring and experimental STM images enables a new form of structure determination at the solid/liquid interface with unprecedented interpretability. The results show that even subtle surface‐induced conformational locking—such as atropisomer freezing—can be resolved and predicted through geometric registry alone. Overall, geometric registry emerges as a versatile design principle for supramolecular systems at interfaces, enabling predictive control over surface‐confined self‐assembly.

## Author Contributions

**David A. Hofmeister**: conceptualization, methodology, investigation, visualization, writing – original draft, writing – review and editing, software. **Christian E. Selzer**: investigation, software, visualization, writing – review and editing, writing – original draft. **Laura zur Horst**: writing – original draft, investigation. **Simon C. Rickert**: investigation, writing – original draft. **Julia Kohn**: investigation, writing – original draft, visualization. **Andreas Hansen**: funding acquisition, supervision, writing – original draft, writing – review and editing. **Stefan‐S. Jester**: conceptualization, writing – original draft, writing – review and editing, project administration, supervision, visualization. **Sigurd Höger**: conceptualization, funding acquisition, writing – original draft, writing – review and editing, project administration, supervision.

## Funding

The authors gratefully acknowledge support from the Deutsche Forschungsgemeinschaft (DFG, RTG‐2591 “Template‐designed Organic Electronics‐TIDE”, Project ID 418404117 and Project ID 455731873).

## Conflicts of Interest

The authors declare no conflicts of interest.

## Supporting information



The authors have cited additional references within the Supporting Information [43, 44, 45, 46, 47, 48, 49, 50, 51, 52, 53, 54, 55, 56, 57, 58, 59, 60, 61, 62, 63, 64, 65]. The Graphene registry optimizer is available *via* https://github.com/Jester‐Group/GRegOptimizer. **Supporting File 1**: anie73190‐sup‐0001‐SuppMat.pdf.

**Supporting File 2**: anie73190‐sup‐0002‐movieS1.mp4.

**Supporting File 3**: anie73190‐sup‐0003‐movieS2.mp4.

## Data Availability

The data that support the findings of this study are available in the Supporting Information of this article.
