# Supplementary material for: Predictive Registry Optimization of Molecular Adsorbates on Solid Surfaces
Source: Angew Chem Int Ed Engl. 2026 Jun 13;65(34):e1693717. doi: 10.1002/anie.1693717 (PMC13480717; doi:10.1002/anie.1693717)
Supplement: Supplementary file 1 [file ANIE-65-e1693717-s002.pdf]

Supplementary Materials for

Predictive Registry Optimization of Molecular Adsorbates on Solid Surfaces

David A. Hofmeister,[a] Christian E. Selzer[b], Laura zur Horst[a], Simon C. Rickert,[a] Julia Kohn[b], Andreas Hansen*[b], Stefan-S. Jester*[a], and Sigurd Höger*[a]

[a] Kekulé-Institut für Organische Chemie und Biochemie
Rheinische Friedrich-Wilhelms-Universität Bonn
Gerhard-Domagk-Str. 1, 53121 Bonn; Germany
E-Mail: hoeger@uni-bonn.de; stefan.jester@uni-bonn.de

[b] Mulliken Center for Theoretical Chemistry
Rheinische Friedrich-Wilhelms-Universität Bonn
Beringstraße 4, 53115 Bonn; Germany
E-Mail: hansen@thch.uni-bonn.de

The PDF file includes:

Materials and Methods
Supplementary Text
Figs. S1 to S61
Tables S1 to S4

Other Supplementary Materials for this manuscript include the following:

Movies mov1, mov2

Contents

**1**	**General**	**S3**
1.1	Reagents and analytics	S3
1.2	Gel permeation chromatography (GPC)	S3
1.3	Scanning tunneling microscopy (STM)	S3
1.4	Computational details	S4
**2**	**Structures of 1 and 2 in the gas phase**	**S5**
2.1	Equilibrium geometries of **1** and **2**	S5
2.2	Concerted rotation of phenyl(ene) rings	S6
**3**	**Physisorption of saturated aliphatic hydrocarbons and all-phenylene MSWs 1 and 2**	**S7**
3.1	Physisorption of *n*-alkanes on graphite	S7
3.2	Physisorption of all-phenylene MSWs **1** and **2** on graphite	S8
**4**	**Registry optimization**	**S10**
4.1	General workflow	S10
4.2	Calibration	S13
4.3	Energetic evaluation of graphene hexagon centers as registry sites	S15
4.4	Adsorption height of MSWs on graphene	S18
4.5	Input structures for the registry optimization of **1** and **2** on graphene	S19
4.6	Registry optimization results	S23
**5**	**Additional STM images and (supra-)molecular models**	**S26**
5.1	Detailed STM discussion of **1**	S26
5.2	Additional STM images of **1**	S29
5.3	Packing Chirality of **1**	S31
5.4	STM discussion of **2**	S33
5.5	Additional STM images of **2**	S34
5.6	STM contrast variations of **1** and **2**	S35
5.7	STM discussion of **3**	S37
**6**	**Application of the workflow**	**S39**
6.1	Registry optimization of **2**	S39
6.2	Registry optimization of **3**	S41
6.3	Registry optimization of a reported system	S43
**7**	**Experimental vs. theoretical backbone/graphite orientations**	**S46**
**8**	**Synthesis**	**S47**
8.1	Synthesis of **1**	S47
8.2	Synthesis of **2**	S56
8.3	Synthesis of **3**	S68

1 General

1.1 Reagents and analytics

Reagents were purchased at reagent grade from commercial sources and used without further purification. All air-sensitive reactions were carried out using standard Schlenk techniques under argon. Reaction solvents (THF, methanol, 1,2-dichloroethane, piperidine, triethylamine, toluene, dimethylformamide) were dried, distilled, and stored under argon according to standard methods; workup solvents were either used in "p.a." quality or purified by distillation (dichloromethane, diethylether, cyclohexane). Prior to characterization and further processing, all solids and oils were dried at r.t. under vacuum. ^{1}H and ^{13}C NMR spectra were recorded on a Bruker Avance I 300 MHz, Bruker Avance I 400 MHz, Bruker Avance III HD 500 MHz Prodigy and Bruker Avance III HD 700 MHz Cryo (300.1, 400.1, 500.1 and 700.1 MHz for ^{1}H and 75.5, 100.6, 125.8 and 176.0 MHz for ^{13}C). Chemical shifts are given in parts per million (ppm) referenced to residual ^{1}H or ^{13}C signals in deuterated solvents. All NMR spectra were recorded at r.t. unless otherwise described. Mass spectra were measured on a Finnigan ThermoQuest MAT 95 XL (EI-MS), a Sektorfeldgerät MAT 90 (EI-MS), a Bruker Daltonics micrOTOF-Q (ESI-MS, APCI), a Bruker Daltronics autoflex TOF/TOF (MALDI-MS; matrix material: DCTB, no salts added) and an ultrafleXtreme TOF/TOF of the Bruker Daltonik company (MALDI-MS; matrix material: DCTB, no salts added). Thin layer chromatography was conducted on silica gel coated aluminium plates (Macherey-Nagel, Alugram SIL G/UV254, 0.25 mm coating with fluorescence indicator). Silica gel Kieselgel 60 (Merck, 0.040-0.063 mm) was used as the stationary phase for column chromatography. UV/vis absorption spectra were recorded on a Perkin Elmer Lambda 18 and fluorescence emission spectra on a Perkin Elmer LS-50B spectrophotometer using 10 mm quartz cuvettes.

1.2 Gel Permeation Chromatography (GPC)

Gel permeation chromatography (GPC) was performed in THF (HPLC grade, stabilized with 2.5 ppm BHT) at r.t. GPC analyses were run on an Agilent Technologies system at a flow rate of 1 mL/min using an IsoPump (G1310 A), a diode array UV detector (G1315B) and PSS columns (Polymer Standards Service, Mainz, Germany; 10^2, 10^3, 10^5 and 10^6 Å, 5 µ, 8 × 300 mm). All molecular weights were determined *vs* PS calibration (PS standards from PSS, Mainz, Germany).

For the preparative separation, a Shimadzu Recycling GPC (rec GPC) system, equipped with an LC-20 AD pump, an SPD-20 A UV detector and a set of three preparative columns from PSS (either SDV 10^3 Å, 5 µ, 20 × 300 mm or SDV preparative linear S, 5 µ, 20 × 300 mm) with precolumn (SDV, 5 µ, 20 × 50 mm) was employed. The system operated at a flow rate of 5 mL/min, THF, 35° C.

1.3 Scanning Tunneling Microscopy (STM)

Scanning tunneling microscopy (STM) was performed under ambient conditions (r.t.) at the solution/solid interface, using 1,2,4-trichlorobenzene (TCB) as solvent and highly oriented pyrolytic graphite (HOPG) as substrate. In a typical experiment, 3 µL of a saturated solution (or diluted solution) of the compound of interest was dropped onto a freshly cleaved HOPG substrate at elevated temperature (80 °C), kept at this temperature for 20 s, and allowed to cool to r.t. before the STM measurements were performed with the tip immersed into the solution. Bias voltages between –0.87 V and –1.85 V and tunneling current set points in the range of 4 pA to 101 pA were applied to image

the supramolecular adlayers shown here. The experimental setup consists of an Agilent 5500 scanning probe microscope that is placed on a Halcyonics actively isolated microscopy workstation. It is acoustically shielded with a home-built box. Scissors cut Pt/Ir (80/20) tips were used and further modified after approach by applying short voltage pulses until the desired resolution was achieved. HOPG was obtained from TipsNano (*via* Anfatec) in ZYB-SS and DS quality. All STM images (unless otherwise noted) were calibrated by subsequent immediate acquisition of an additional image at reduced bias voltage, therefore the atomic lattice of the HOPG surface is observed which is used as a calibration grid. Data processing, also for image calibration, was performed using the SPIP 5 (Image Metrology) software package. (Supra-) molecular modelling was performed using Wavefunction Spartan '18. Supramolecular models are based on equilibrium geometries of the respective MSWs which were obtained using molecular mechanics (based on the Merck Molecular Force Field (MMFF)) and a graphene monolayer with fixed atom positions as interaction partner.

1.4 Computational details

Geometry optimizations of the individual molecules in the gas phase were performed (unless noted otherwise) at the GFN-FF level of theory[26] using the freely available xTB 6.6.1 program package[16] with default convergence criteria 10^{-7} E_h for energies and 10^{-5} $E_h \cdot \mathrm{Bohr}^{-1}$ for gradients. The potential energy surface (PES) scans as well as the molecular dynamics simulations were performed using the ORCA 6.1.0 program package.[43]

Registry optimizations of individual molecules on a graphene cutout were performed using a Monte Carlo based algorithm. The respective code is written in python programming language and is available at:

The algorithm is added to the supporting information for reviewing only. It will be made available after the acceptance of the manuscript for publication by a Github link added here.

The code was built and executed on a Samsung Notebook with a 13th Gen Intel © Core i7-1355U, 1700 MHz, 10 core processor.

2 Structures of 1 and 2 in the gas phase

2.1 Equilibrium geometries of 1 and 2

Equilibrium geometries of **1** and **2**, obtained at the GFN-FF level of theory, are shown (Figure S1c and d) together with corresponding chemical structures (Figure S1 a and b).

In the gas phase equilibrium geometry of **1** (Figure S1c), all sixfold substituted benzene rings are aligned in parallel to the molecular plane and marked in grey. They are connected with each other *via* biphenylene units (blue), and the six outer rings are additionally substituted with three phenyl substituents each (blue). An alike equilibrium geometry was obtained for **2** (Figure S1d), where the outer six rings are substituted with 3,5-dimesitylphenyl units (blue) which, in comparison to the phenyl substituents in **1**, protrude even further out of the molecule plane.

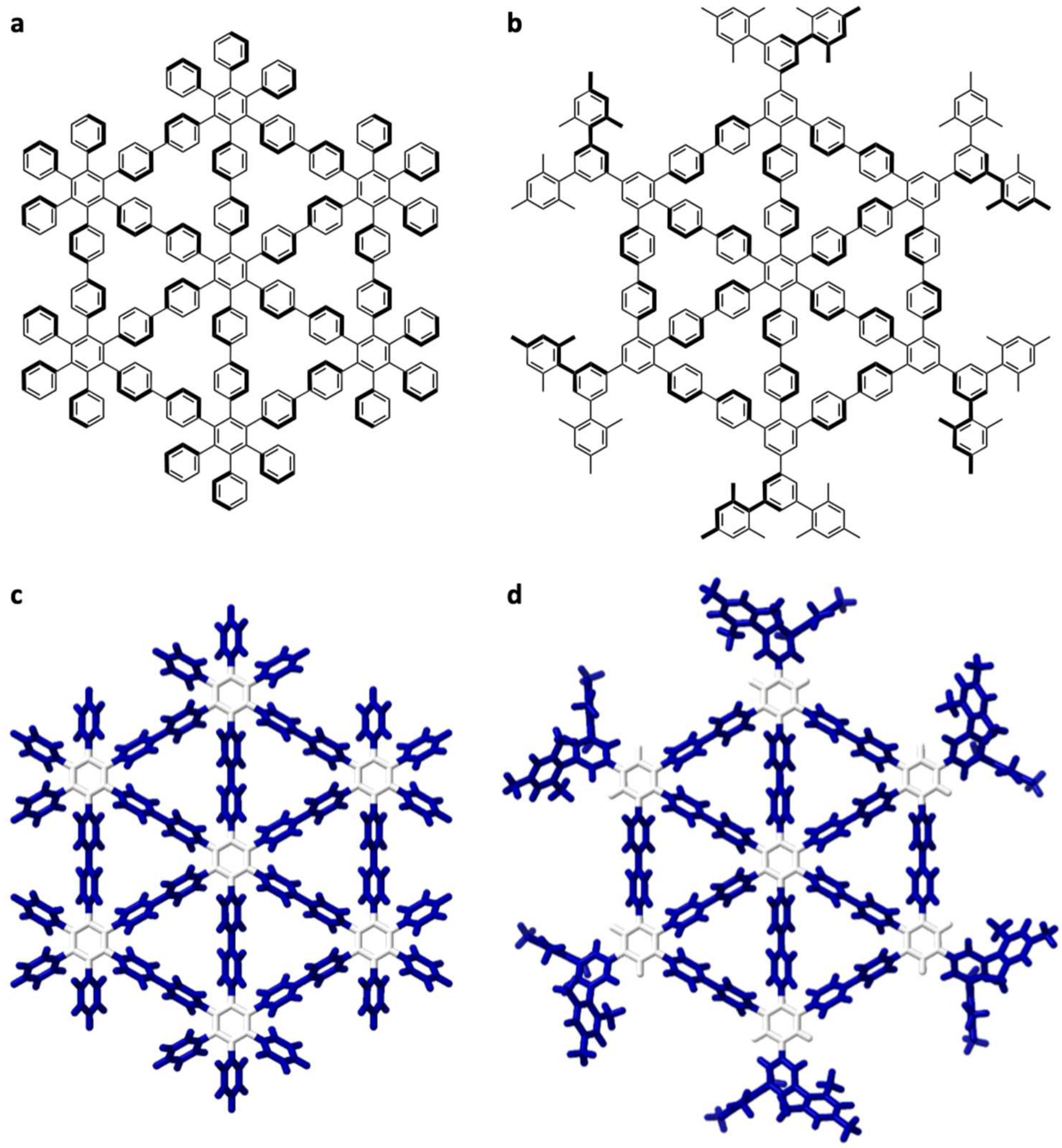

Figure S1 Chemical structures of (a) **1** and (b) **2**, as well as gas phase equilibrium geometries of (c) **1** and (d) **2**. The latter are obtained by geometry optimizations at the GFN-FF level of theory.

2.2 Coupled rotation of phenyl(ene) rings

The twisted orientation of the biphenyl units is expected to mechanically couple the orientations of the spokes due to the sterical constraint. To test this hypothesis a molecular dynamics simulation was performed (100 ps length, 1 fs stepsize, r.t., enhanced hydrogen mass of 4 amu (for stability reasons), no bonds constrained from breaking, GFN-FF level of theory, solution phase[44] (ALPBsolvation model with dioxane as solvent), Nosé–Hoover chains thermostat[45,46] with a time constant of 100 fs). As a starting geometry of the MD simulation, the *p*-phenylene unit marked in red in structure **A** (Figure S2) has been rotated around the two sigma bonds, so that it is aligned in parallel to the neighboring *p*-phenylene unit marked in blue (blue denotes orientation in the equilibrium geometry). From thereon, a series of 8 frames shows that two nearly complete isomerizations occur. For the full trajectory please see the accompanying movie "mov1.mp4".

Due to sterical constraints, the rotation of all 42 non-planar oriented rings occurs in a coupled fashion, similar to mechanically meshing gears in a gear box. In the simulation, isomerization can either be induced by perturbation (*i.e.* the rotation of a ring around the axis of the two sigma bonds, cf. conversion from structure **A** to structure **B**), or occur spontaneously (cf. conversion from structure **B** to structure **C**).

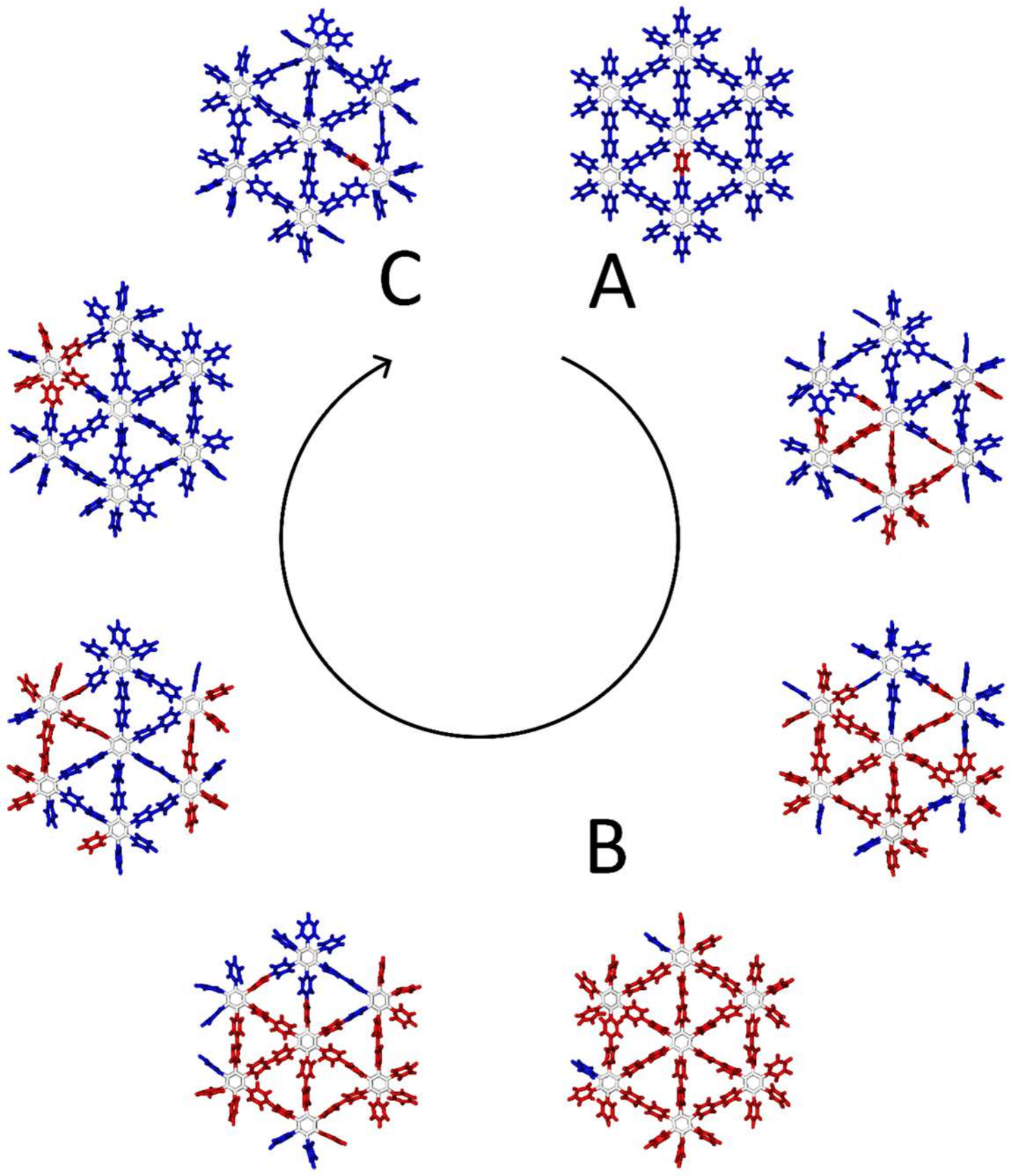

Figure S2 Molecular dynamics simulation of **1** at r.t. in the solution phase obtained by GFN-FF. Simulation was carried out for 100 ps with a time step of 1 fs and an enhanced hydrogen mass of 4 amu. Grey, blue, and red colors indicate aromatic rings aligned in parallel to the molecular plane, tilted in the direction given in the starting geometry, and the opposite direction, respectively.

3 Physisorption of saturated aliphatic hydrocarbons and all-phenylene MSWs 1 and 2

In this chapter we discuss the physisorption of *n*-alkanes according to the Groszek model to provide a scholarly introduction for the system used to develop and calibrate our method. We expand the Groszek model for the description of all-phenylene molecular spoked wheels (MSWs), which can be viewed as a set of oligo(phenylene) building blocks.

3.1 Physisorption of *n*-alkanes on graphite

Groszek proposed in 1970 a detailed molecular framework for understanding the selective adsorption of *n*-alkanes on graphite surfaces.[7] His motivation was that the adsorption should be relevant to effective lubrication of sliding surfaces of cast iron and graphite by mineral oils.[47] The model emphasizes the role of non-covalent interactions (mostly London Dispersion interactions) as the primary mechanism driving adsorption. Thereby, *n*-alkanes preferentially adopt a flat-lying conformation on the graphite basal plane, maximizing the molecular contact and enhancing interaction strength (cf. Fig. S3a for the example of $C_{16}H_{34}$).[a] The concept of selective adsorption is based on the structural compatibility between the periodicity of the graphite lattice and the *n*-alkane chains. As a result, the distances between the hydrogen atoms and the centers of its hexagons of the uppermost graphene layer of the HOPG substrate are minimized, as well as the system´s potential energy.[47] This is visualized in Fig. S3b, in which positions of the relevant hydrogen atoms are marked by black dots. The relevant hydrogen atoms are these that point towards the HOPG substrate. The adsorption energy (atom-atom interaction potentials) indicates that longer alkanes exhibit stronger adsorption due to increased surface contact. The sum of the surface contacts discussed for the Groszek model of *n*-alkanes may be viewed as a characteristic footprint of their specific geometry interlocking with the carbon hexagons of graphite.

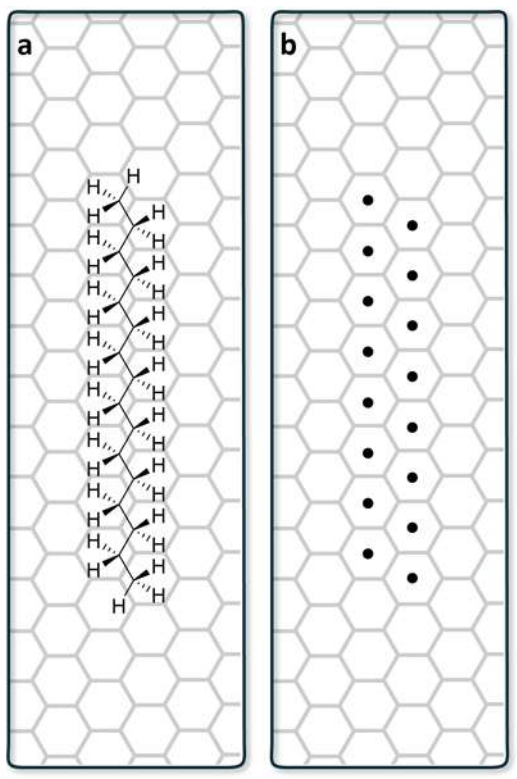

Figure S3 Schematical representation of (a) *n*-hexadecane ($C_{16}H_{34}$) adsorbed on the basal plane of graphite (uppermost layer schematically shown as grey hexagons) according to the Groszek model, and (b) contact areas (centers of the footprints, black dots) of hydrogen atoms and carbon hexagons in the basal plane.[a]

[a] Note that the wedge bar notation in Fig. S3a has certain limitations regarding the visualization of the positions of the H atoms relative to the centers of the graphite hexagons. The positions are, however, correctly shown as black dots in Figure S3b.

3.2 Physisorption of all-phenylene MSWs 1 and 2 on graphite

Similar to *n*-alkanes, all-phenylene MSWs may form characteristic footprints of surface contacts. One of the key questions is, whether MSWs also exhibit optimized surface contacts which can be described by an adapted, expanded Groszek-like model. Here we propose that also in MSWs the sum of distances of all relevant hydrogen atoms and the nearest carbon hexagon centers of the uppermost graphene layer of the HOPG substrate is minimized.

Considering a cutout of the backbone of **1** (cf. Fig. S5a), certain phenyl(ene) rings (some of which are separated by planar rings), are tilted relative to the molecule plane, leaving one edge of a ring closer to the graphite basal plane. There, two hydrogen atoms for each tilted ring are in close contact with the surface, while the other ones point towards the third dimension and should not play a role for the following considerations. The sum of the tilted rings may form a distinct footprint as a series of black dots, and an example of its possible orientation relative to the carbon hexagons is shown in Fig. S5b. However, it is unclear which would be the exact orientation (rotation and translation) relative to the graphite basal plane.

As described before, the phenyl(ene) rings can rotate in solution phase (cf. Fig. S2), therefore enantiomers cannot be separated. However, we expect that the phenyl(ene) rings do no longer rotate after physisorption to the HOPG substrate, therefore enantiomers of MSW **1** should not racemize after adsorption (for the full trajectory please see the accompanying movie “mov2.mp4”). A cutout of the corresponding enantiomorphic footprint of the MSW 1 and the respective backbone cutout are shown in Fig. S4c and d.

The backbone of **2** (cf. Fig. S1 and Fig. S4e) differs in parts from **1**, more specifically the ends of the corner units are different as they are substituted with a phenyl ring carrying with two mesityl (Mes) groups. While the hydrogen atoms of the inner biphenylene units will contribute to the footprint of **2**, (cf. Fig. S4f, black dots), the contribution of the two Mes groups is *a priori* not tangible. Again, two enantiomorphic footprints should be distinguished (cf. Fig. S4g and h).

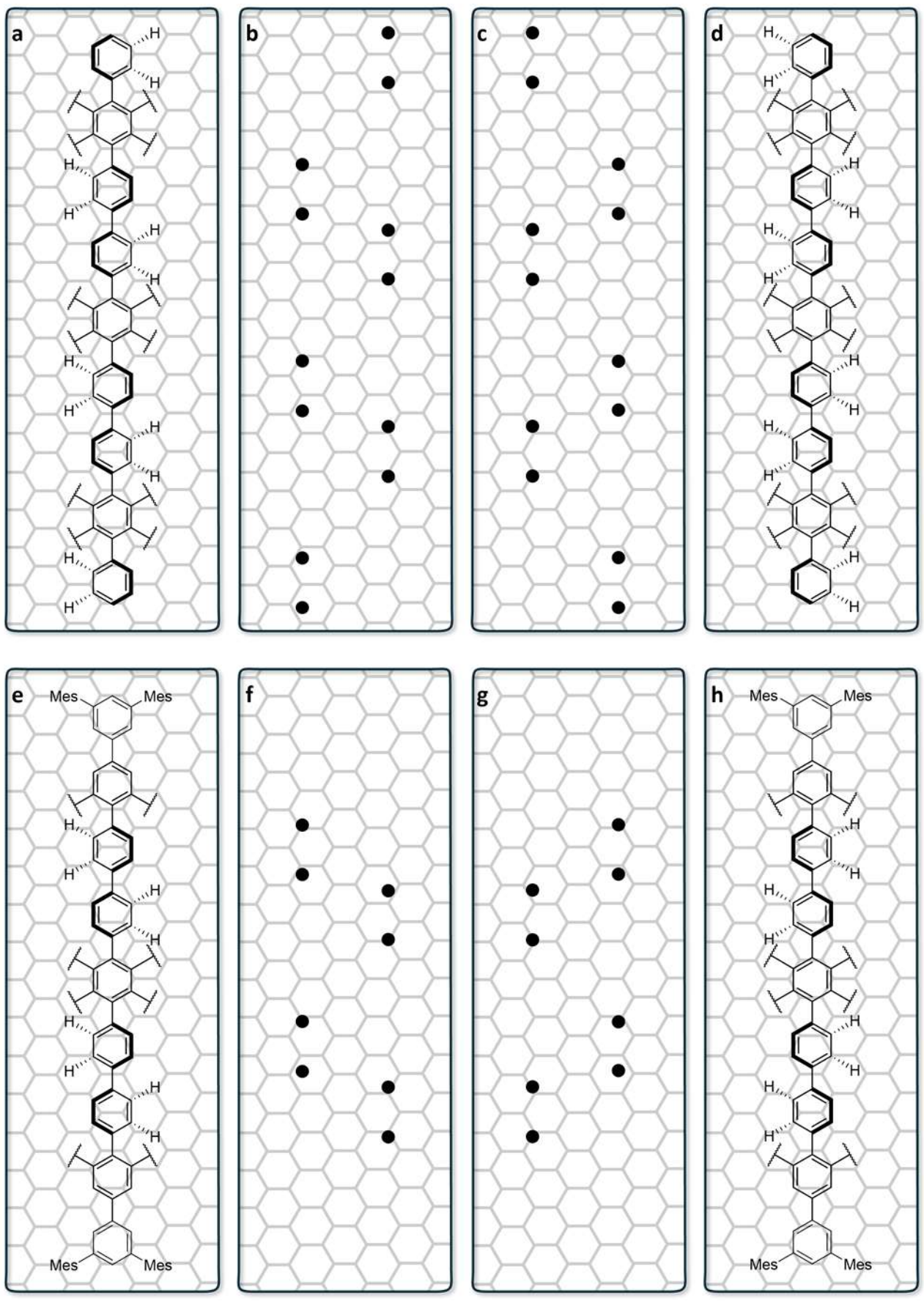

Figure S4 Schematical representation of enantiomorphic surface contact footprints of cutouts of MSWs (a)–(d) **1** and (e)–(h) **2**. The black dots show the positions of relevant hydrogen atoms, however without rotation and translation of the backbone relative to the graphite basal plane. The hydrogen atoms of the Mes groups of **2** are omitted here for clarity, but are considered after geometry optimization by GFN-FF (cf. Fig. S12) in the registry analysis (cf. chapter 4), and the results are shown in the Main Text.

In the next chapter, we will describe a method to elaborate the rotation and translation of MSWs relative to the graphite basal plane.

4 Registry optimization

To obtain insights into the preferable orientation of MSWs on graphene a Monte-Carlo based registry optimization algorithm is proposed. It is based on the Groszek model, which is for a first time applied to MSWs, where the amount of adsorbate hydrogens locking into the centers of the hexagons of the uppermost graphene layer of the HOPG substrate are maximized.

The respective code, called Graphene Registry Optimizer (short: GRegOptimizer) is written in the Python programming language and is publicly available at:

The algorithm is added to the supporting information for reviewing only. It will be made available after the acceptance of the manuscript for publication by a Github link added here.

4.1 General workflow

The registry analysis is implemented as a stepwise algorithm consisting of the following operations:

- (A) The program reads an input file containing the *xyz*-coordinates of all atoms.
- (B) Based on their *z*-coordinates, the atoms are assigned to three groups.
- (C) The graphene layer is then simplified into a two-dimensional grid of hollow-site coordinates representing the centers of the hexagons.
- (D1) The molecule is translated and rotated across the grid in the *xy*-plane while keeping the *z*-coordinate constant.
- (D2) A nearest-neighbor search is performed between the projections of the downward-facing hydrogen atoms of the molecule onto the graphene plane and the hollow-site coordinates.
- (D3) A registry match is counted when the *x*- and *y*-coordinates of a hydrogen atom fall within a 0.2 Å radius of a hollow-site coordinate.
- (D4) The configuration with the highest number of registry hits is tracked and stored.
- (E) Finally, a graphical output is generated to visualize the registry-matched positions.

A schematic illustration of steps (A) and (B) is provided in Fig. S5. After reading the *xyz*-coordinates, all atoms are first sorted by element type (in this case, only carbon and hydrogen).

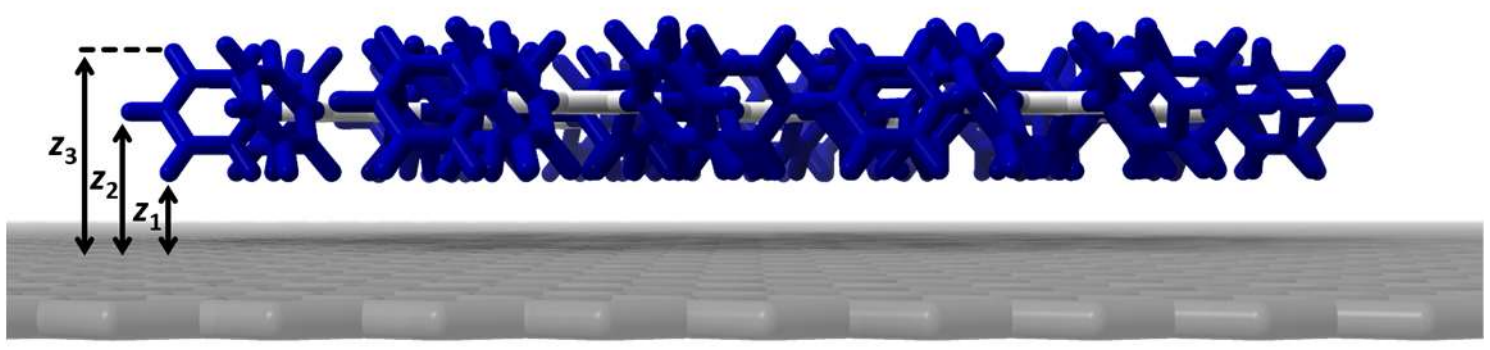

Figure S5 Schematic illustration of the separation procedure used to assign atoms to either the graphene substrate or the set of relevant hydrogen atoms, based on their respective *z*-coordinates. The hydrogen atoms located closest to the surface at height z_1 are automatically identified as relevant. Hydrogen atoms positioned at greater distances from the surface (*e.g.*, at heights z_2 or z_3) are excluded from the registry analysis.

The atoms are sorted into three groups:

(1) The graphene layer is identified by assigning all carbon atoms with a *z*-coordinate = 0 to the substrate group.
(2) The hydrogen atoms of the molecule that are oriented toward the surface are then identified *via* a nearest-neighbor search relative to the graphene layer. These atoms are assigned to the group of relevant hydrogen atoms.
(3) All remaining atoms are classified according to their element type and assigned to two separate lists (of hydrogen and carbon atoms).

Further processing of the graphene coordinates (step C) involves identifying the centers of all individual carbon hexagons (see Fig. S6). This is accomplished *via* a breadth-first search (BFS) algorithm, which traverses the list of neighboring carbon atoms to locate continuous six-membered cycles with defined C–C bond distances. The centers of these hexagons represent the hollow sites of the graphene layer and serve as registry targets for the simulation.

To facilitate further processing, the program inserts "dummy atoms" (here arbitrarily chosen as helium, He) at the calculated hollow-site positions with a fixed *z*-coordinate of 0. These additional He atoms are written to the original .xyz file and are used as reference points during the Monte Carlo-based registry analysis. In the simulation, this derived grid of hollow sites replaces the explicit carbon coordinates of graphene for efficiency and clarity (see schematic in Fig. S6, "x" represents the hollow sites).

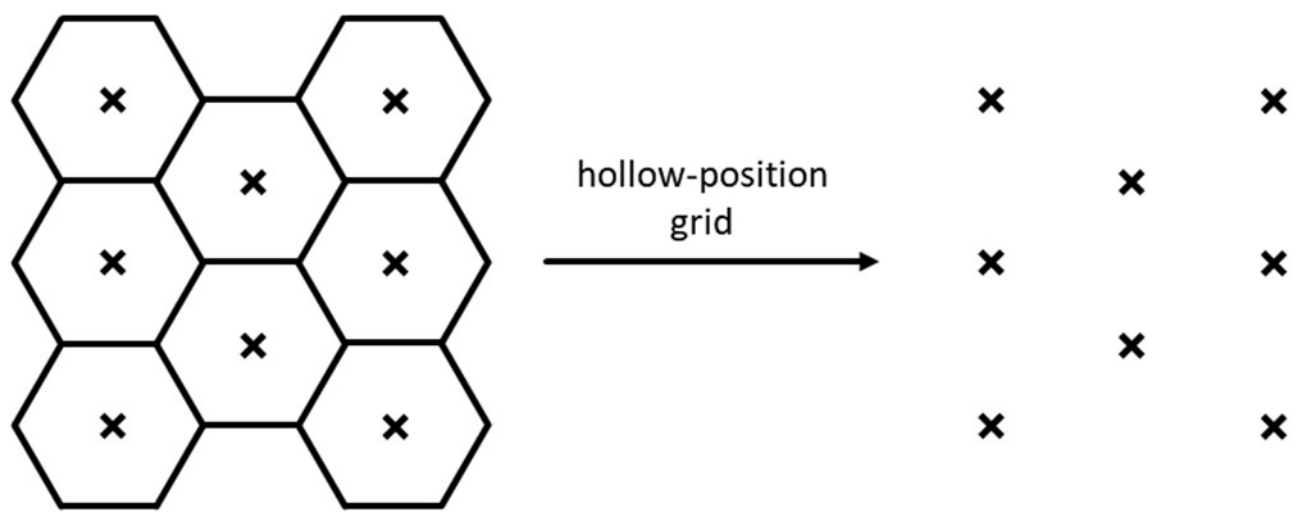

Figure S6 Schematic transformation of a graphene layer into a hollow-position grid using the hollow positions (marked with an x) of graphene.

This workflow reduces the workload of the nearest neighbor search during simulation as the grid only has to be generated once at the beginning of the simulation and the relevant hydrogen atoms do not have to compare their positions to multiple carbon atoms at each step.

At the start of the Monte Carlo simulation (D1), the molecule is placed at its initial position. During the first simulation step, it is randomly translated to a new location in the *xy*-plane using a defined step size. The parameterization of the simulation, including the specific step sizes for translation and rotation, is described in detail in the following chapter.

At the new location – and still within the same simulation step – the molecule is rotated around its center of mass within a defined angular range and using a fixed angular increment.

Subsequently, the hydrogen atoms of the molecule that are oriented toward the surface are compared to the hollow-site positions of the graphene layer using a nearest-neighbor search (D2).

Calculating Euclidean distances for every atom pair is computationally expensive. To improve efficiency, a KDTree data structure is used to identify the nearest hollow-site (represented by a “He” dummy atom) within a specified radius around each hydrogen atom. For each query point, a radius of $r = 0.2$ Å is applied (cf. Fig. S7), which defines a circular search area with a diameter of 0.4 Å. If a hydrogen atom is located within this region, it is counted as a registry match (D3); all other hydrogen atoms outside this radius are ignored.

For illustrative purposes, consider a single graphene hexagon (Fig. S7): the dummy atom is located at its center, and the circular search area defines the region within which matching hydrogen atoms can be found.

To provide further insight into the registry quality, the matching hydrogen atoms are sorted into categories based on their distance from the center of the hexagon. These are visualized using color shading from light to dark, corresponding to increasing distance intervals ranging from 0 to $a_0/\sqrt{3}$, where $a_0 = 2.46$ Å is the graphene lattice constant.

In the case of a single hexagon, this procedure would result in a maximum of one registry match.

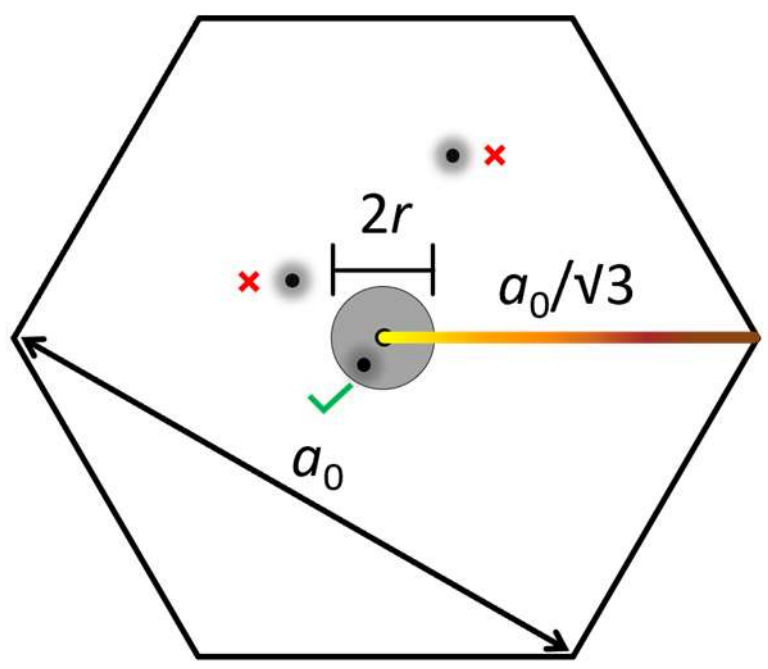

Figure S7 Schematic visualization of match detection and distance sorting within one graphene hexagon. The radius r is typically $r = 0.2$ Å, the unit cell vector in graphene is $a_0 = 2.46$ Å. Pictograms (green and red) are used to display successful and unsuccessful match detection, respectively.

Since multiple registry matches may occur during a single event, the total number of matches is summed over the entire molecule. This workflow is repeated for a defined number of events (n, typically $n = 50{,}000$), and the configuration(s) with the highest match count are continuously tracked and stored (D4). For each of these top configurations, the event number, rotation angle, and molecular position are recorded.

To avoid edge effects and ensure continued sampling near the system boundaries, periodic boundary conditions were implemented for both translational and rotational moves.

After completion of the simulation, all configurations with the highest registry count are visualized (E). In these plots, the matched hydrogen atoms are shown as colored dots, corresponding to their radial distance from the center of the associated graphene hexagon. To visualize the registry footprint, the full graphene layer – rather than the simplified hollow-site grid – is depicted, with carbon atoms shown as black dots and bonds as black lines.

A schematic summary of the simulation workflow is provided in Fig. S8.

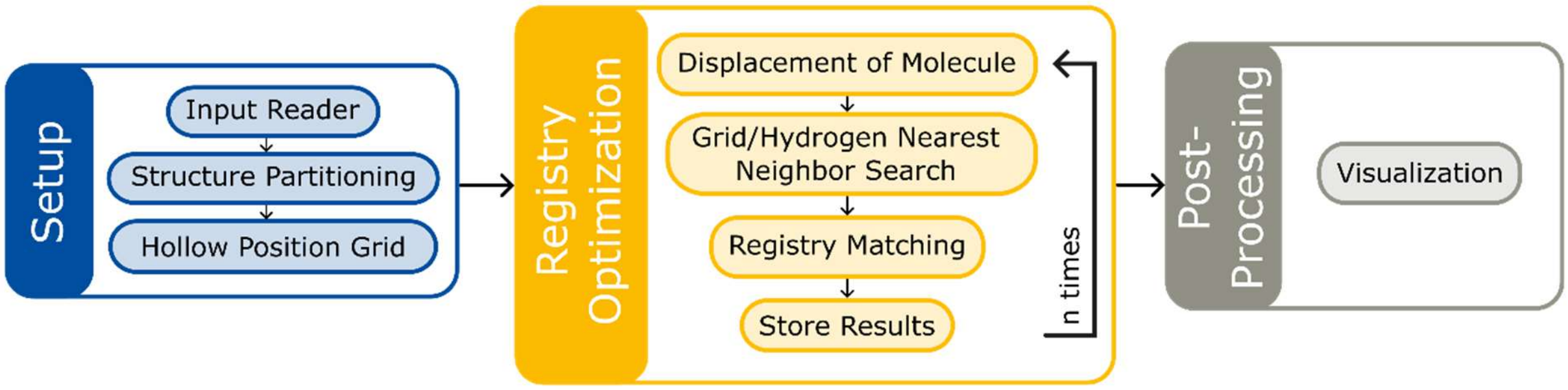

Figure S8 General workflow of the registry optimization code.

Throughout the simulation, all atoms of the molecule are moved/rotated synchronously. Even though they are not relevant for registry match counting, this allows generating .xyz files containing the registry optimized structures on a trimmed graphene cutout for further use.

4.2 Calibration

To test and calibrate the registry optimization method, we reproduced the preferential adsorption orientation of a flat-lying alkane ($C_{16}H_{34}$) on a graphene cutout. The molecular geometry was first optimized using the GFN-FF method on a fixed graphene sheet. The alkane was then manually rotated out of its preferential adsorption orientation by a few (*e.g.* 11) degrees.

We conducted multiple optimization runs in which key parameters were systematically varied: the translational step size, the rotational step size, and the tolerance radius for the nearest-neighbor search between hydrogen atoms and hollow-site positions. We found that a translational step size in the range of ±0.2 – 0.5 Å (drawn from a uniform distribution) was sufficient to adequately sample the surface and capture registry repetitions. The use of a uniform distribution enhances sampling resolution by giving smaller steps equal probability.

For rotational sampling, a step size of 1° within a range of ±30° around the initial orientation was found to provide sufficient angular resolution without incurring excessive computation time. Smaller increments, such as 0.1°, significantly increased the computational effort without yielding substantial benefits. (Note that to cover all non-identical orientations with respect to the symmetry of HOPG, either a range of ±30° around any reference direction, or ±15° around *e.g.* both the zigzag and armchair directions may be sampled.)

Nearest-neighbor accuracy was controlled by the choice of a threshold radius in the *xy*-plane. This radius must be sufficiently small to prevent double-counting but sufficiently large to allow for minor mismatches due to lateral displacement. A threshold of 0.2 Å was found to strike a good balance. This tolerance is physically justified, as small axial shifts of alkyl chains have only a minor effect on adsorption energy, and misaligned chains may still favor flat-lying arrangements along the graphene zigzag direction, as described in the Groszek model (cf. chapter 3.1).[10]

Applying these optimized parameters to the test case (Fig. S9a) resulted in the registry output shown in Fig. S9b, successfully reproducing the expected adsorption behavior. These parameters were subsequently used as calibration for the registry analyses of **1** and **2**.

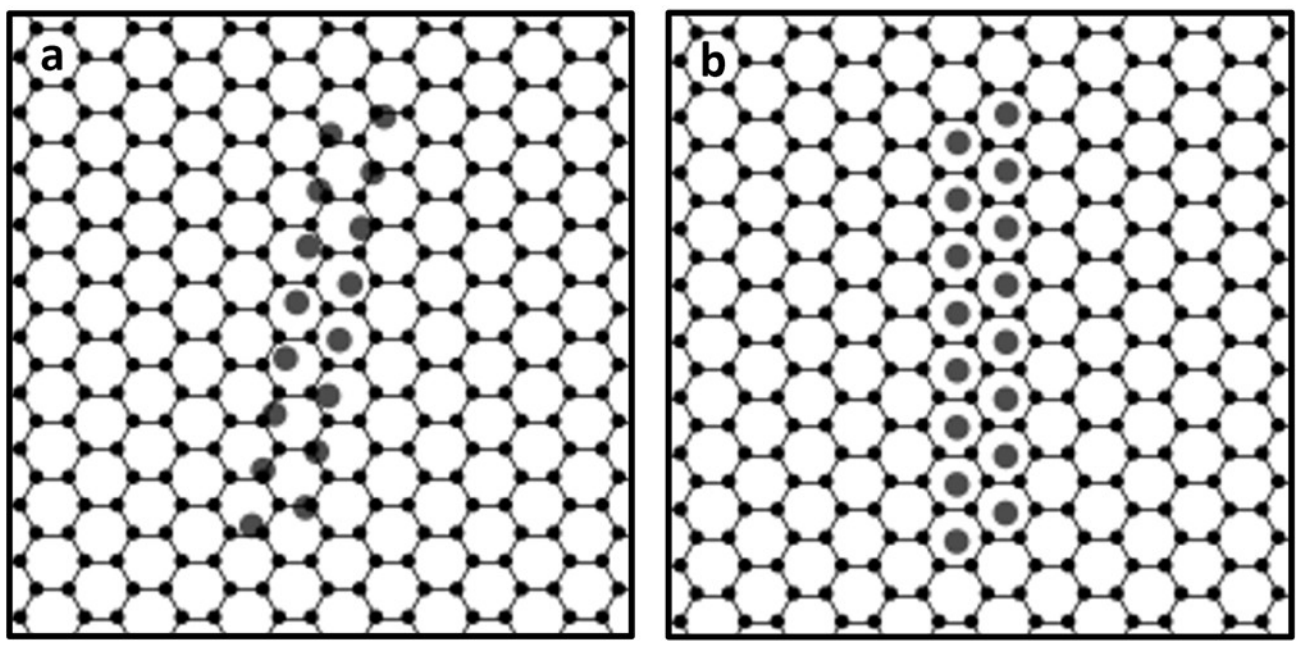

Figure S9 Calibration of the registry optimization program. 2D plots of a flat-lying alkane $C_{16}H_{34}$ on graphene at (a) input location and (b) registry optimized position. Only the relevant hydrogen atoms are shown and represented by black dots.

4.3 Energetic evaluation of graphene hexagon centers as registry sites

Graphene carbon hexagons exhibit three high symmetry binding sides. The first is located atop a carbon atom (“top” or “T-site”), the second atop a C–C bond (“bridge” or “B-site”) and the hexagon center (“Hollow” or “H-site”). The three adsorption sites are schematically visualized in Figure S10.

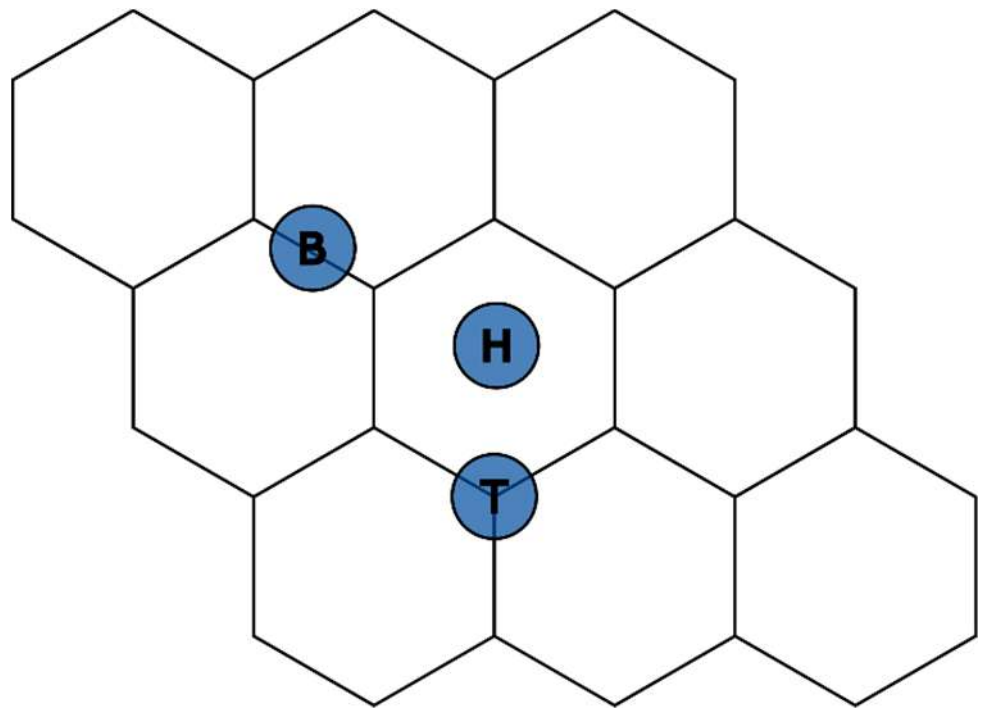

Figure S10 Schematic representation of three high symmetry binding sites of graphene indicated by H (center of a hexagon), T (atop a carbon atom) and B (atop a C–C bond).

Numerous theoretical studies have been reported investigating physisorption phenomena of small adsorbates on benzene and graphene.[48,49,50,10] The strong interest in these interactions is reflected by the large number of different levels of theory that have been used, ranging from simple Lennard-Jones type interaction potentials[51] over Machine Learning potentials[52] over DFT[53] to studies using high level ab initio methods.[54] It has been found that for physisorption phenomena the H-site is often the preferred one.

This type of CH – π interaction is a well-known NCI motive.[55] Although this type of hydrogen bond is smaller in interaction strength than typical hydrogen bonds, as they are present in e.g. water, they can have a guiding influence on the structure and assembly of molecules.[56,57]

We were intrigued by the question whether the H-position represents also an energetic minimum for the case of a CH-π interaction with the adsorbate being non-aliphatic. Thus, we conducted relaxed potential energy surface (PES) scans of benzene adsorbed on a cutout of graphene using the efficient r^2SCAN-3c method[58] with a step size of 0.1 Å. r^2SCAN-3c was chosen because of its demonstrated accuracy especially for geometries and conformational energies.[59,59,60]

The chosen system serves as a suitable model system, approximating the MSW. Two scans were conducted, first the benzene molecule was placed atop a carbon atom vertical to the graphene sheet with one hydrogen pointing toward the graphene sheet. Then it was translated to the carbon atom across hovering over the hollow position. The second scan was conducted analogous to the first one only that the movement was from the B-site to the opposite B-site. A visualization of these two approaches together with their corresponding results are given in Fig. S11a and b.

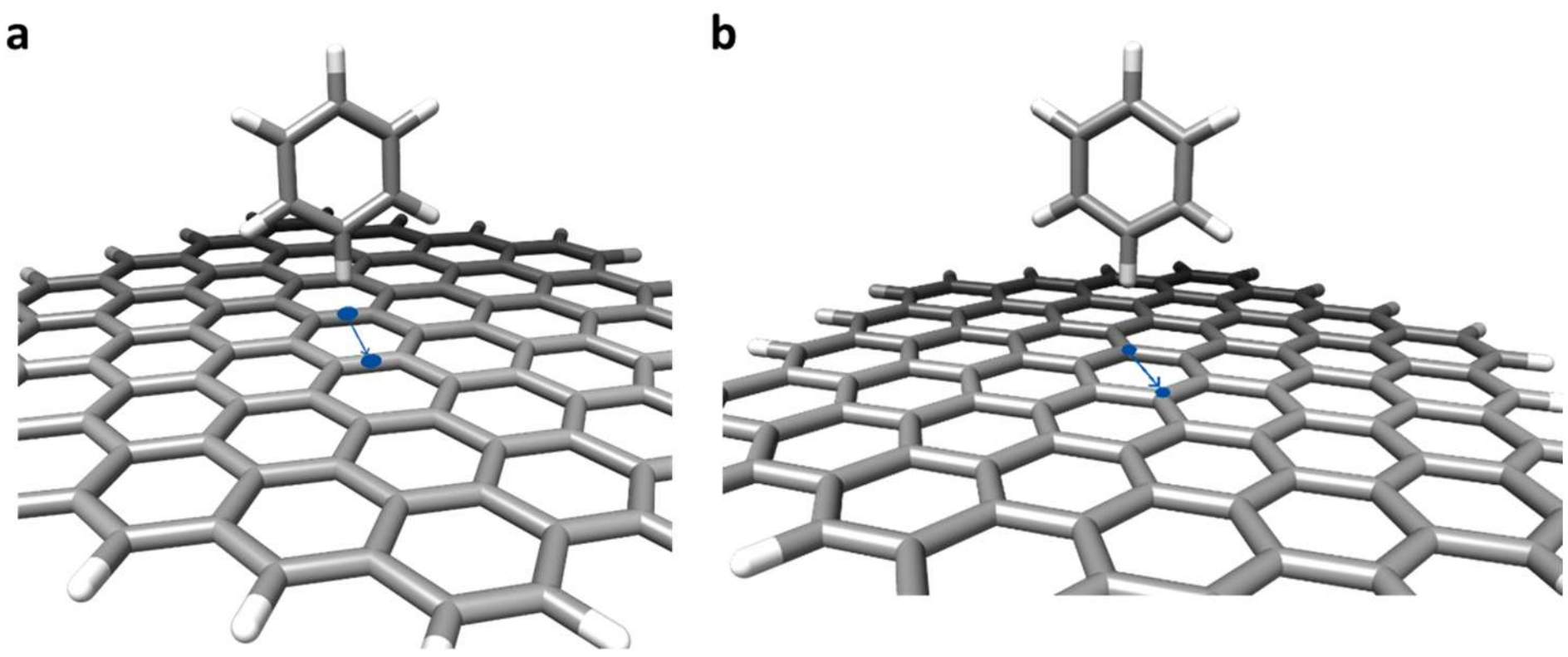

Figure S11 (a),(b) Graphical representation of two relaxed PES scans: (a) Scan from B to B site and (b) scan from T to T side. In both cases the benzene molecules were kept orthogonal to the graphene plane.

The results are in accordance with previous literature results. The PES shows an energetic minimum located at the H-site (cf. Fig. S12). This further supports the idea of the GRegOptimizer algorithm and the chosen thresholds, as the hexagon center of graphene represents an energetic minimum. The preference of the H site is very small for one CH-π interaction (approximately 0.4 – 0.5 kcal mol^{-1}), however the maximization of the C–H-H-site interactions throughout oligo-Phenyl(ene)s can lead to a non-negligible stabilization.

Looking at the spatial extend of the calculated PES profiles this further supports the chosen value for the cutoff radius of 0.2 Å in the registry algorithm as the change in energy is still well below 0.1 kcal mol^{-1}.

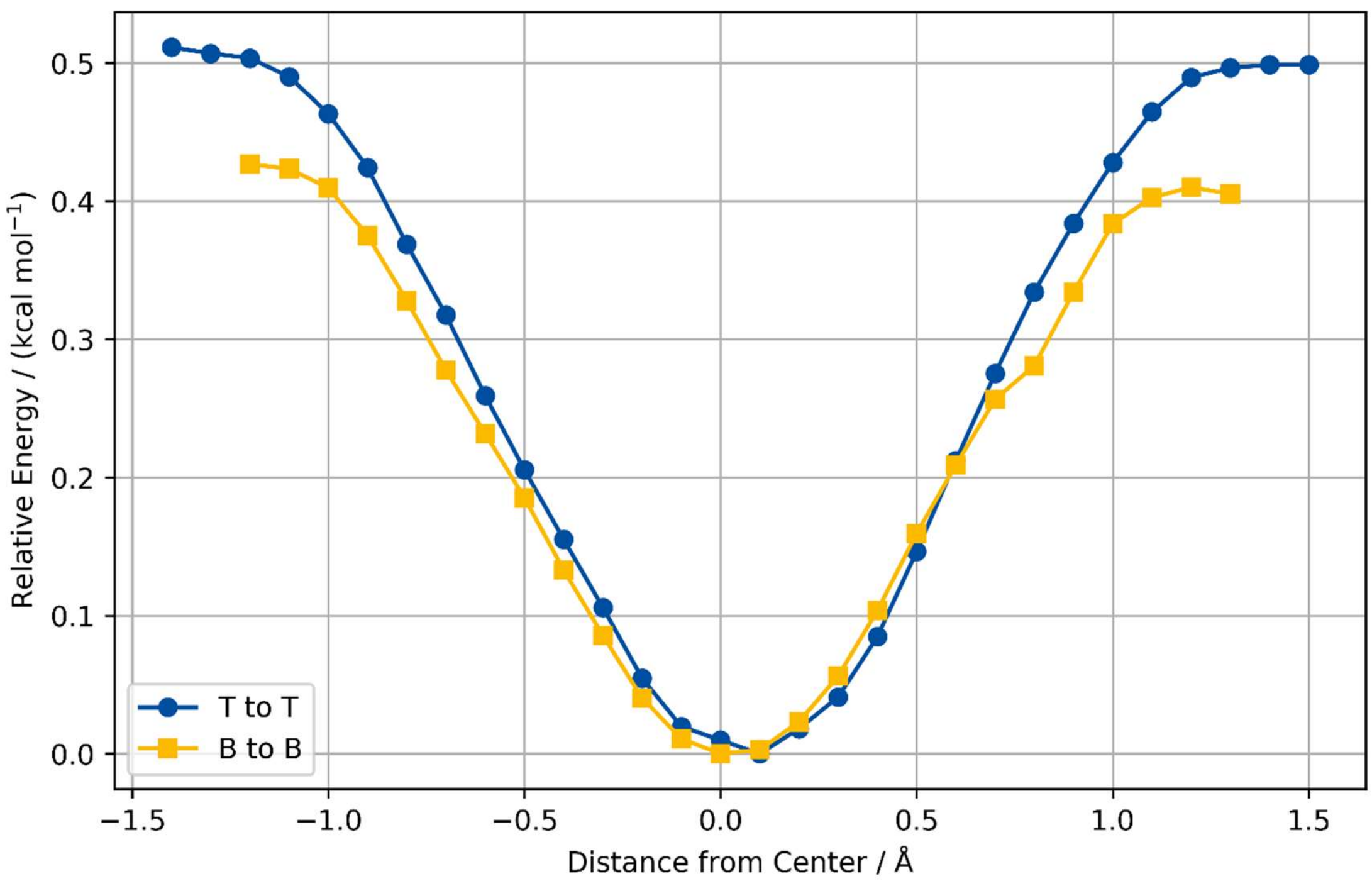

Figure S12 Results from relaxed PES scans probing the CH-π interaction of a benzene molecule adsorbed on a graphene sheet cutout. All results were calculated on the r²SCAN-3c level of theory.

4.4 Adsorption height of MSWs on graphene

In the GRegOptimizer algorithm the adsorption height in *z*-direction is neglected as registry optimization only considers an *xy*-projection of molecular coordinates. However, the observed molecular registry of the adsorbed MSW **1** requires a stable adsorption geometry of the wheel in close proximity to the graphene sheet. Therefore, a potential energy surface scan varying the vertical distance of the wheel to the sheet was performed to determine a physically reasonable adsorption height. The results are shown in Figure S13.

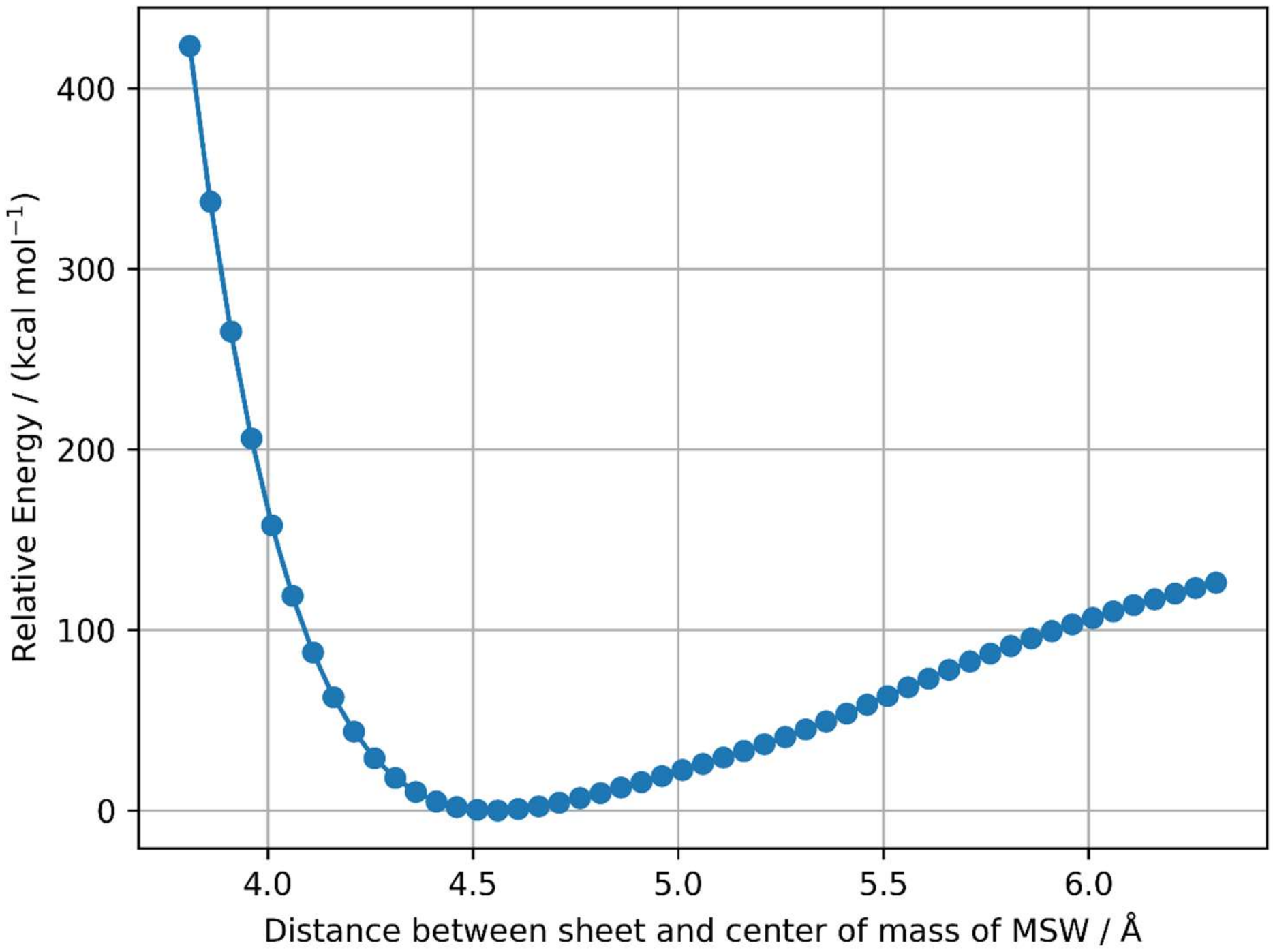

Figure S13 Unrelaxed PES scan using GFN1-xTB as implemented in xTB 6.6.1. The distance between the center of mass of the MSW and the plane defined by the graphene sheet was scanned.

We observed an energetic minimum at a vertical distance of 4.5 Å between graphene and the MSW.

4.5 Input structures for the registry optimization of 1 and 2 on graphene

The input structures for registry optimization can be prepared using either quantum chemistry software or molecular modelling. In general, suitable quantum chemical methods should be able to handle sufficiently large systems, correct for competing interactions, such as hydrogen bonds, and also implement solvation models, if required.

Two principal approaches were employed to generate the structures investigated here: (i) geometry optimization of the molecule on a fixed graphene surface using force field methods (e.g., GFN-FF) with implicit solvation models (e.g., ALPB), and (ii) idealization of the molecular backbone by adjusting defined twist angles between phenyl(ene) units.

For **1**, both strategies were applied to investigate (i) the compatibility of the registry optimization algorithm with force-field-optimized geometries, and (ii) the influence of phenyl(ene) torsion angles on registry outcomes. First, the molecular geometry of **1** was pre-optimized in the gas phase using GFN2-xTB, followed by a geometry optimization on a fixed graphene sheet using GFN-FF (both with implicit solvation (ALPB, toluene)). This yielded a conformation with an average twist angle of 59.5° between adjacent phenyl(ene) units.

To assess registry alignment, both enantiomers (***P*-1** and ***M*-1**) were manually placed on a graphene cutout and oriented either along the zigzag or the armchair axis, resulting in four distinct input structures.

Specifically, for the idealized geometry we compared the average twist angle (59.5°) with the typical value in hexaphenylbenzene (HPB), which is known to adopt twist angles around 60° depending on substitution patterns.[61,62] Based on this agreement, we constructed idealized molecular backbones with a uniform torsion angle of 60° for both enantiomers. We constructed the MSW from hexaphenylbenzene units that were geometry optimized using a graphene cutout as interaction partner at the GFN-FF level of theory in order to manually idealize twist angles. The so constructed MSW was again oriented along the zigzag and armchair axes, yielding another set of four input structures for the registry optimization. The corresponding geometries are shown in Fig. S14: (a) and (b) for ***P*-1** and ***M*-1** along the zigzag axis, and (c) and (d) along the armchair axis.

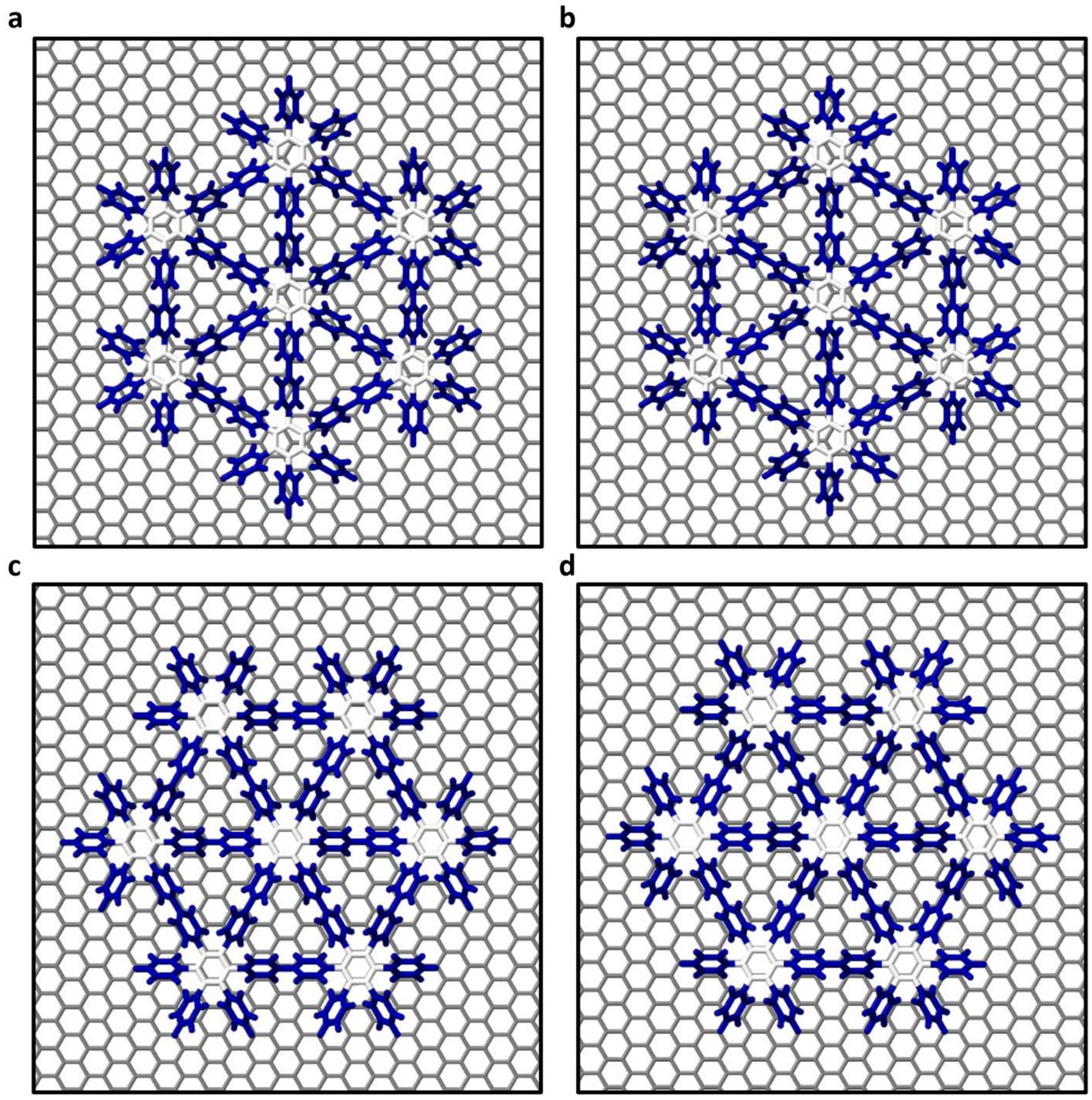

Figure S14 Idealized backbones of both isomers of **1** on graphene for the registry optimization. Both isomers ***P*-1** and ***M*-1** oriented along the (a),(b) zigzag and (c),(d) armchair axis. The twist angles are fixed at a uniform value of 60°.

To investigate how variations in the twist angle affect the registry-optimized configurations, we generated additional input structures for both enantiomers by systematically adjusting the twist angle. The relevant twist angle Φ_1 is defined as shown in Fig. S15a, using biphenylene subunits. Starting from hexaphenylbenzene (HPB) motifs with different preset twist angles Φ_2 (cf. Fig. S15b), we constructed a series of molecular backbones for both isomers of **1**.

Φ_1 was varied within the range of 10° to 80°. For the low-angle regime (10° – 40°), we used increments of 10°, while around the expected equilibrium angle of $\Phi_1 = \Phi_2 = 60°$, a finer resolution of 5° steps was applied (*i.e.*, in the range of 40° – 80°). All resulting molecular backbones were aligned along the zigzag direction of a graphene sheet and used as input for registry optimization.

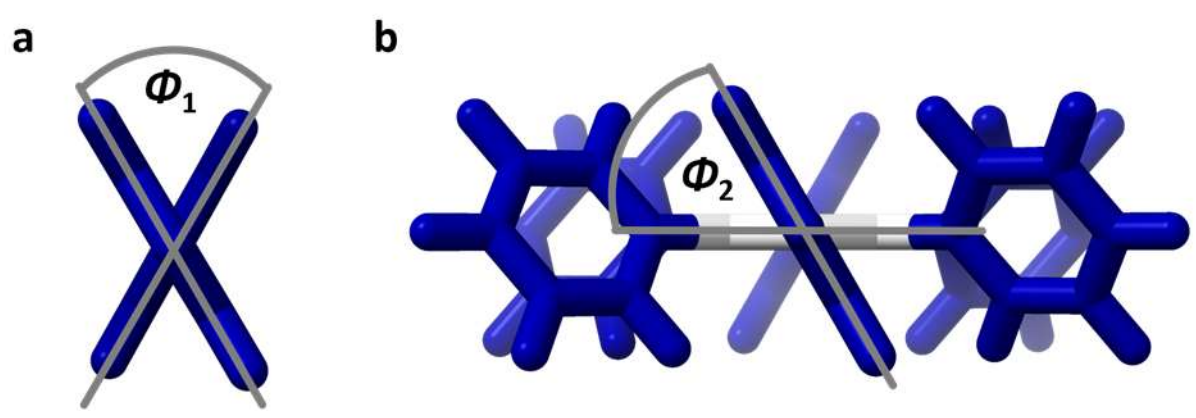

Figure S15 Side views of (a) biphenylene and (b) hexaphenylbenzene MSW subunits. The twist angles of biphenylene units Φ_1 were manually set to obtain input structures of isomers of **1**. Φ_1 was adjusted by manually fixing the angles of Φ_2.

Regarding the input structures of **2**, again two isomers ***P*-2** and ***M*-2** may form on graphite. Unlike **1**, **2** carries dimesitylphenyl groups which pack densely in the nanopatterns formed. These groups are

sterically demanding but at the same time have degrees of freedom at the methyl groups. An idealization of the backbone is not suitable here as the relative positioning of the subunits of **2** is more complex than for **1**. Therefore, we solely relied on the GFN-FF method to model the backbone structure. We placed one molecule of **2** on a graphene sheet with fixed coordinates and performed a geometry optimization with implicit solvation. Then, both isomers of **2** were rotated within the substrate plane so that the backbone is oriented along the zigzag axis and used the so-obtained systems as inputs for registry optimization within a rotation range of ±30° (cf. Fig. S16a and b).

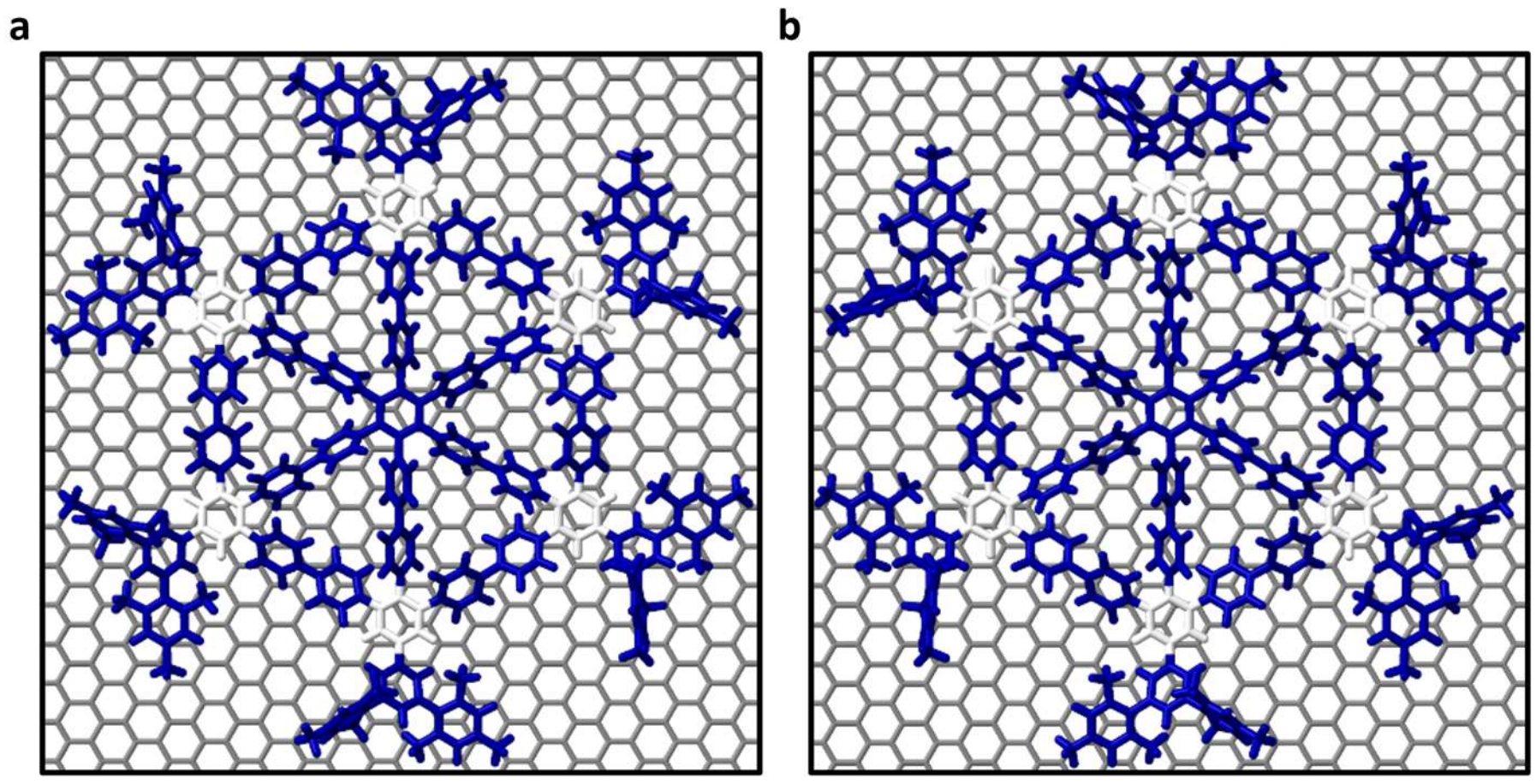

Figure S16 Geometry optimized backbones of both isomers (a) ***P*-2** and (b) ***M*-2** oriented along the zigzag axis of a graphene sheet.

Due to steric effects of the dimesitylphenyl units, some parts of **2** adopt a larger *z*-value relative to the graphene plane after geometry optimization. Especially the rim is lifted from the surface as the phenylenes there are closer to the bulky end groups (cf. side view of the input structure of ***P*-2**, Fig S17).

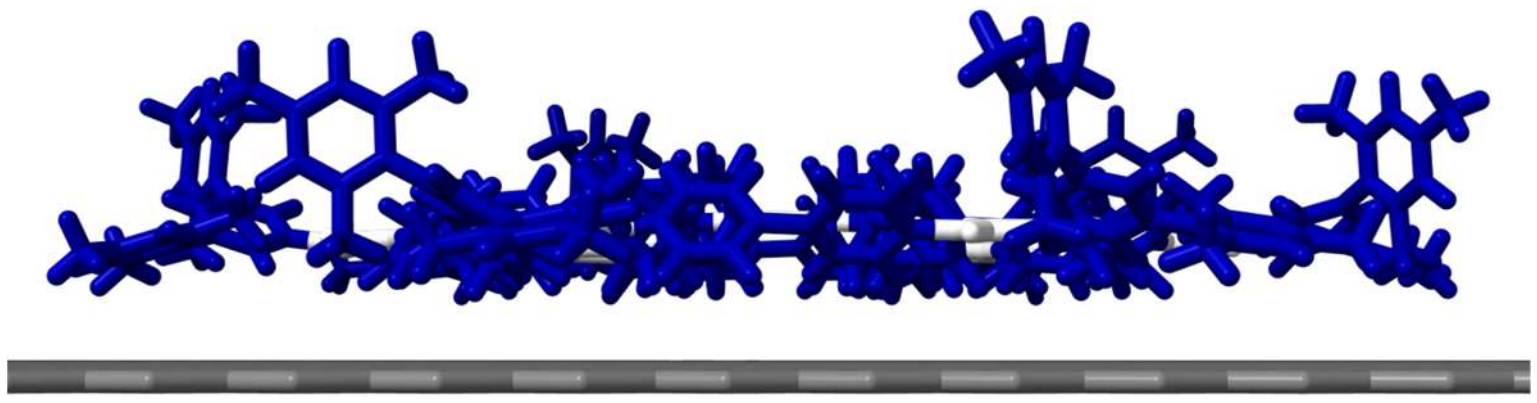

Figure S17 Side view of the input structure of ***P*-2** shown in Fig. S16a.

During registry optimization, the *z*-coordinates of the relevant hydrogen atoms are kept constant and therefore do not influence the resulting configurations with the highest registry match count. The selection of relevant hydrogen atoms of **2** is based on their downward-facing orientation relative to the planar graphene hexagons (indicated in light gray in Fig. S16 and S17). However, within this group of hydrogen atoms, their distances from the surface vary. The distribution of their *z*-values is visualized using a yellow-to-blue color gradient in Fig. S18, shown as (a) a top view and (b) a side view of the relevant hydrogen atoms on the graphene layer. Taking ***P*-2** as an example, the hydrogen atoms closest to the surface are located near the center of the molecule (spoke segments). In contrast, the molecular rim is lifted away from the surface due to steric interactions with the dimesitylphenyl groups. Most downward-facing hydrogens on these peripheral groups are positioned even farther from the surface. Although all relevant hydrogen atoms are oriented downward, we expect that those located in the spoke segments contribute more significantly to the registry of **2**.

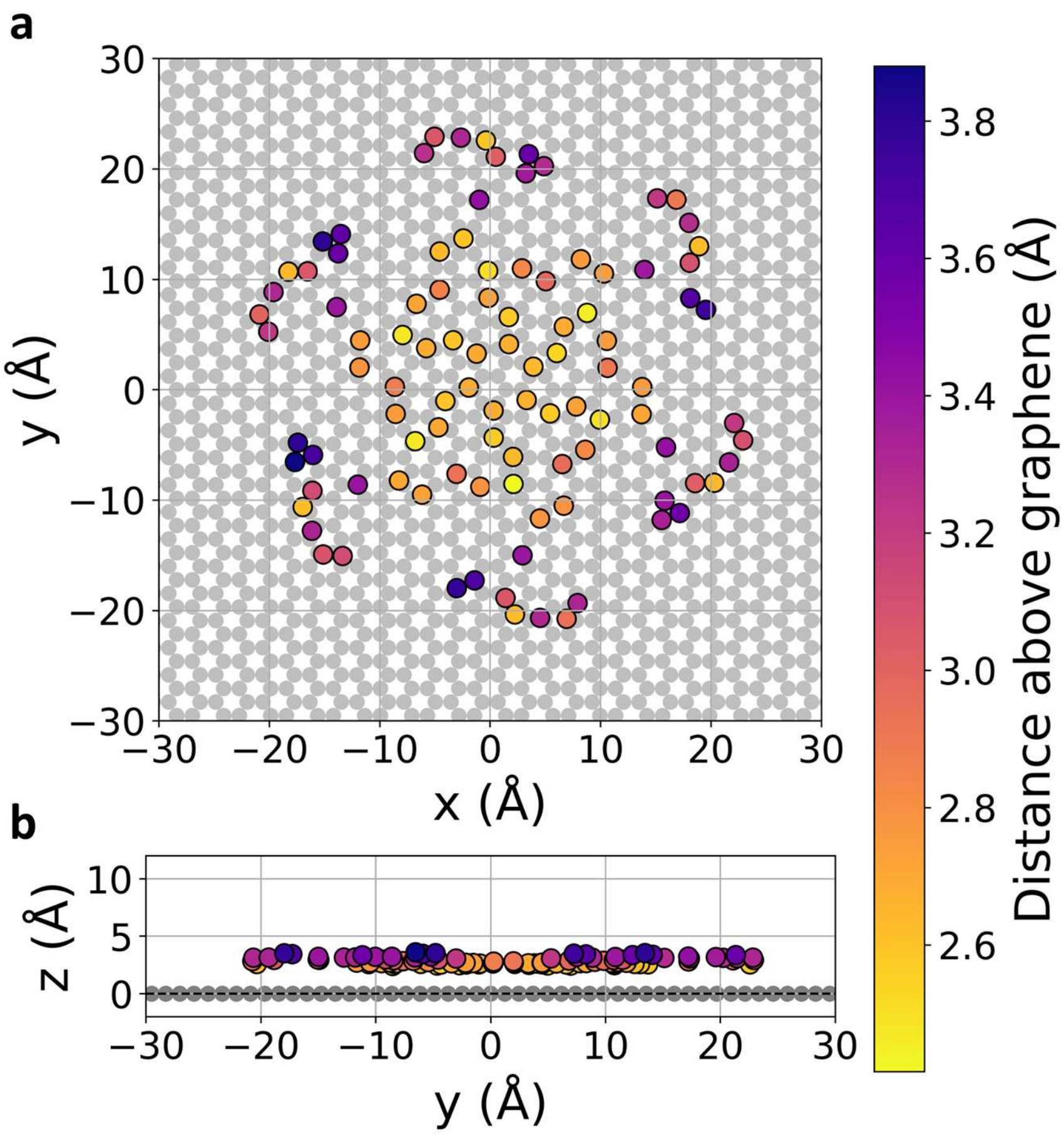

Figure S18 Distribution of relevant hydrogen atoms of ***P*-2** within the input structure for registry optimization according to their distances above the graphene layer. For ***M*-2**, the distribution is mirrored and not shown here.

4.6 Registry optimization results

Results of registry optimizations of the idealized backbones of **1** with uniform twist angles of 60° are discussed in Fig. 3 of the Main Text. Backbones obtained from geometry optimization yielded similar rotations of the nonaphenylene axes relative to zigzag or armchair (–6° for ***P*-1**/zigzag, +11° for ***P*-1**/armchair, +6° for ***M*-1**/zigzag and –11° for ***M*-1**/armchair). Both approaches to obtain input structures led to registry optimization results that are in good agreement with the experimentally observed rotations relative to the respective axes. To discuss the influence of twist angles in more detail, we investigated the effect of Φ_1 on the optimization results. We varied Φ_1 manually in a stepwise fashion and performed registry optimizations (see chapter 4.1). We conducted this study for the registry of the ***P*-1** (and ***M*-1**) isomer in a range of ±15° relative to the zigzag axis. The results shown in Table S1 suggest that twist angles deviating from Φ_1 = 60° in the idealized backbones do not accurately reflect the experimental results.

Table S1 Results of performing registry optimizations with varying twist angles Φ_1. The main result using an idealized twist angle is underlined.

Φ_1 [°]	Rotation rel. to zigzag [°]
10	
	-1
20	
	+2, -3
30	
	-5
40	
	-5
45	
	-5
50	
	-4
55	
	-2, -4
60	
	-7
	+1, -3
	+4, -3
70	
	+4
75	
	+4
80	

To evaluate the strength and orientation of registry matching for **1** (defined as hydrogen-to-center distance ≤ 0.2 Å), we plotted the maximum number of registry matches identified for each rotation angle relative to the zigzag and armchair axes (within a range of –15° to +15°) as bar diagrams (cf. Fig. S19).

For the zigzag orientation, the highest registry match counts were observed at –7° for ***P*-1** and +7° for ***M*-1** with counts of 12 matches (Fig. S19a and b). The exact registry match count distribution for values ≤ 10 may vary depending on the specific random walk and the sampled positions. The global maxima at ±7° with 12 matches were consistently reproduced across multiple optimizations each with a sample size of 100000 events.

For the armchair orientation, the highest registry counts were found at +11° for ***P*-1** and –11° for ***M*-1** with counts of 21 matches (Fig. S19c and d). These maxima stand out clearly, with a notable gap to the second-highest values, indicating a strong preferential orientation. However, as with the zigzag orientation, variations in the random walk and sampling density may influence the detailed distribution. The global maxima at ±11° were also consistently reproduced across multiple simulations, each based on a sample size of 100000 events.

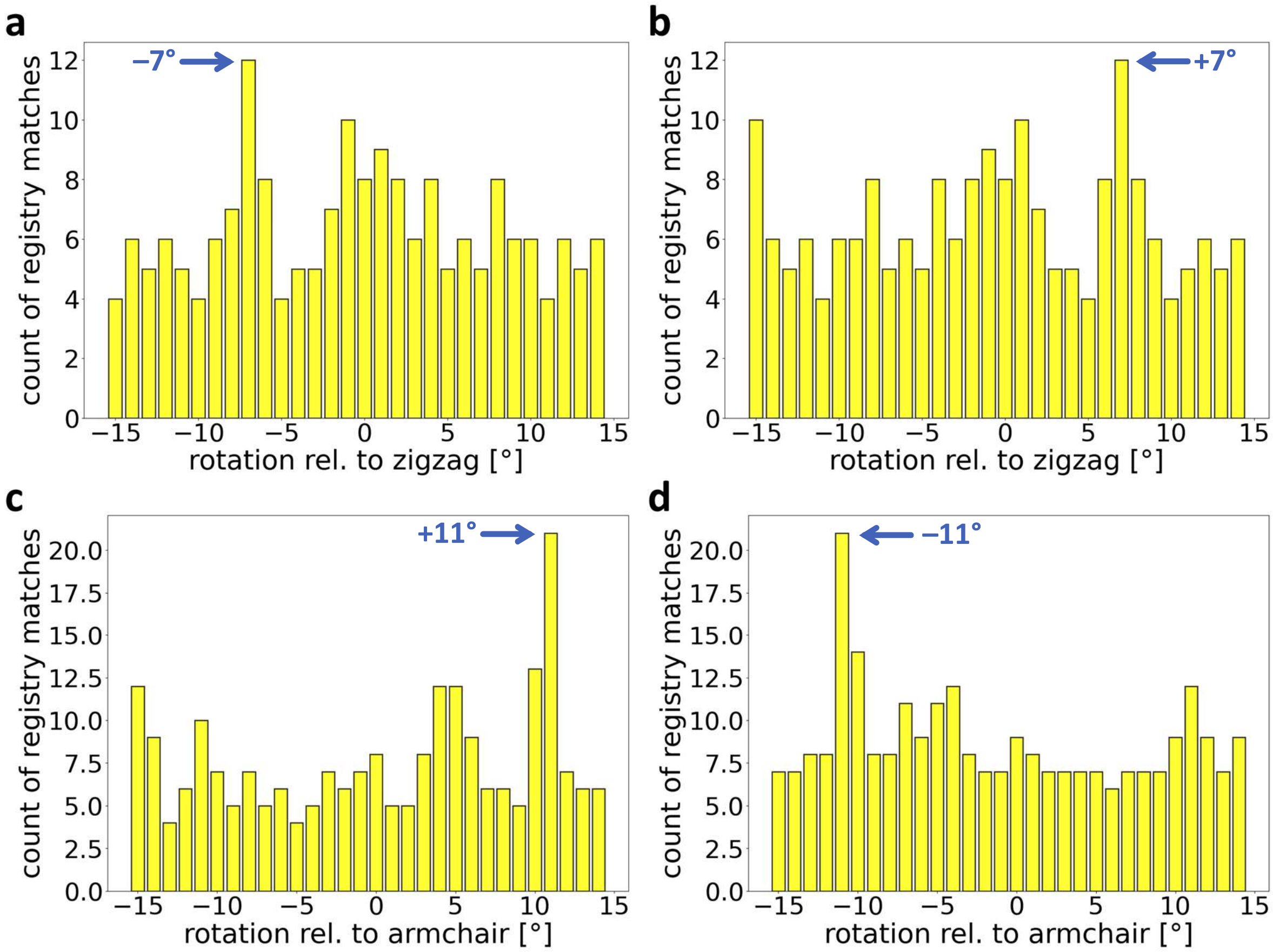

Figure S19 Bar diagrams of the maximum registry match count at different rotational steps of (a),(c) ***P*-1** and (b),(d) ***M*-1** throughout a simulation (100000 events) relative to the (a),(b) zigzag and (c),(d) armchair directions.

We repeated such an analysis for isomers of **2**. Experimentally, **2** only exhibits rotation relative to the zigzag axis. Therefore, global maxima were sampled between –30 to +30° here (cf. Fig. S20). A rotation of –7° with the highest registry match count was found for ***P*-2**/zigzag with counts of 19 matches

(cf. Fig. S20a). In a similar distribution, we found a maximum at +7° for ***M*-2**/zigzag (cf. Fig. S20b). Again, both maxima at ±7° were consistently reproduced across multiple simulations, each proceeded with a sample size of 100000 events.

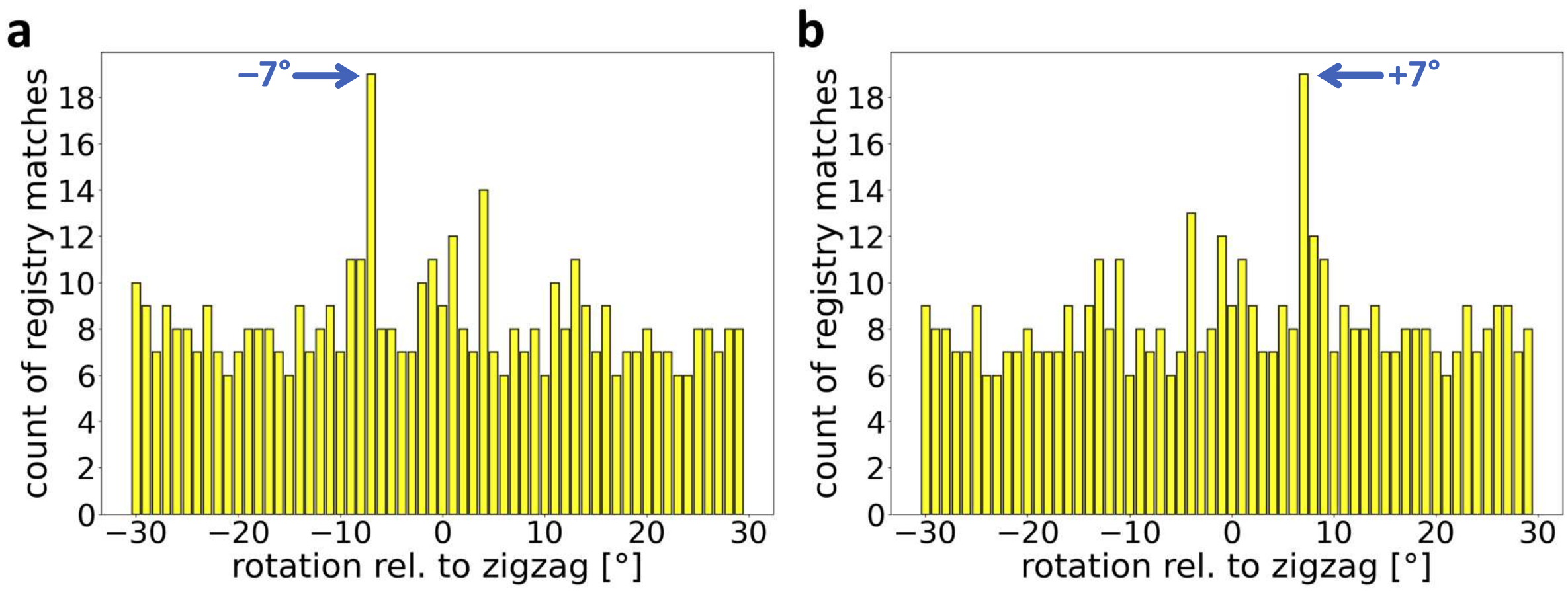

Figure S20 Bar diagrams of the maximum registry match count at different rotational steps of (a) ***P*-2** and (b) ***M*-2** throughout a simulation relative to zigzag.

5 Additional STM images and (supra-) molecular models

5.1 Detailed STM discussion of 1

Image and packing parameters supporting the STM analysis of **1** discussed in Fig. 3 of the Main Text are given in Tables S2 and S3.

Table S2 Image parameters supporting STM analysis discussed in Fig. 3 of the Main Text.

Polymorph	Subfigure	Size [nm × nm]	V_S [V]	I_t [pA]	Concentration
A	(a)	15.0 × 15.0	−1.85	30	saturated
B	(b)	15.0 × 15.0	−1.60	6	saturated, diluted ×2
A′	(c)	15.0 × 15.0	−1.80	6	saturated, diluted ×20
B′	(d)	15.0 × 15.0	−1.70	6	saturated

Table S3 Packing parameters supporting STM analysis discussed in Fig. 3 of the Main Text.

Polymorph	Enantiomer	*a* [nm]	*b* [nm]	$\gamma(a,b)$ [°]	$\gamma(c,d)$ [°]	$\gamma(c,d')$ [°]
A	***P*-1**	4.7 ± 0.2	3.7 ± 0.2	60 ± 1	−7 ± 2	-
B	***P*-1**	3.7 ± 0.2	3.7 ± 0.2	60 ± 1	-	+11 ± 2
A′	***M*-1**	4.7 ± 0.2	3.7 ± 0.2	60 ± 1	+7 ± 2	-
B′	***M*-1**	3.7 ± 0.2	3.7 ± 0.2	60 ± 1	-	−11 ± 2

In addition to the analysis given in the STM discussion in the Main Text, we embedded further information regarding polymorphisms, packing defects and crystallographic parameters in Fig. S21 using overview STM images of the respective cutouts displayed in Fig. 3 of the Main Text.

Self-assembled monolayers (SAMs) of **1** at the solution/solid interface of the respective compounds in 1,2,4-trichlorobenzene (TCB) and highly oriented pyrolytic graphite (HOPG) are shown in Fig. S21. **1** forms a densely covered SAM with three distinguishable 2D crystalline polymorphs (A, B, and C). For polymorph A, a unit cell of *a* = (4.7 ± 0.2) nm, *b* = (3.7 ± 0.2) nm and $\gamma(a,b)$ = (60 ± 1)° with one molecule per unit cell is indexed (red in Fig. S21a (top) and g), and an alignment of unit cell vector *b* along HOPG zigzag direction direction *d* is found. The backbones pack densely along lattice vector *b*, and point towards each other along lattice vector *a* which leads to the formation of intramolecular nanopores most probably filled by TCB molecules, which however remain invisible here (and are thus omitted in the models). For polymorph C, a unit cell of *a* = (11.7 ± 0.3) nm, *b* = (3.7 ± 0.2) nm and $\gamma(a,b)$ = (60 ± 1)° with three molecules per unit cell is indexed (red in Fig. S21a (bottom) and k), and an alignment of

unit cell vector *b* along HOPG zigzag direction *d* is found. Along unit cell vector *a*, long packing distances (alike in polymorph A, e_2 = (4.7 ± 0.2) nm), and shorter distances (e_1 = (3.7 ± 0.2) nm) are observed (cf. Fig S21k). In this denser packing, additional bright image features are found (cf. arrow 1 in Fig. S21a), which are attributed to geometrically constrained TCB solvent molecules (cf. Fig. S21k). For the majority of the molecules in polymorph C, a similar image contrast as in polymorph A is observed. However, in the central row of the densely packed region (arrow 2 in Figure S21a), six bright image features in a C_6-symmetric alignment in the positions of the spokes of **1** are dominant, and will be discussed below. In both polymorphs (A and C), the unit cell vector *b* is aligned along one of the three HOPG zigzag directions, *d*, and each molecule of **1** is rotated so that its north-south axis is oriented along *c* with $\gamma(c,d)$ = –(7 ± 2)°. For polymorph B, a unit cell of *a* = *b* = (3.7 ± 0.2) nm, $\gamma(a,b)$ = (60 ± 1)° with one molecule per unit cell is observed. The molecules, including TCB, pack in similar fashion as the dense packing observed in polymorph C, however with a rotation of the unit cell vector *b* relative to the armchair direction *d*' with $\gamma(b,d')$ = (14 ± 1)°. Each of the six bright features in the circle in Figure S21b corresponds to tilted peripheric phenyl substituents of the encircled molecule of **1**, and the six corresponding units of the six neighbors in a densely packed arrangement, so that the overall image contrast resembles a sixfold paddle wheel. For each of the polymorphs, A, B, and C, shown in Figure S21 (left panel), the respective enantiomer, A’, B’, and C’, is observed and shown in Figure S21 (right panel).

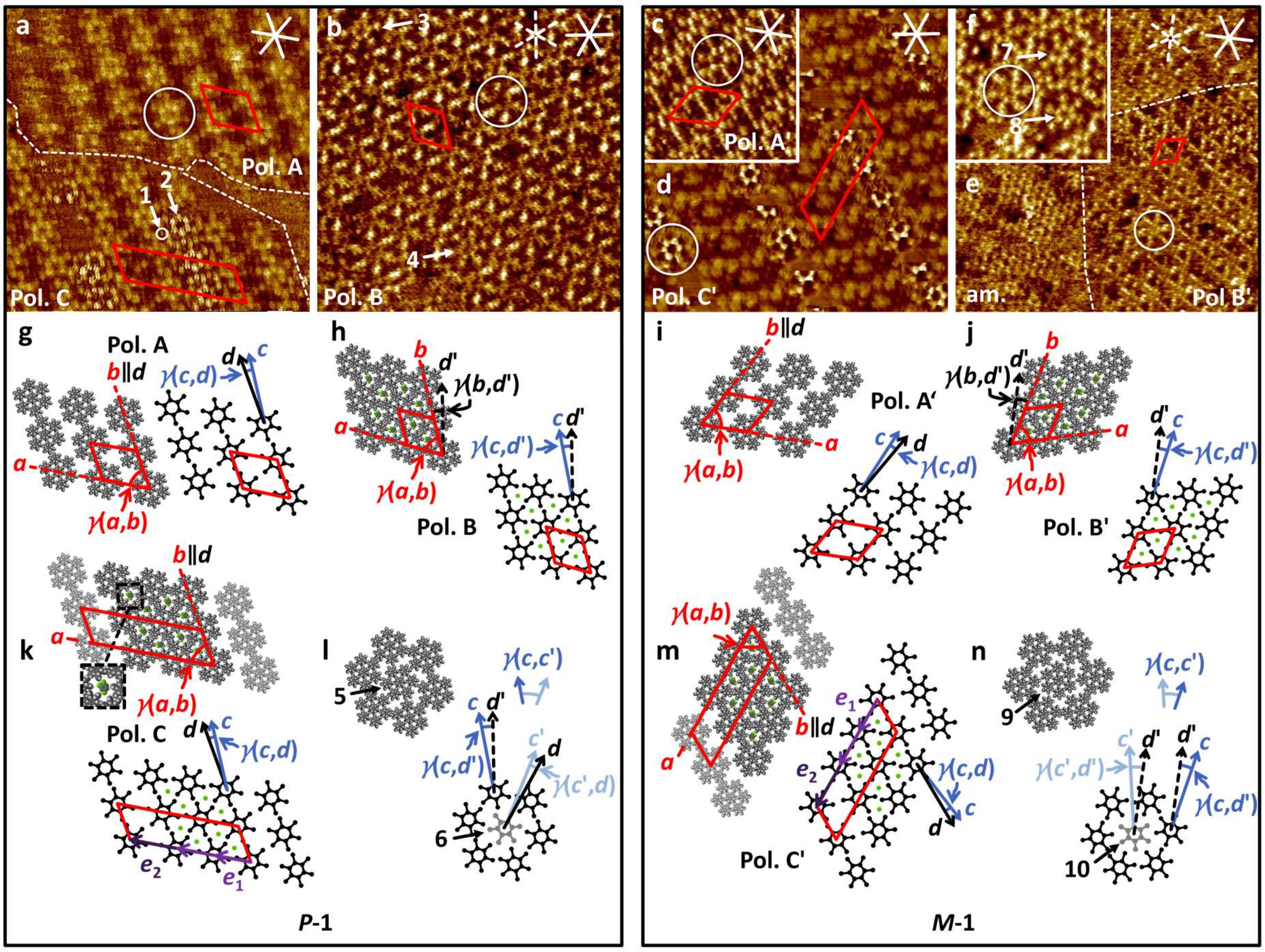

Figure S21 (a)–(f) STM images, (g)–(n) (supra-) molecular models and schematic models of supramolecular nanopatterns of **1** at the solution/HOPG interface using TCB as solvent. (a), (g), (k) Polymorph A and C of ***P*-1** (clockwise backbone rotation relative to the HOPG zigzag direction; image parameters 30.0 × 30.0 nm^2, V_S = –1.85 V, I_t = 30 pA, saturated solution; unit cell and additional packing parameters of polymorph (pol.) A: a = (4.7 ± 0.2) nm, b = (3.7 ± 0.2) nm, $\gamma(a,b)$ = (60 ± 1)°, $b \| d$, $\gamma(c,d)$ = –(7 ± 2)°, and of polymorph C: a = (11.7 ± 0.3) nm, b = (3.7 ± 0.2) nm, $\gamma(a,b)$ = (60 ± 1)°, $b \| d$, $\gamma(c,d)$ = –(7 ± 2)°, e_1 = (3.7 ± 0.2) nm, e_2 = (4.7 ± 0.2) nm); (b), (h), (l) polymorph B of ***P*-1** (counterclockwise backbone rotation relative to the HOPG armchair direction; image parameters 30.0 × 30.0 nm^2, V_S = –1.60 V, I_t = 6 pA, saturated solution diluted by × 2; unit cell and additional packing parameters of polymorph B: $a = b$ = (3.7 ± 0.2) nm, $\gamma(a,b)$ = (60 ± 1)°, $\gamma(b,d')$ = (14 ± 1)°, $\gamma(c,d')$ = +(11 ± 2)°; arrows 3, 4, 5, and 6 indicate individual molecules of ***M*-1** oriented along d with $\gamma(c',d)$ = +(11 ± 2)° on a lattice site surrounded by six molecules of ***P*-1**); (c), (d), (i), (m) polymorph A′ and C′ of ***M*-1** (counterclockwise backbone rotation relative to the HOPG zigzag direction; image parameters, c: 15.0 × 15.0 nm^2, V_S = –1.80 V, I_t = 6 pA, saturated solution diluted by × 20; d: 30.0 × 30.0 nm^2, V_S = –0.87 V, I_t = 31 pA, saturated solution; unit cell and additional packing parameters of polymorph A′: a = (4.7 ± 0.2) nm, b = (3.7 ± 0.2) nm, $\gamma(a,b)$ = (60 ± 1)°, $b \| d$, $\gamma(c,d)$ = +(7 ± 2)°, and of polymorph C′: a = (11.7 ± 0.3) nm, b = (3.7 ± 0.2) nm, $\gamma(a,b)$ = (60 ± 1)°, $b \| d$, $\gamma(c,d)$ = +(7 ± 2)°, e_1 = (3.7 ± 0.2) nm, e_2 = (4.7 ± 0.2) nm); (e), (f), (j), (n) polymorph B′ of ***M*-1** (clockwise backbone rotation relative to the HOPG armchair direction; image parameters, e: 45.0 × 45.0 nm^2, V_S = –1.70 V, I_t = 6 pA, saturated solution diluted by × 2; f: 15.0 × 15.0 nm^2, V_S = –1.80 V, I_t = 6 pA, saturated solution diluted by × 2; unit cell and additional packing parameters of polymorph B′: $a = b$ = (3.7 ± 0.2) nm, $\gamma(a,b)$ = (60 ± 1)°, $\gamma(b,d')$ = (14 ± 1)°, $\gamma(c,d')$ = –(11 ± 2)°; arrows 7, 8, 9, and 10 indicate individual molecules of ***P*-1** oriented along d' with $\gamma(c',d')$ = +(11 ± 2)° on a lattice site surrounded by six molecules of ***M*-1**), The red, blue, as well as solid and dashed white (black) lines or arrows indicate unit cells (a, b), backbone orientations (c, c'), as well as HOPG zigzag (d) and armchair (d') directions, respectively. The violet arrows indicate the positions of molecules along unit cell vector b relative to the origin (with distances e_1, e_2). All samples were thermally annealed to 80 °C for 20 s prior to STM imaging as a routine procedure in order to promote 2D self-assembly

5.2 Additional STM images of 1

Figure S22 shows additional detail STM images of **1** at the solution/HOPG interface using TCB as solvent. We found further surface regions covered by the discussed polymorphs shown in Fig. 3 of the Main Text and Figure S21. We observed regions populated by ***P*-1** forming (a) polymorph A (with amorphous areas), (c) polymorph B or (e) polymorph C and regions covered by ***M*-1** forming (b) polymorph A′, (d) polymorph B′ or (f) polymorph C′. The observed rotations relative to zigzag are $\gamma(c,d) = -(7 \pm 2)°$ for ***P*-1** found in (a) and (e) and $\gamma(c,d) = +(7 \pm 2)°$ for ***M*-1** found in (b) and (f). Relative to armchair, the orientations are $\gamma(c,d') = +(11 \pm 2)°$ for ***P*-1** found in (c) and $\gamma(c,d') = -(11 \pm 2)°$ for ***M*-1** found in (d).

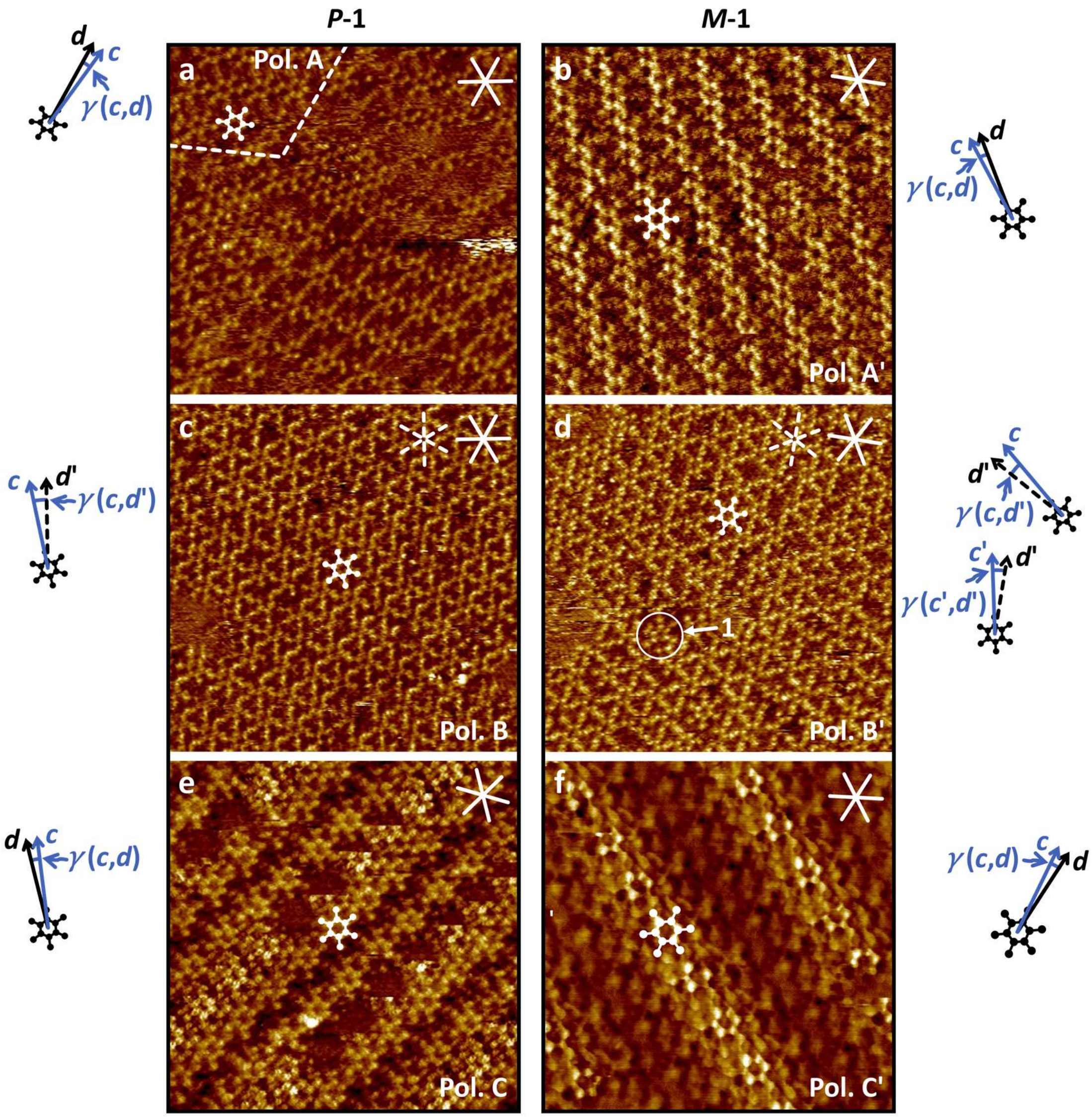

Figure S22 Additional STM images of **1** at the solution/HOPG interface using TCB as solvent. (a), (c), (e) Polymorphs A, B and C of ***P*-1**; image parameters: (a) 35.0 × 35.0 nm², $V_S = -1.6$ V, $I_t = 6$ pA, saturated solution diluted by × 2; (c) 38.0 × 38.0 nm², $V_S = -1.8$ V, $I_t = 6$ pA, saturated solution diluted by × 2; (e) 30.0 × 30.0 nm², $V_S = -1.8$ V, $I_t = 4$ pA, saturated solution diluted by × 10. (b), (d), (f) Polymorphs A′, B′ and C′ of ***M*-1**; image parameters: (b) 30.0 × 30.0 nm², $V_S = -1.8$ V, $I_t = 6$ pA, saturated solution diluted by × 10; (d) 38.0 × 38.0 nm², $V_S = -1.8$ V, $I_t = 6$ pA, saturated solution diluted by × 2; (f) 23.0 × 23.0 nm², $V_S = -1.17$ V, $I_t = 101$ pA, saturated solution. Grey dots and lines represent a schematic model of a single respective isomer of **1** placed over one molecule observed in the respective STM image. Blue and white (bold or dashed) lines represent the backbone direction and zigzag or armchair axes, respectively.

As discussed earlier, packing defects may occur for polymorphs B and B' in which one molecule occupies a lattice site that is surrounded by six molecules with a different orientation/isomerism on the substrate. Another occurrence may be observed in Fig. S22d where a ***P*-1** isomer (cf. arrow 1 in (d)) occupies a lattice site with a rotation of $\gamma(c',d')$ = +(11 ± 2)° relative to the armchair axis. Besides packing defects, we observed surface regions at domain boundaries either covered by the same isomer but with different orientations or covered by different isomers. Fig. S23 shows such regions in which single molecules are analyzed regarding their orientation relative to the substrate axes. White-dashed lines confine molecules with identical orientation. In (a), we observed a surface area covered by the ***P*-1** isomer either oriented with $\gamma(c',d')$ = +(11 ± 2)° relative to the armchair axis within a region covered by pol. B or oriented with $\gamma(c,d)$ = –(7 ± 2)° relative to the zigzag axis in a region covered by pol. A. In (b), we observed a surface area either covered by the ***P*-1** isomer (region of pol. A) or the ***M*-1** isomer (region of pol. B'). Thereby, the ***P*-1** isomer is oriented with $\gamma(c,d)$ = –(7 ± 2)° relative to the zigzag axis and the ***M*-1** isomer is oriented with $\gamma(c',d')$ = –(11 ± 2)° relative to the armchair axis.

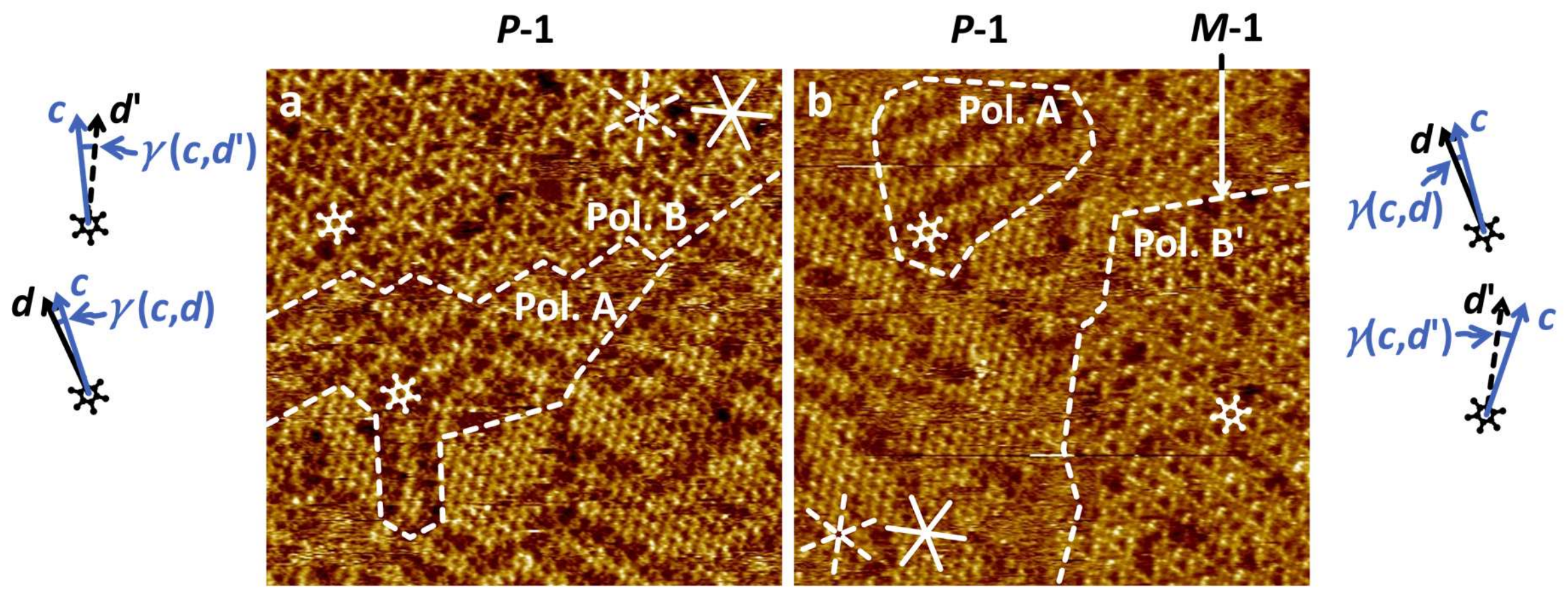

Figure S23 Additional STM images of **1** at the solution/HOPG interface using TCB as solvent. (a) Region covered by ***P*-1**; image parameters: 50.0 × 50.0 nm^2, V_S = –1.7 V, I_t = 6 pA, saturated solution diluted by × 2; (b) Region covered by ***P*-1** and ***M*-1**; image parameters: 50.0 × 50.0 nm^2, V_S = –1.7 V, I_t = 6 pA, saturated solution diluted by × 2. Grey dots and lines represent a schematic model of a single respective isomer of **1** placed over one molecule observed in the respective STM image. Blue and white (bold or dashed) lines represent the backbone direction and zigzag or armchair axes, respectively.

5.3 Packing chirality of 1

We observed chirality at different levels. At the molecular level, individual atropisomers (*P* and *M*) of **1** orient differently relative to *e.g*. the zigzag axis, *d*, to form chiral adsorbate structures (cf. Fig. S24a). At the supramolecular level, ordering of atropisomers leads to homochiral domains containing the discussed Polymorphs A, B, C or their mirror images A', B' or C'. However, each polymorph exhibits an additional axial chirality. Taking pol. A as an example, molecules interact along unit cell vector *b* in parallel with *d*. The parallelism is caused by a synchronous backbone orientation while interaction of the molecules follows the zigzag direction (Fig. S24b, left). However, an offset is possible if the interaction propagates towards a direction non-identical to zigzag while the backbone orientation is identical (cf. Fig. S24b, right). Both of these arrangements in theory exhibit a mirror image as well (cf. Fig. S24c).

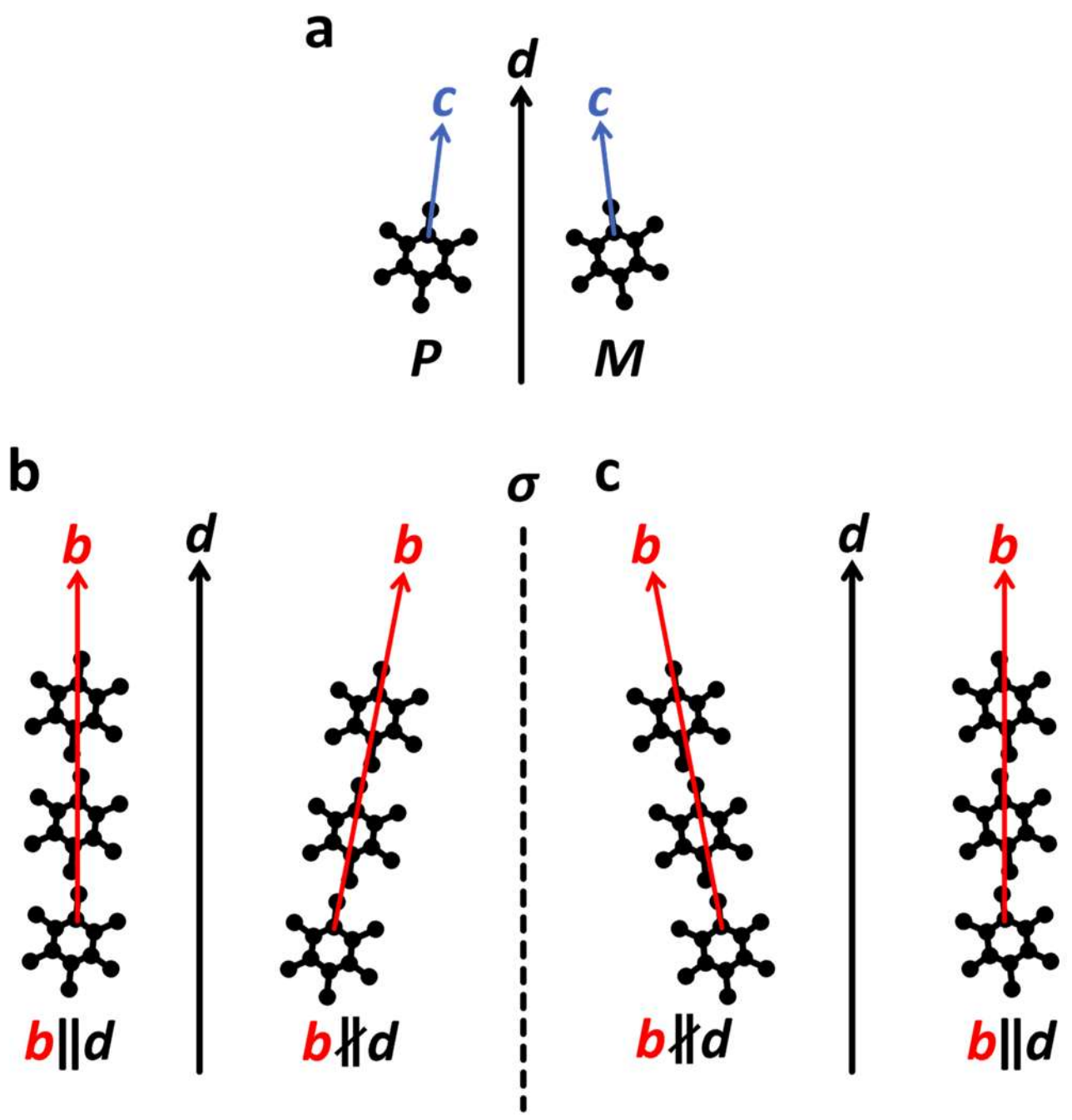

Figure S24 Schematic analysis of chirality at different levels. (a) Chirality of single molecules relative to the zigzag axis *d* and (b),(c) packing chirality considering chiral interaction motifs either following the zigzag direction *d* or propagating towards a direction ≠ *d*.

We observed this type of axial chirality within the supramolecular nanopatterns of ***P*-1**, more precisely in small domains of Pol. A. Regions 1 and 2 marked with white rectangles in the STM image in Fig. S25a show the two possible arrangements discussed in Fig. S24b. STM image cutouts and schematic models in Fig. S25b and c focus on three molecules which either interact with a direction in parallel to the zigzag axis (cf. Fig. S25b) or with a direction not in parallel to zigzag (cf. Fig. S25c).

We assume that for every enantiomorphic polymorph pair (A/A', C/C'), two such enantiomorphic axial chiral arrangements should be observable where the unit cell vector *b* is not parallel to the zigzag direction. The considerations should apply to Polymorphs B/B' as well but the chirality is not easily detected due to the different image contrast.

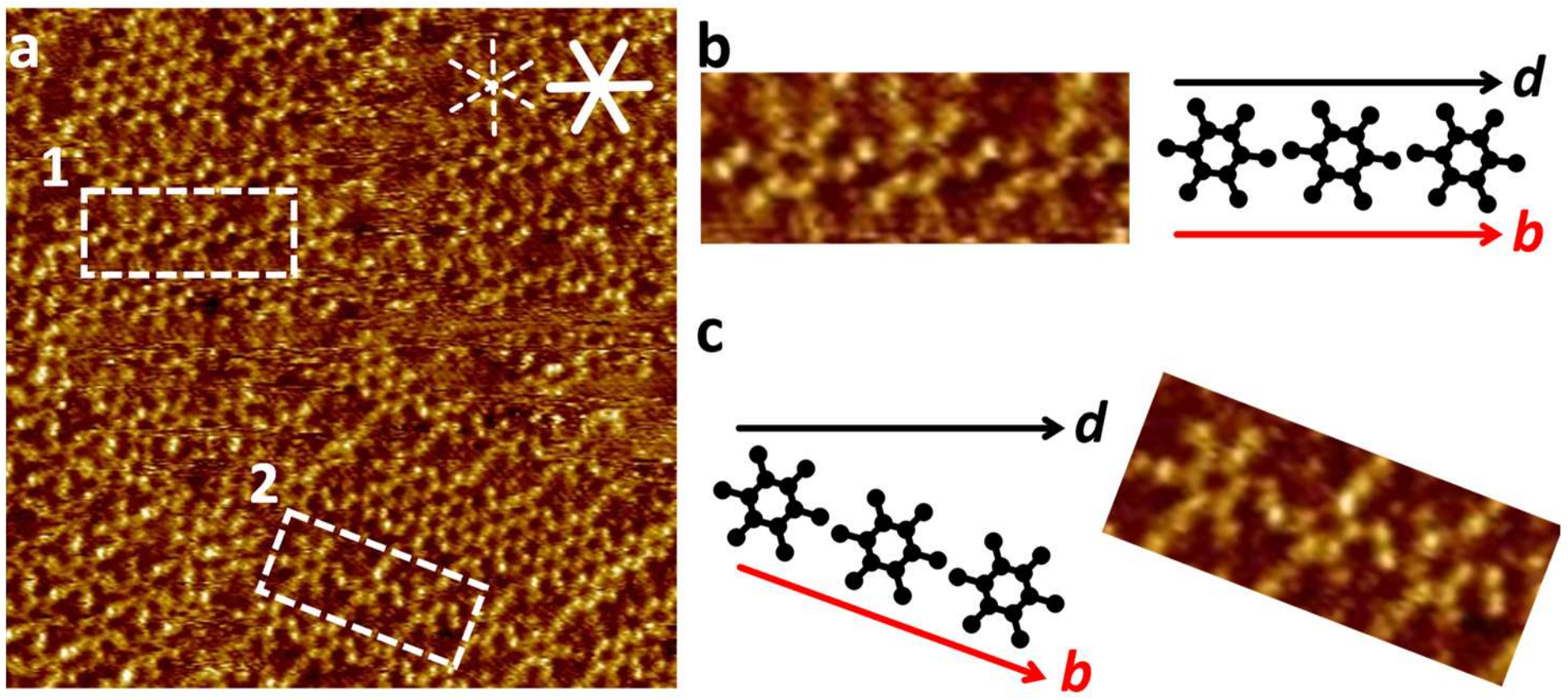

Figure S25 (a) STM image and (b), (c) image cutouts and schematic models of **1** at the solution/HOPG interface using TCB as solvent. (a) image parameters: 40.0 × 40.0 nm², V_S = –1.6 V, I_t = 6 pA, saturated solution diluted by × 2; (b) Region (1 in (a)) covered by three interacting molecules of ***P*-1** which interact in parallel to the zigzag direction: image cutout: 12.3 × 5.0 nm², black dots and lines represent a schematic model of three interacting molecules; (c) Region (2 in (a)) covered by three interacting molecules of ***P*-1** which do not interact in parallel to the zigzag direction: image cutout: 12.3 × 5.0 nm², black dots and lines represent a schematic model of three interacting molecules.

5.4 STM discussion of 2

2 forms a densely covered SAM with domain sizes of identically oriented molecules of > 100^2 nm^2 (see overview STM image in Fig. S28). Throughout all measurements, one 2D crystalline pattern was observed to which a unit cell of *a* = *b* = (4.2 ± 0.2) nm and $\gamma(a,b)$ = (59 ± 2)° with one molecule per unit cell is indexed (red in Fig. S26a and c), and an alignment of unit cell vector *b* relative to the HOPG zigzag direction *d* with $\gamma(b,d)$ = (41 ± 2)° is found. Each of the repeating units appears as a set of six bright features (arrow 1 in Figure S26a) in a C_6 symmetric alignment around a dark center (arrow 2), and with a dark periphery (arrow 3) in which medium-dark hexagons are found. These are, in that order, attributed to the six spoke units, the central phenylene hub, and the MSW rim and corner units. The backbones pack densely so that each of the dimesitylphenyl units of each molecule is in contact with two such units of adjacent molecules. Each molecule of **2** is rotated so that its north-south axis is oriented along *c* with $\gamma(c,d)$ = –(7 ± 2)° relative to the HOPG zigzag direction, *d*. Similar to **1**, for the packing of **2** shown in Figure S26 (left panel), the respective enantiomer with alike contrast features and $\gamma(c,d)$ = +(7 ± 2)° is observed and shown in Figure S26 (right panel). Despite numerous attempts and using different concentrations of **2** in TCB, no other polymorphs were observed.

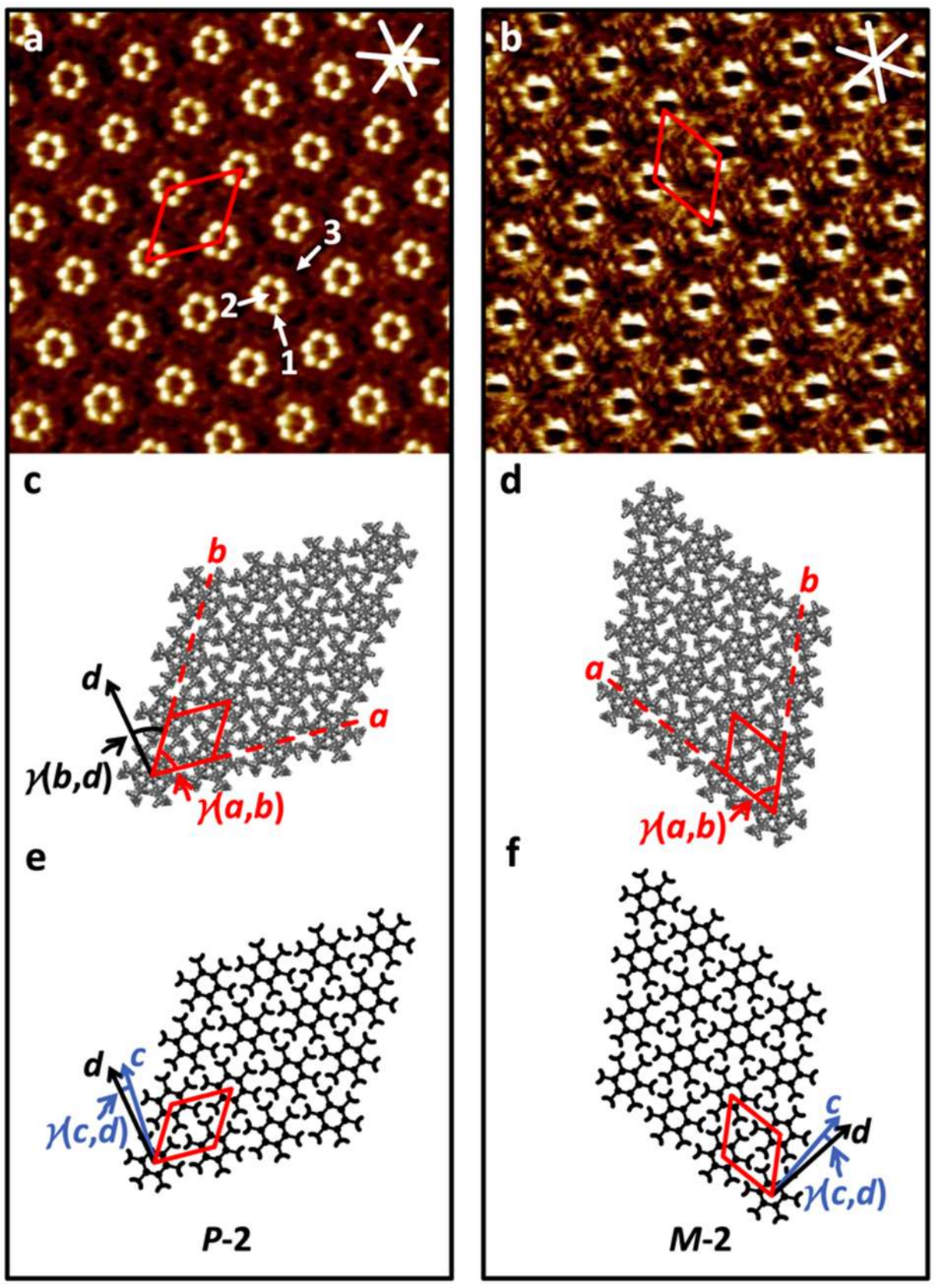

Figure S26 (a), (b) STM images, (c), (d) (supra-) molecular models and schematic models of supramolecular nanopatterns of **2** at the solution/HOPG interface using TCB as solvent, as well as (e), (f) corresponding 2D plots of optimized molecular registries. (a), (c), (e) supramolecular nanopattern of ***P*-2** (counterclockwise backbone rotation relative to the HOPG zigzag direction; (a) image parameters 25.0 × 25.0 nm^2, V_S = –1.30 V, I_t = 20 pA, *c* = 10^{-4} M; (c) unit cell and additional packing parameters: *a* = *b* = (4.2 ± 0.2) nm, γ(*a*,*b*) = (59 ± 2)°, γ(*b*,*d*) = (41 ± 2)°, γ(*c*,*d*) = –(7 ± 2)°; (b), (d), (f) supramolecular nanopattern of ***M*-2** (clockwise backbone rotation relative to the HOPG armchair direction; (b) image parameters 25.0 × 25.0 nm^2, V_S = –1.70 V, I_t = 10 pA, *c* = 10^{-4} M; (d) unit cell and additional packing parameters: *a* = *b* = (4.2 ± 0.2) nm, γ(*a*,*b*) = (59 ± 2)°, γ(*c*,*d'*) = +(7 ± 2)°. The red, blue, as well as solid white (black) lines or arrows indicate unit cell vectors *a*, *b*, nonaphenylene orientations *c*, as well as HOPG zigzag directions *d*, respectively. All samples were thermally annealed to 80 °C for 20 s prior to STM imaging as a routine procedure in order to promote 2D self-assembly.

5.5 Additional STM images of 2

The enantiomorphic nanopatterns of isomers of **2** discussed in Fig. S26 were observed in other surface regions as well (cf. Fig. S27). Both isomers populate the graphite surface only with rotation relative to zigzag not to armchair. A region populated by ***P*-2** is found in (a) with a rotation of $\gamma(c,d)$ = +(7 ± 2)°, whereas ***M*-2** is found in (b) with an orientation of $\gamma(c,d)$ = –(7 ± 2)°. Coincidentally, we found molecules of ***P*-2** and ***M*-2** (cf. arrows 1 and 2 in (a) and (c), respectively) with a darker appearing contrast than the surrounding molecules. We assume that the contrast change is a result of a displacement of the molecule's registry positions relative to the underlying substrate, similar to a contrast change due to the registry of an alkyl chain.[63]

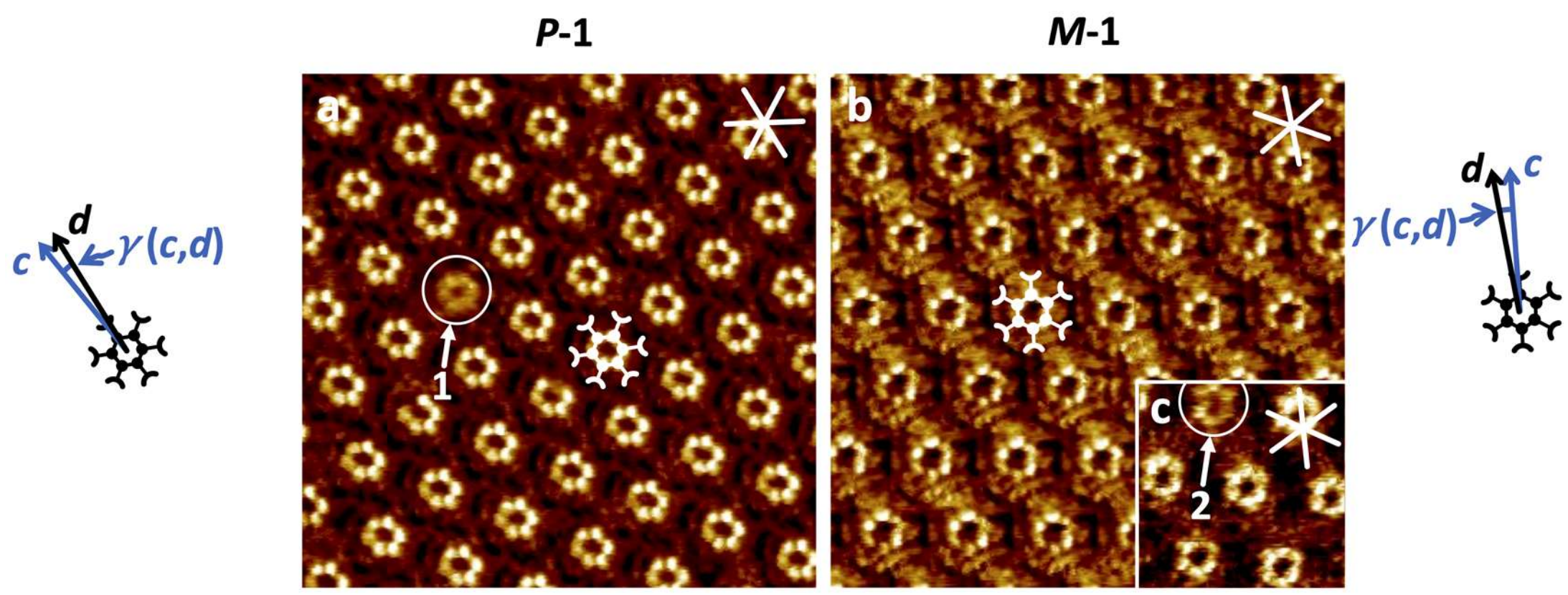

Figure S27 Additional STM images of **2** at the solution/HOPG interface using TCB as solvent. (a) Region covered by ***P*-2**; image parameters: (a) 30.0 × 30.0 nm^2, V_S = –1.05 V, I_t = 18 pA, c = 10^{-4} M; (b) Region covered by ***M*-2**; image parameters: (b) 25.0 × 25.0 nm^2, V_S = –1.7 V, I_t = 10 pA, c = 10^{-4} M; (c) Region covered by ***M*-2**; image parameters: (b) 10.0 × 10.0 nm^2, V_S = –1.7 V, I_t = 10 pA, c = 10^{-4} M. Grey dots and lines represent a schematic model of a single respective isomer of **2** placed over one molecule observed in the respective STM image. Blue and white (bold or dashed) lines represent the backbone direction and zigzag or armchair axes, respectively. White arrow 1 marks one molecule with a darker contrast.

The darker contrast is occurring frequently which we observed for a larger surface area (100^2 nm^2) covered by the packing of ***P*-2** / zigzag. As shown in Fig. S28, darker appearing molecules are clearly discernible from regular bright appearing ones in the overview STM image. Two occurrences are exemplarily marked (out of 26 in total) with arrows 1 and 2 in Fig. S28. We assume that the darker contrast is occurring frequently as well for ***M*-2** but only observed that in smaller surface areas.

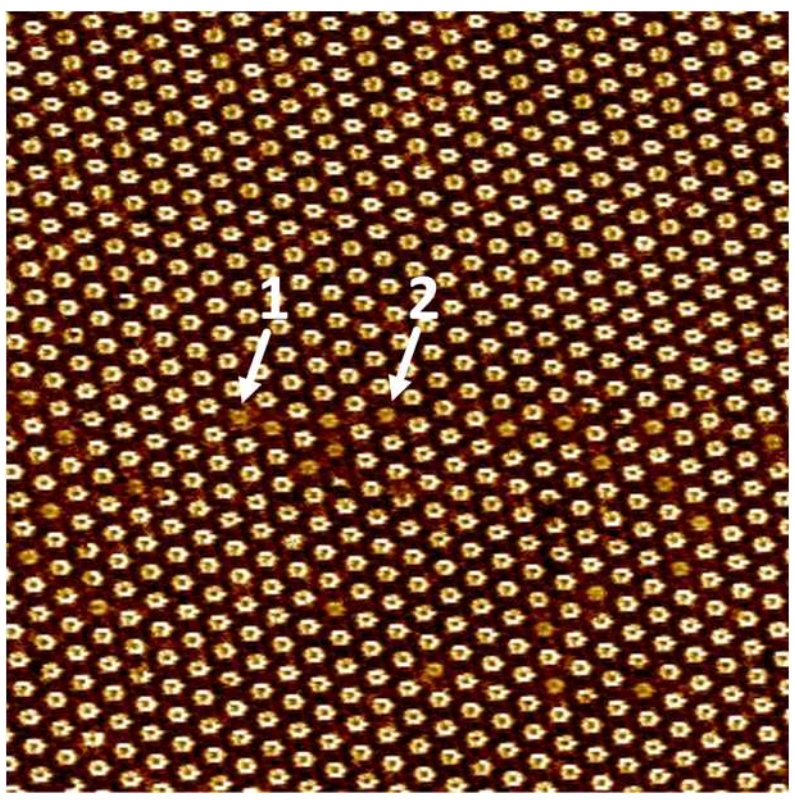

Figure S28 Overview STM image of ***P*-2** / zigzag at the solution/HOPG interface using TCB as solvent. Image parameters: (a) 100.0 × 100.0 nm^2, V_S = –0.9 V, I_t = 17 pA, c = 10^{-4} M.

5.6 STM contrast variations of 1 and 2

Regarding the molecular STM image contrast, there are general variations found depending on the supramolecular polymorphism and the orientation of the molecules relative to graphite. Throughout the performed measurements, we observed three different modes of contrast (with slight deviations, due to measurement conditions) for single molecules of *e.g.* ***P*-1**. Planar benzene rings appear dark and remain invisible in all STM images. Molecules within polymorph A (cf. Fig. 3 of the Main Text) that pack densely at two opposite corners and are oriented with −(7 ± 2)° relative to zigzag, exhibit a contrast in which the spokes appear brightest and the outer rim appears slightly darker than the spokes (cf. Fig. S29a). Molecules within polymorph C that pack densely at all corners and are oriented with −(7 ± 2)° relative to zigzag, exhibit an even greater brightness at the spokes and a slightly darker outer rim (cf. Fig. S29b). Lastly, molecules within polymorph B (cf. Fig. 3 of the Main Text) that pack densely at all corners and are oriented with +(11 ± 2)° relative to armchair, show the brightest contrast at the outer rim and lower brightness at the spokes (cf. Fig. S29c). The contrast modes are independent from the observed atropisomerism. However, the modes clearly depend on the respective polymorph and the orientation of molecules relative to the graphite axes.

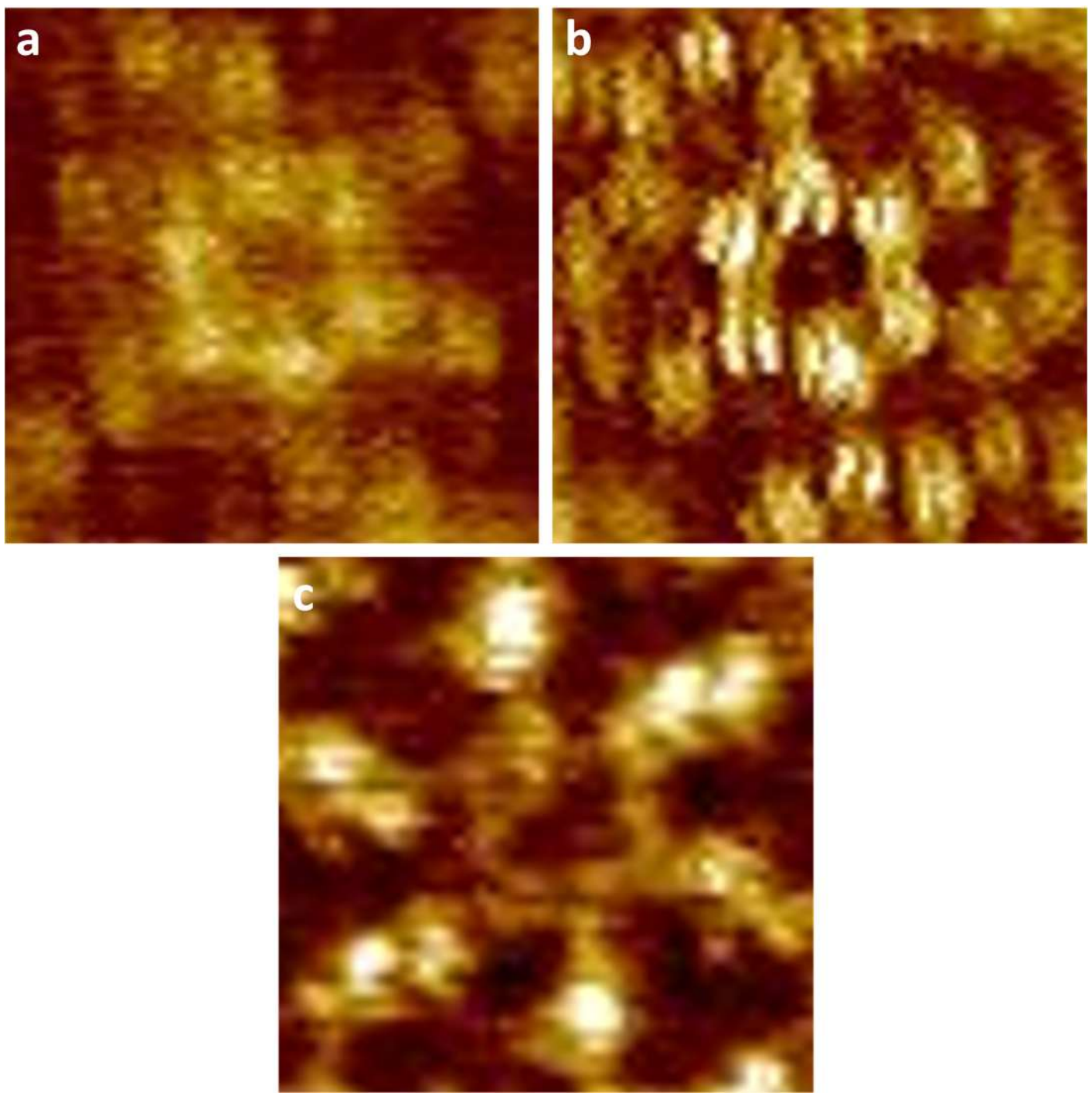

Figure S29 STM images showing only one molecule of ***P*-1** at the solution/HOPG interface using TCB as solvent. (a) One molecule of ***P*-1** within polymorph A; image parameters: 4.50 × 4.50 nm^2, V_S = −1.85 V, I_t = 30 pA, saturated solution; (b) One molecule of ***P*-1** within polymorph C; image parameters: 4.50 × 4.50 nm^2, V_S = −1.85 V, I_t = 30 pA, saturated solution; (c) One molecule of ***P*-1** within polymorph B; image parameters: 4.50 × 4.50 nm^2, V_S = −1.60 V, I_t = 6 pA, saturated solution diluted by × 2.

Regarding general contrast variations of **2**, we found one variation observed for the majority of molecules. Again, planar benzene rings remain invisible in the STM images. Taking the ***P*-2** isomer as an example, we observed a strong dark/bright difference in the contrast. At the spokes, the contrast is extremely bright and at the outer rim and dimesitylphenyl groups it is much darker (cf. Fig. S30a). At some locations, we have found molecular contrast features where the spokes appear still bright but a lot darker than usual (cf. Fig. S30b and chapter 5.5). We assume that the enantiomer occupies the lattice sites at the respective locations, as they pack densely with surrounding molecules but exhibit a non-ideal registry.

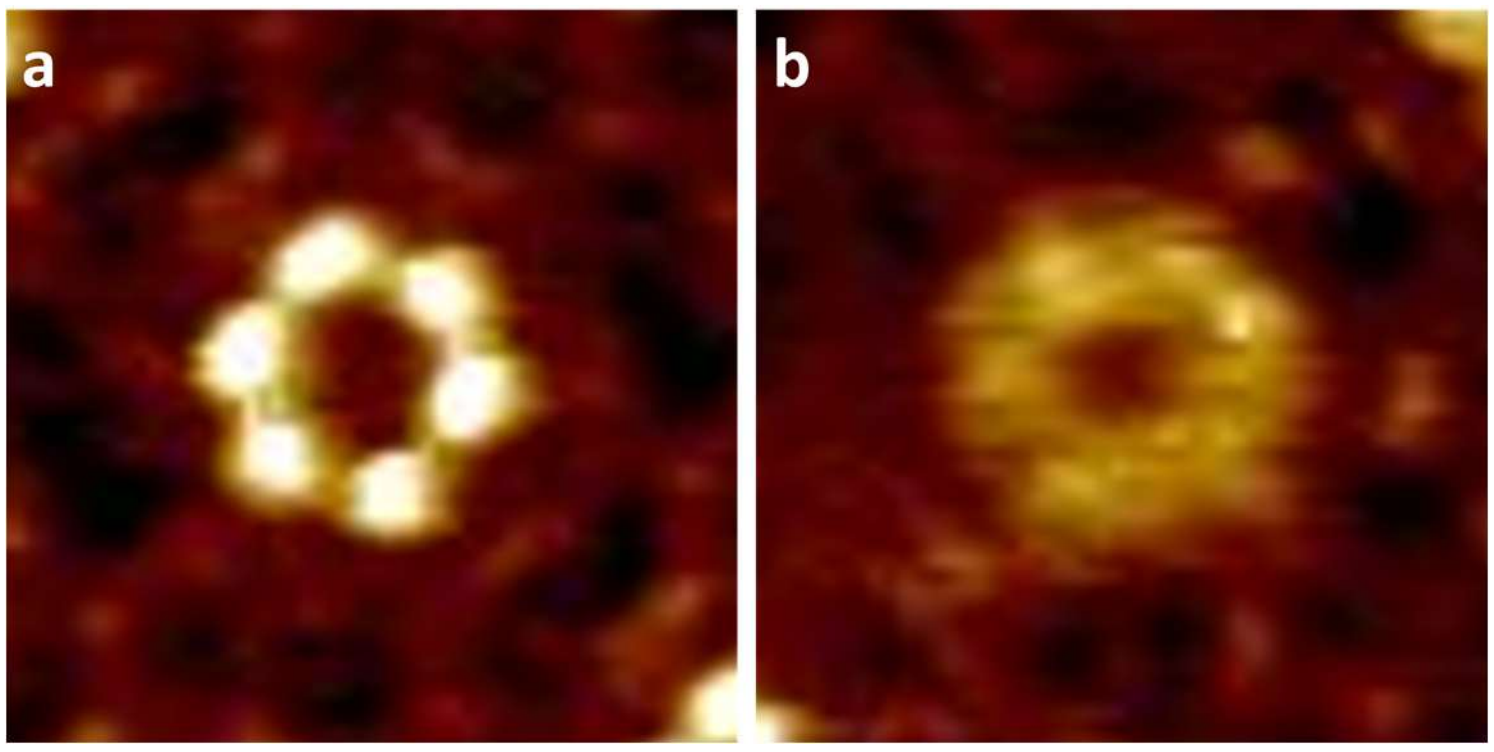

Figure S30 STM images showing only one molecule of ***P*-2** at the solution/HOPG interface using TCB as solvent. (a) One molecule of ***P*-2** with bright contrast; image parameters: 5.00 × 5.00 nm^2, V_S = –1.3 V, I_t = 20 pA, *c* = 10^{-4} M; (b) One molecule of ***P*-2** with darker contrast; image parameters: 5.00 × 5.00 nm^2, V_S = –1.05 V, I_t = 18 pA, *c* = 10^{-4} M.

We fitted one molecular model of ***P*-2** (from an adapted supramolecular model) to the STM image which is shown in Fig. S31a. The brightest contrast features originate from phenylenes contributing to the preferred registry orientation.

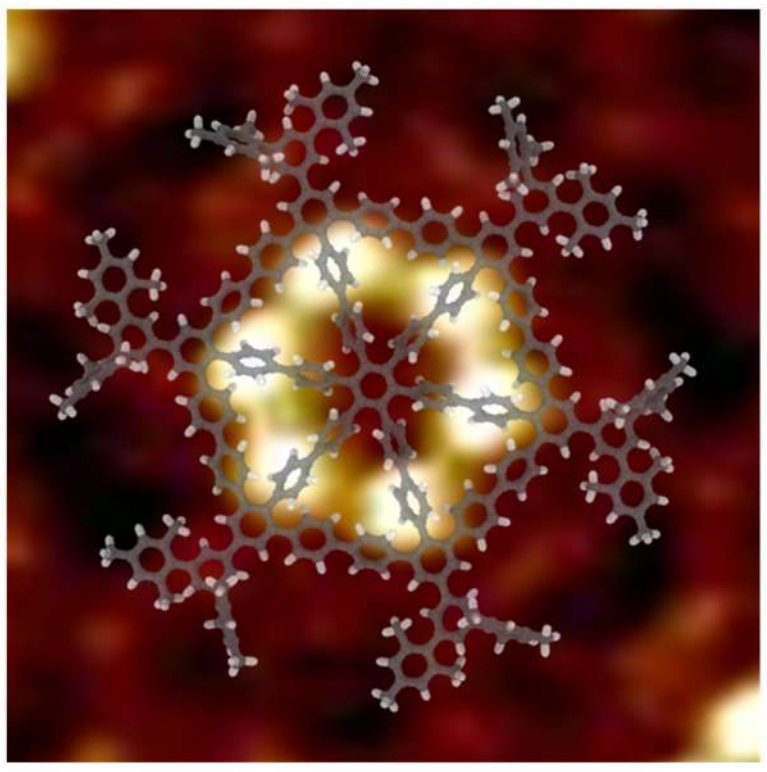

Figure S31 STM image (cf. Fig. S30a) overlayed with a molecular model of ***P*-2**.

5.7 STM discussion of 3

We investigated MSW **3** by STM which carries solubilizing dihexadecyloxyphenyl groups as well as Boc-protected amino alkyls (cf. Fig. S32).

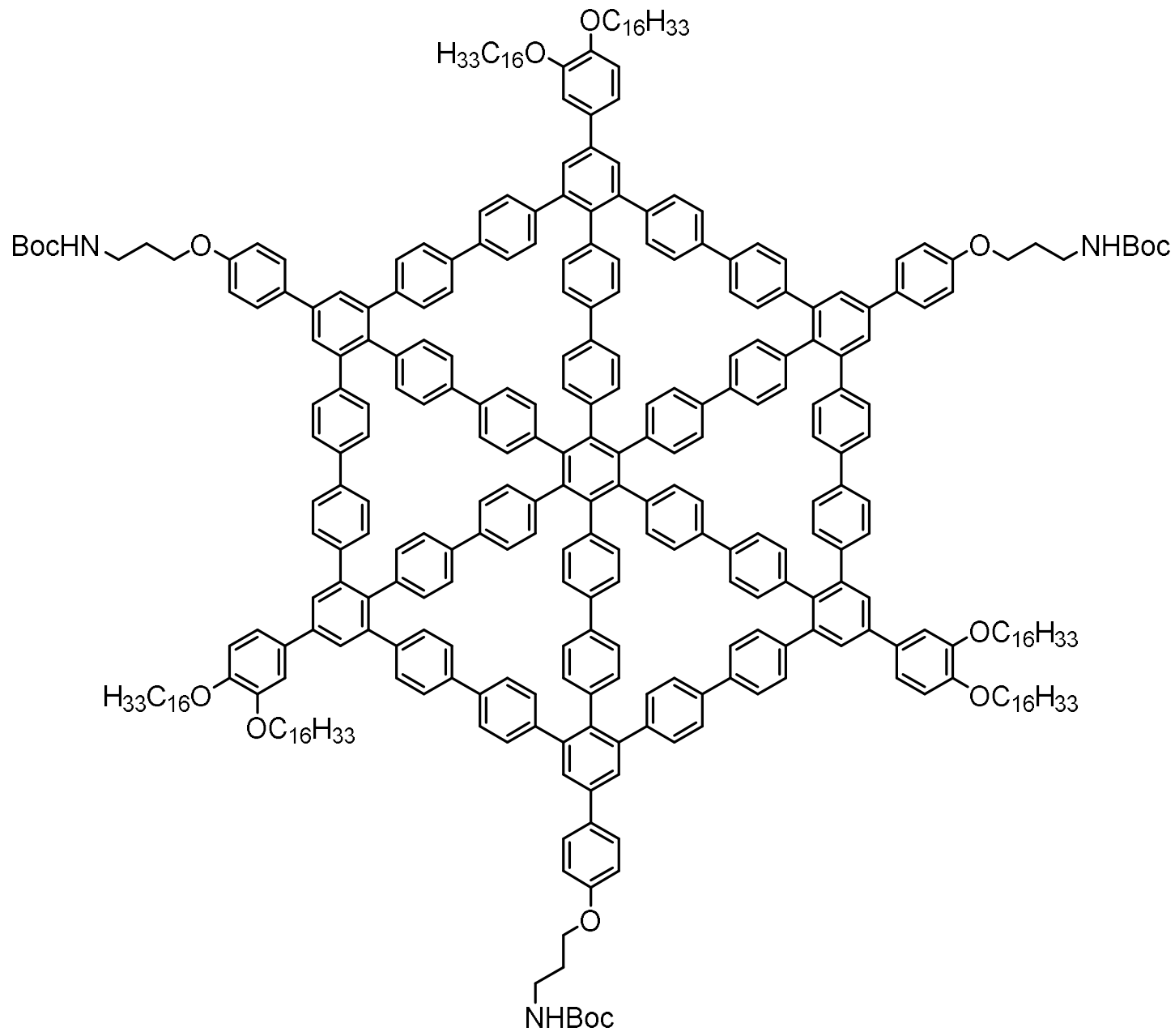

Figure S32 Chemical structure of MSW **3**.

Self-assembled monolayers of **3** at the solution/solid interface of a solution of **3** in TCB and HOPG are shown in Fig. S33. At a concentration of $c = 5 \times 10^{-6}$ M in the solution phase, **3** forms a 2D-crystalline nanopattern with a unit cell of $a = (10.5 \pm 0.3)$ nm, $b = (4.8 \pm 0.2)$ nm and $\gamma(a,b) = (81 \pm 1)°$, and an orientation of unit cell vector *a* relative to the HOPG zigzag direction d_1 is found as $\gamma(a,d_1) = (4 \pm 2)°$ (cf. Fig. S33a and b). Each MSW carries three dihexadecyloxyphenyl units. In general, the alkoxy side chains orient along two different zigzag directions d_1 and d_2. Along d_1, four alkoxy chains of two adjacent dihexadecyloxyphenyl units adsorb and pack densely in an AABB pattern. Along d_2, two alkoxy chains of two adjacent dihexadecyloxyphenyl units adsorb and pack densely in an AB pattern. Thereby, one side chain each of the two contributing dihexadecyloxyphenyl units points towards the solution phase. Along unit cell vector *b*, two MSWs pack densely with a dihexadecyloxyphenyl unit and a Boc protecting group pointing towards each other. There, both chains of the dihexadecyloxyphenyl unit presumably point towards the solution phase. Due to the bulky protecting groups, the packing is rather porous and is probably dynamically filled with TCB molecules. Regarding the molecular backbone orientation *c* relative to the HOPG zigzag direction *d* (= d_2), we found a deviation of $\gamma(c,d) = -(7 \pm 2)°$ (cf. Fig. S33c). Since **3** is based on an identical spoke and rim backbone compared to **1**, such a deviation would imply an STM observation of the ***P*-3** isomer. To test this hypothesis, we performed registry optimization of ***P*-3**.

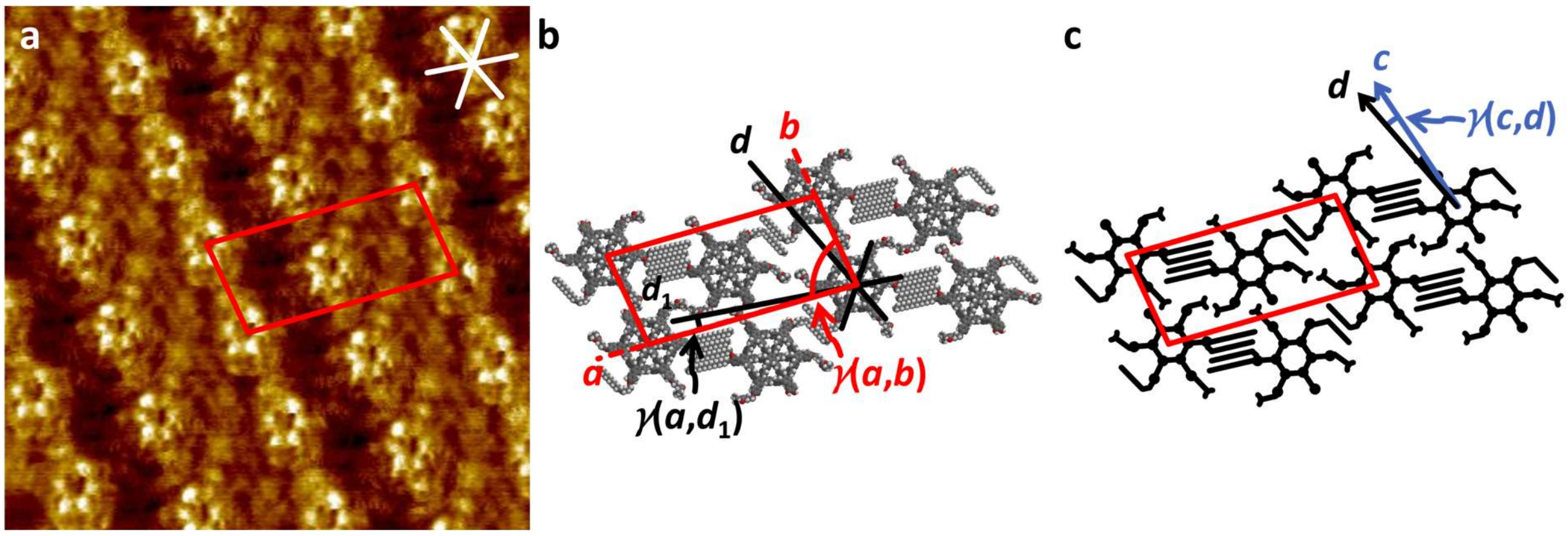

Figure S33 (a) STM image, (b) (supra-) molecular model and (c) schematic model of a supramolecular nanopattern of **3** at the solution/HOPG interface using TCB as solvent. (a) image parameters 25.0 × 25.0 nm^2, V_S = –1.0 V, I_t = 25 pA, $c = 5 \times 10^{-6}$ M; (b),(c) unit cell and additional packing parameters: a = (10.5 ± 0.3) nm, a = (4.8 ± 0.2) nm, $\gamma(a,b)$ = (81 ± 2)°, $\gamma(a,d_1)$ = (4 ± 2)°, $\gamma(c,d)$ = –(7 ± 2)°. The red, blue, as well as solid white (black) lines or arrows indicate unit cell vectors *a*, *b*, nonaphenylene orientations *c*, as well as HOPG zigzag directions *d*, respectively. All samples were thermally annealed to 80 °C for 20 s prior to STM imaging as a routine procedure in order to promote 2D self-assembly.

6 Application of the workflow

Concerning the application of the workflow, the number of available literature data for systems with sufficiently low conformational degrees of freedom containing surface-oriented hydrogen atoms is limited. The data must include high-resolution STM images as well as the orientation of the molecules relative to the graphite substrate and go beyond the trivial case of *n*-alkanes and alkane derivatives.

In the following subchapters we investigate the registry optimization of **2**, **3**, and the literature systems **MSWa** and **MSWb**.

6.1 Registry optimization of 2

The input structure of the Monte Carlo procedure of **2** was optimized at the GFN-FF level of theory, and resulted in a twist angle between the phenyl(ene) units of 56°. No changes of the twist angles were investigated. From the registry optimization, we assigned ***P*-2** and ***M*-2** for the STM images shown in the left and right panel of Figure S34, respectively. For ***P*-2**/zigzag and ***M*-2**/zigzag (cf. Figure S34a and b), we found 13 relevant hydrogen atom footprints of the phenyl(ene) units and 6 relevant hydrogen atom footprints of the methyl units, marked in yellow, within a radius of 0.2 Å around the hexagon centers, while the other 81 relevant hydrogen atoms (marked with a gradient from light to dark colors) are closer to the carbon atoms of the graphene sheet (and a histogram is shown in Figure S34b). Similar as observed for **1**, in monolayers of **2** the regions of the spokes of the MSWs appear bright, which is attributed to the close proximity of hydrogen atoms to the centers of the graphene hexagons. Although some of the hydrogen atoms of the rim are also located above the hexagon centers of the graphene, these are less tilted and lifted by the sterically demanding dimesitylphenyl units (cf. chapter 4.5). Therefore, they do not come into such close contact with the graphene and appear less bright.

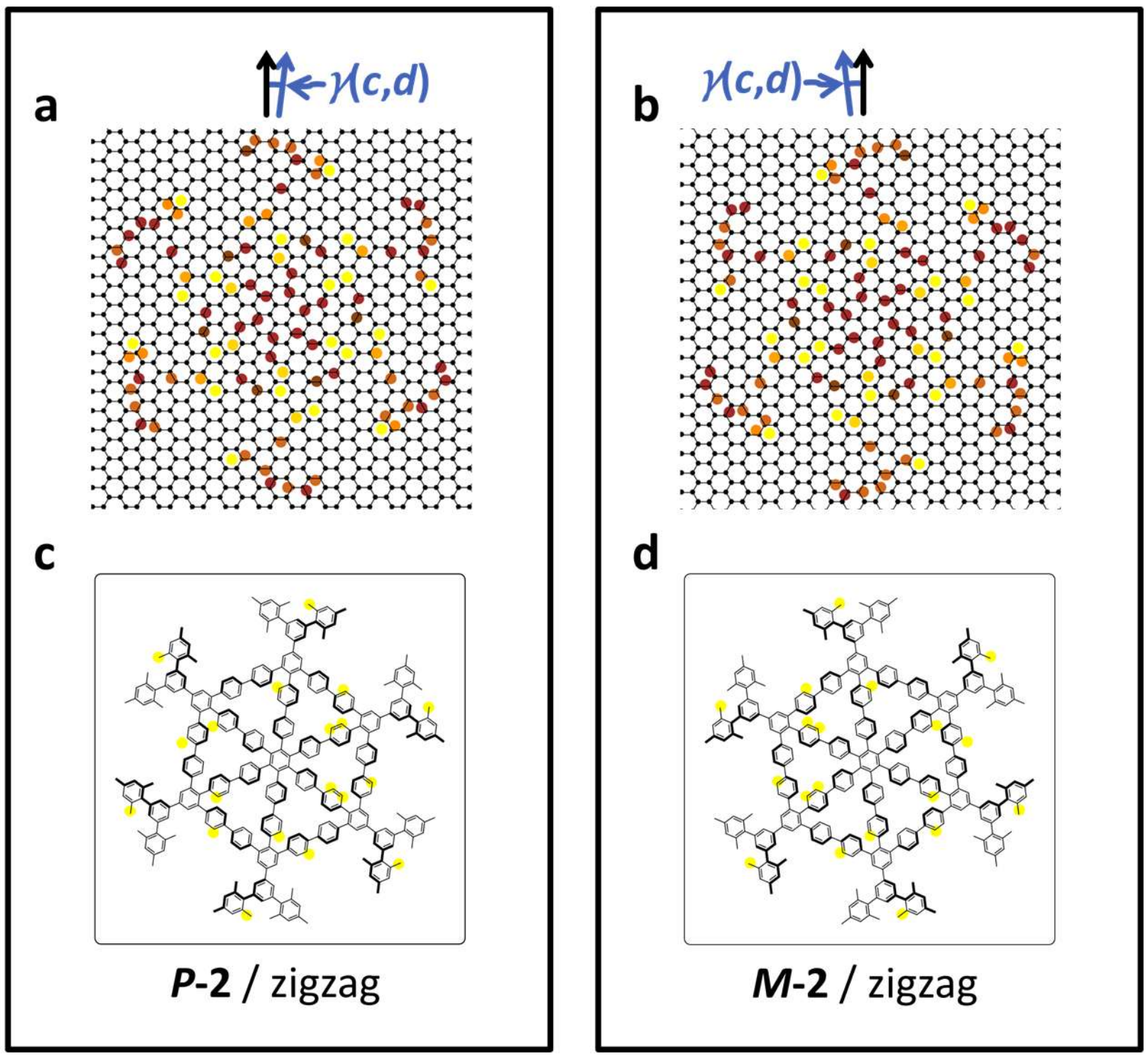

Figure S34 (a), (b) 2D plots of optimized molecular registries of (a) ***P*-2** and (b) ***M*-2** on a graphene cutout; the color gradient (light to dark) indicates increasing distances between the hydrogen atoms and the centers of the hexagons with a_0 = 2.46 Å, *i.e.* the lattice vector of graphene; all intervals are left-closed, right-open; (c), (d) chemical structures of the absolute conformations of ***P*-2** and ***M*-2**, as indexed to the domains, with registry positions highlighted in yellow (hydrogen-to-center distance ≤ 0.2 Å).

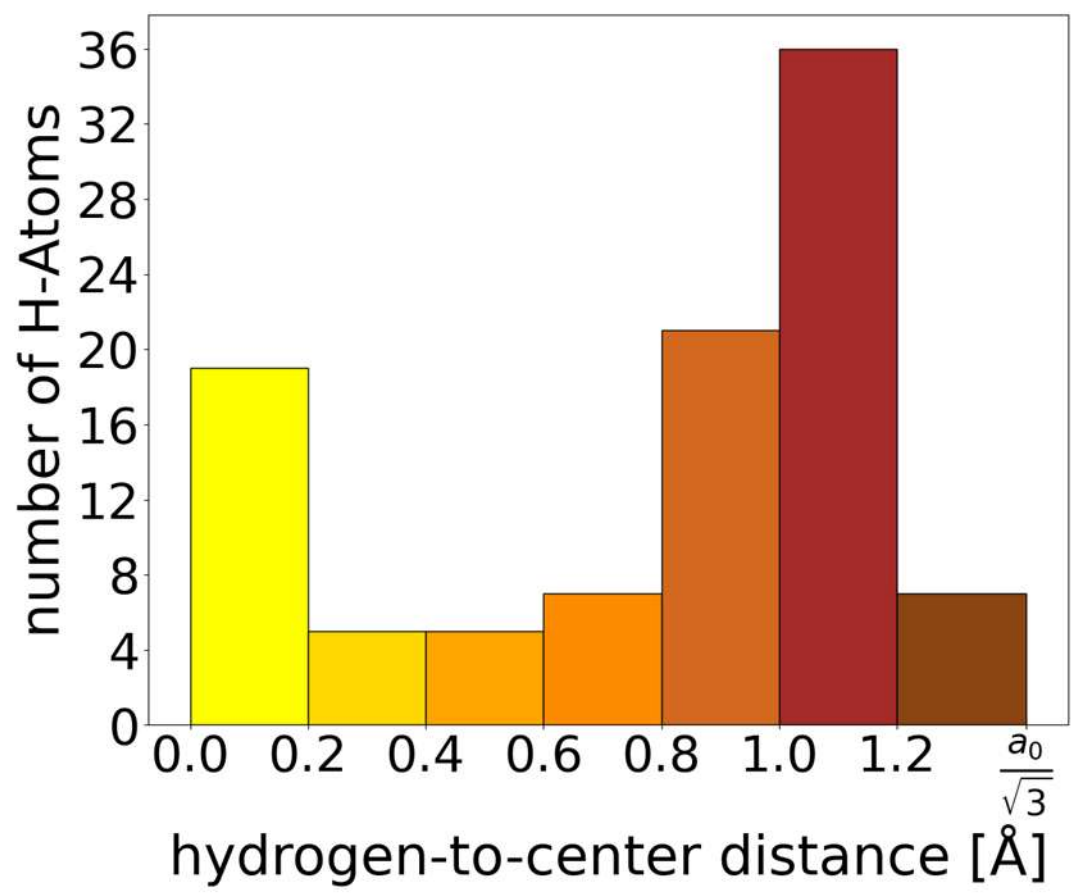

Figure S35 Histogram showing the distribution of the distances between the hydrogen atoms of ***P*-2** (and ***M*-2**) and the centers of the hexagons in the graphene cutout.

6.2 Registry optimization of 3

Enantiomeric input structures of **3** (***P*-3** and ***M*-3**) were prepared by geometry optimization using the GFN-FF method with implicit solvation on graphene with fixed coordinates (side groups were reduced to methoxy units). The optimized backbones were placed on a larger graphene sheet. The combined structures were used as inputs for registry optimization and the input of the ***P*-3** enantiomer is shown in Fig. S36.

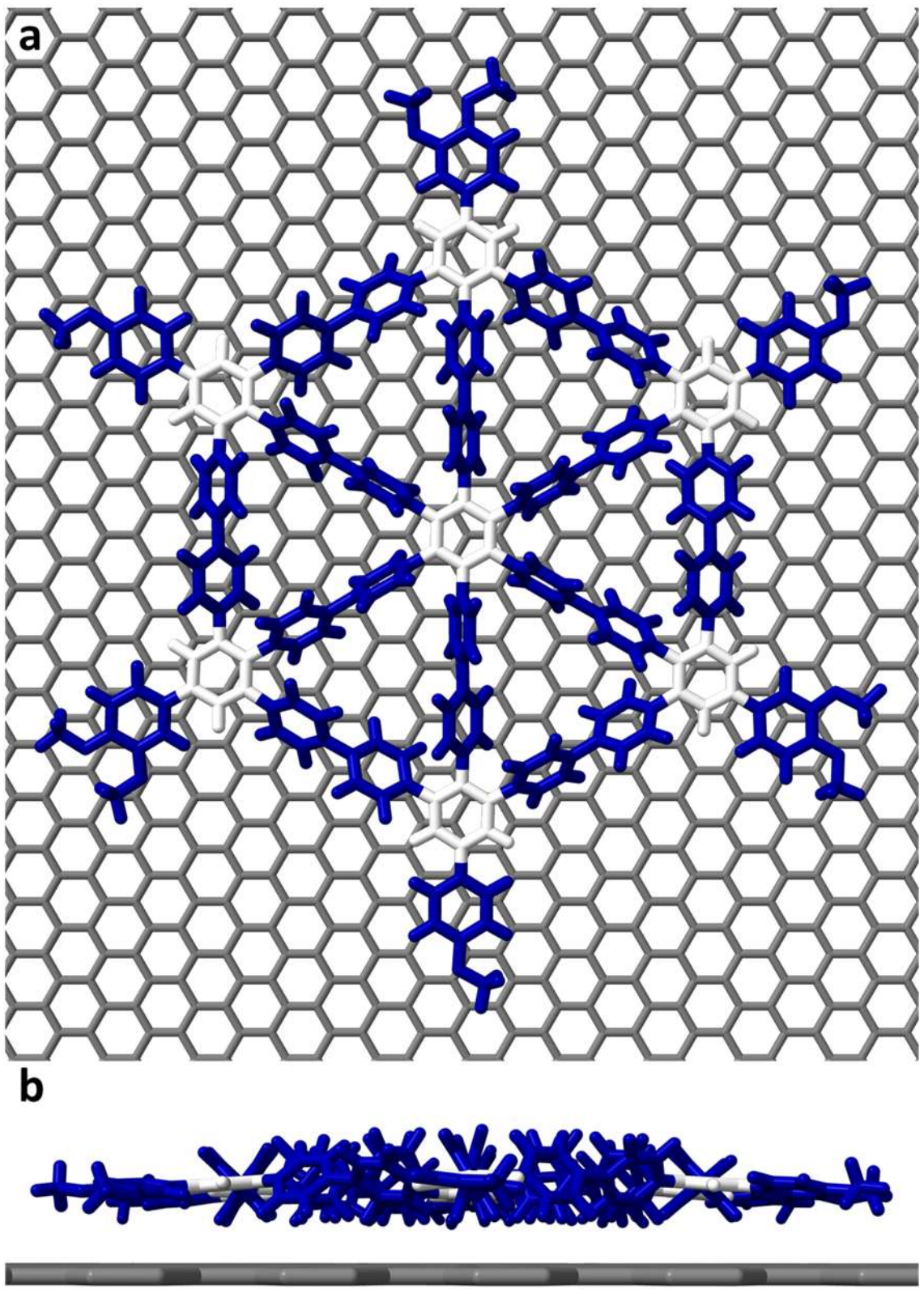

Figure S36 (a) Front and (b) side view of a geometry optimized backbone of ***P*-3** oriented along the zigzag axis of a graphene sheet.

Using the registry optimization workflow, we found a registry configuration for ***P*-3** which is similar to ***P*-1**. We considered downward oriented hydrogens of the strongly tilted spoke/rim phenylene units and of alkyl groups as relevant. Phenyls carrying alkoxy groups are weakly tilted out of the molecular plane. Only the phenylene spoke units contribute to the registry count (cf. Fig. S37). The preferred registry orientation for ***P*-3** of $\gamma(c,d) = -(7 \pm 2)°$ (cf. Fig. S37) is identical to the orientation observed by STM. Using the ***M*-3** enantiomer as input for registry optimization yielded a preferred registry orientation with inverted sign.

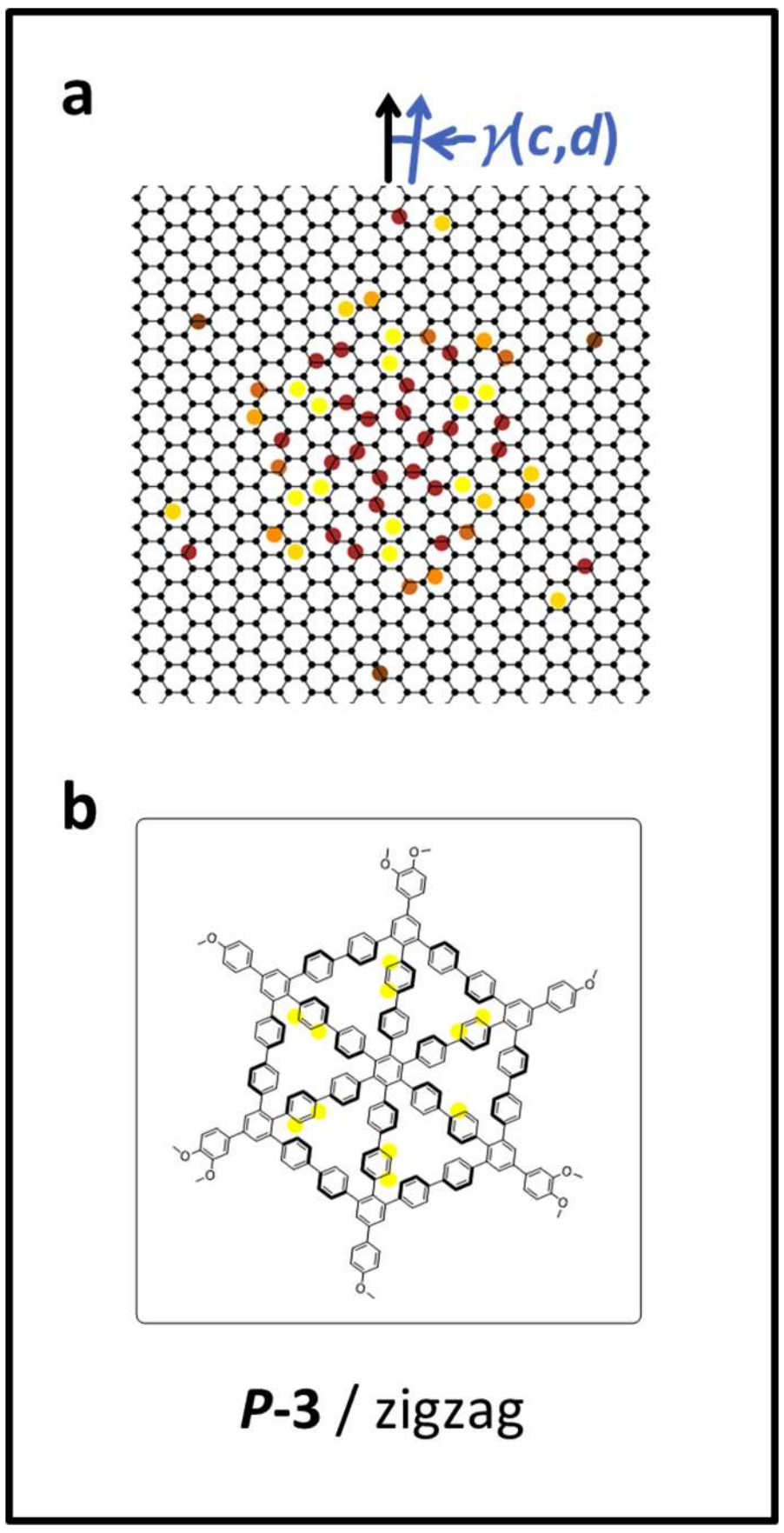

Figure S37 (a) 2D plot of an optimized molecular registry of ***P*-3** on a graphene cutout; the color gradient (light to dark) indicates increasing distances between the hydrogen atoms and the centers of the hexagons with a_0 = 2.46 Å, *i.e.* the lattice vector of graphene; all intervals are left-closed, right-open; (b) Chemical structure of the absolute conformation of ***P*-3** with registry positions highlighted in yellow (hydrogen-to-center distance ≤ 0.2 Å).

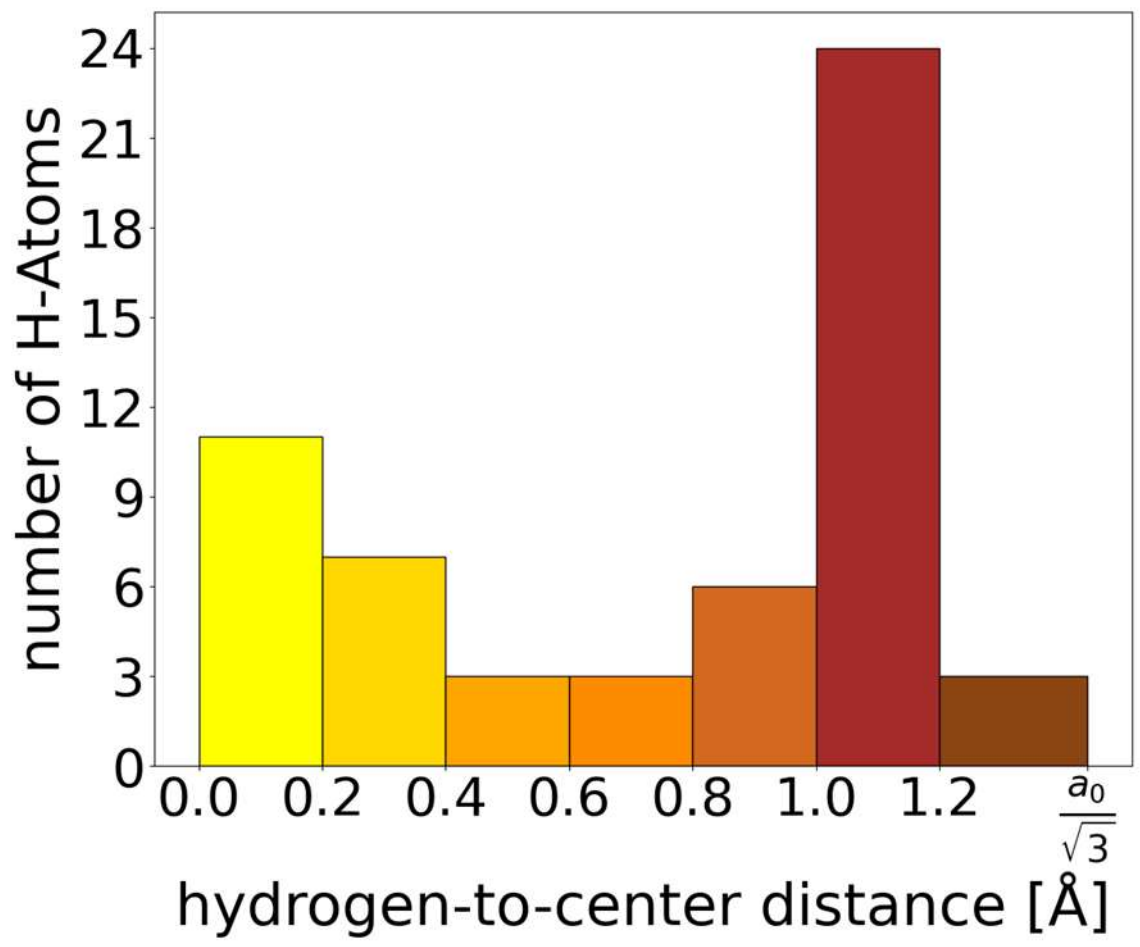

Figure S38 Histogram showing the distribution of the distances between the hydrogen atoms of ***P*-3** and the centers of the hexagons in the graphene cutout.

6.3 Registry optimization of a reported system

We frequently reported on supramolecular nanopatterns of systems carrying phenyl(ene) units which are tilted out of the molecular plane and towards an underlying substrate. At the time of publishing, the discussed algorithm was not available and we had no workflow to discuss molecular orientation of such systems on graphite. In the following, we revisit one of our reported systems regarding its registry on graphite.

In Ref.[20] we reported on MSWs carrying 3,5-dihexadecyloxybenzyloxy (**MSWa**) or 3,4,5-trihexadecyloxybenzyloxy units (**MSWb**) in the periphery. **MSWb** (cf. Fig. S39) forms a densely packed supramolecular nanopattern with a complex alkoxy chain packing. The backbones adopt two different orientations one relative to zigzag with $\gamma(c,d)$ = +(14 ± 2)° and one relative to armchair with $\gamma(c,d')$ = +(2 ± 2)°. We assume that the alkoxy chain packing drives the adsorption orientation of the MSW, resulting in a less favourable registry of the backbone. The backbone of the MSW carrying less chains however may be able to optimize its interaction with the substrate. For **MSWa** (cf. Fig. S39), we reported an orientation *c* of the MSW backbone relative to an HOPG zigzag direction d_1 of $\gamma(c,d_1)$ = +(7 ± 2)°.

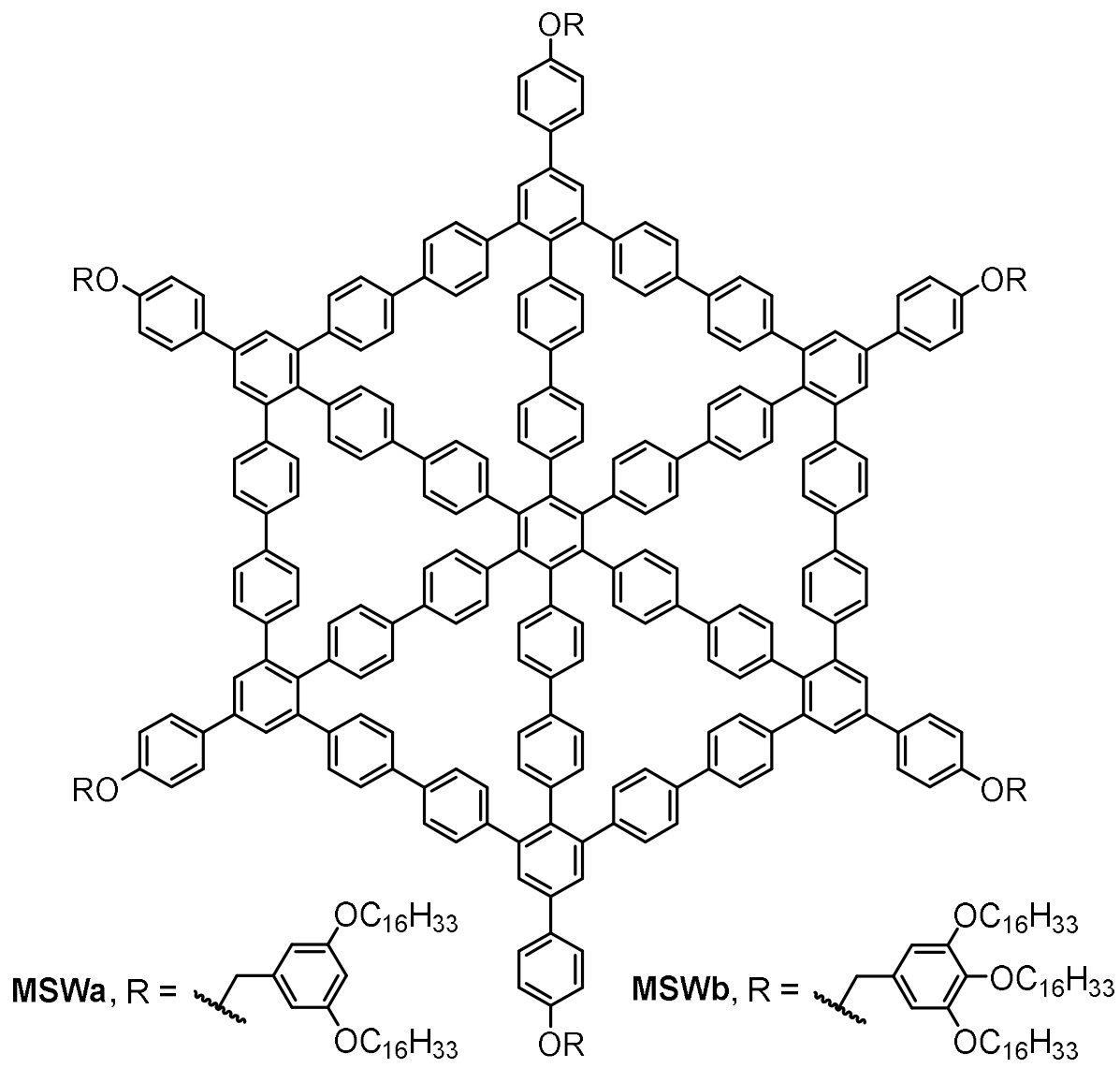

Figure S39 Chemical structures of MSWs carrying either 3,5-dihexadecyloxybenzyloxy (**MSWa**) or 3,4,5-trihexadecyloxybenzyloxy units (**MSWb**) in the periphery.

For registry optimization, enantiomeric input structures of **MSWa** were prepared. One input structure based on the *P*-enantiomer of the oligo-phenyl(ene) spoke and rim backbone is shown in Fig. S40. The structure was obtained by geometry optimization using the GFN-FF method with implicit solvation on a graphene sheet with fixed coordinates (side chains were reduced to methoxy units). Rings colored in light gray are planar while those colored in darker gray are slightly tilted. The optimized backbone was placed on a larger graphene sheet and the combined structure was used for registry optimization (cf. Fig. S41).

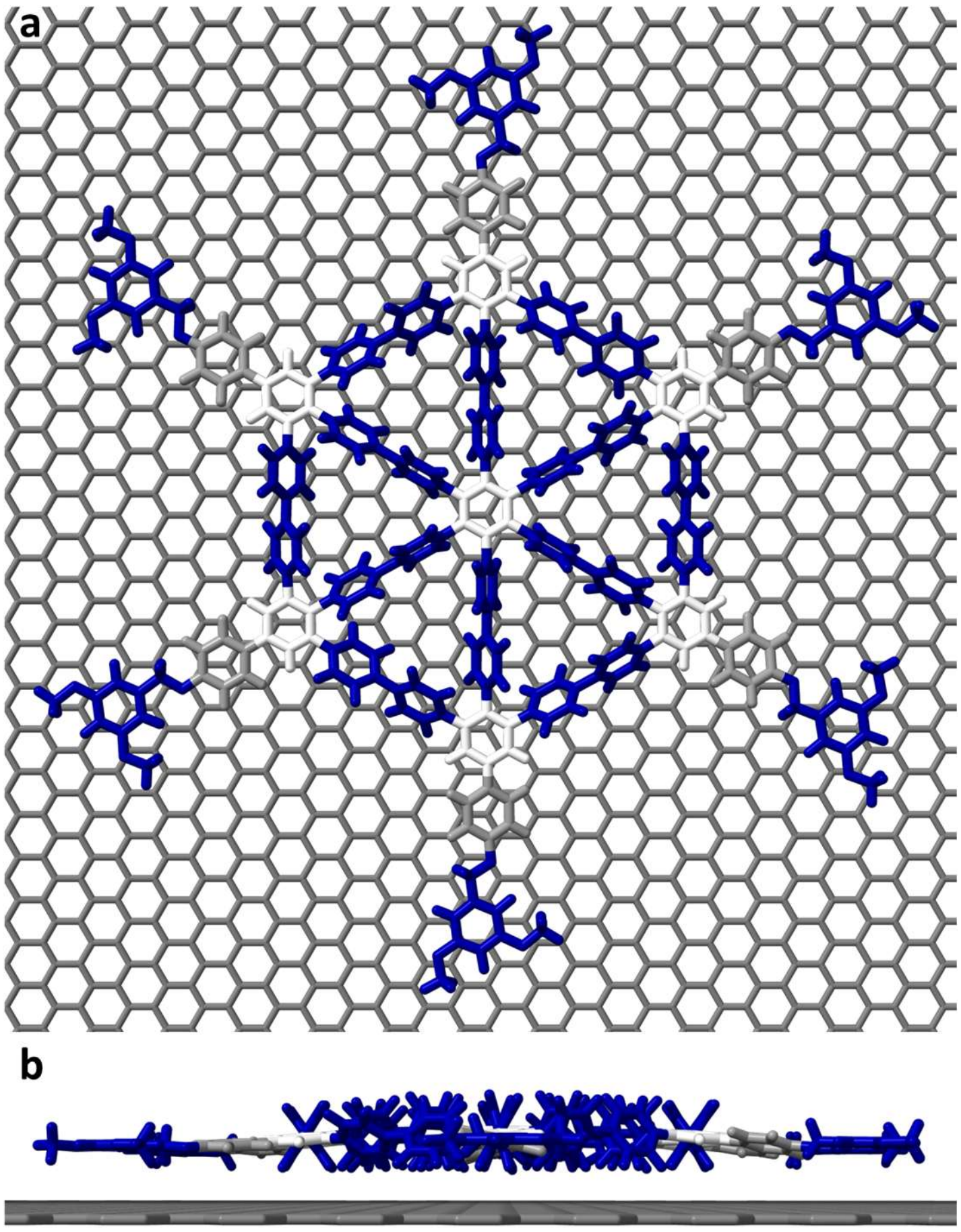

Figure S40 (a) Front and (b) side view of a geometry optimized backbone of the *P*-enantiomer of the discussed **MSWa** oriented along the zigzag axis of a graphene sheet.

Using the registry optimization workflow, we found a registry configuration similar to ***M*-1**. We considered downward oriented hydrogens of the strongly tilted spoke/rim phenylene units and of alkyl groups as relevant. Phenyls carrying dendritic side groups are only weakly tilted out of the molecular plane and their downward oriented hydrogens mostly exhibit a larger distance to graphene than hydrogens of the alkyl groups. Only phenylene units of the spokes contribute to the registry count (cf. Fig. S41). The preferred registry orientation for **MSWa** of $\gamma(c,d)$ = +(7 ± 2)° (cf. Fig. S41) is identical to the orientation observed by STM of $\gamma(c,d_1)$ = +(7 ± 2)° (cf. Ref. 20). Using the *P* enantiomer as input for registry optimization led to an inverted sign of the preferred registry orientation. The MSW showed different image contrasts throughout multiple STM measurements with a variation of brightness distribution contrary to MSWs **1**, **2** and **3**. Thus, we found no correlation of STM contrast features and registry positions in this case.

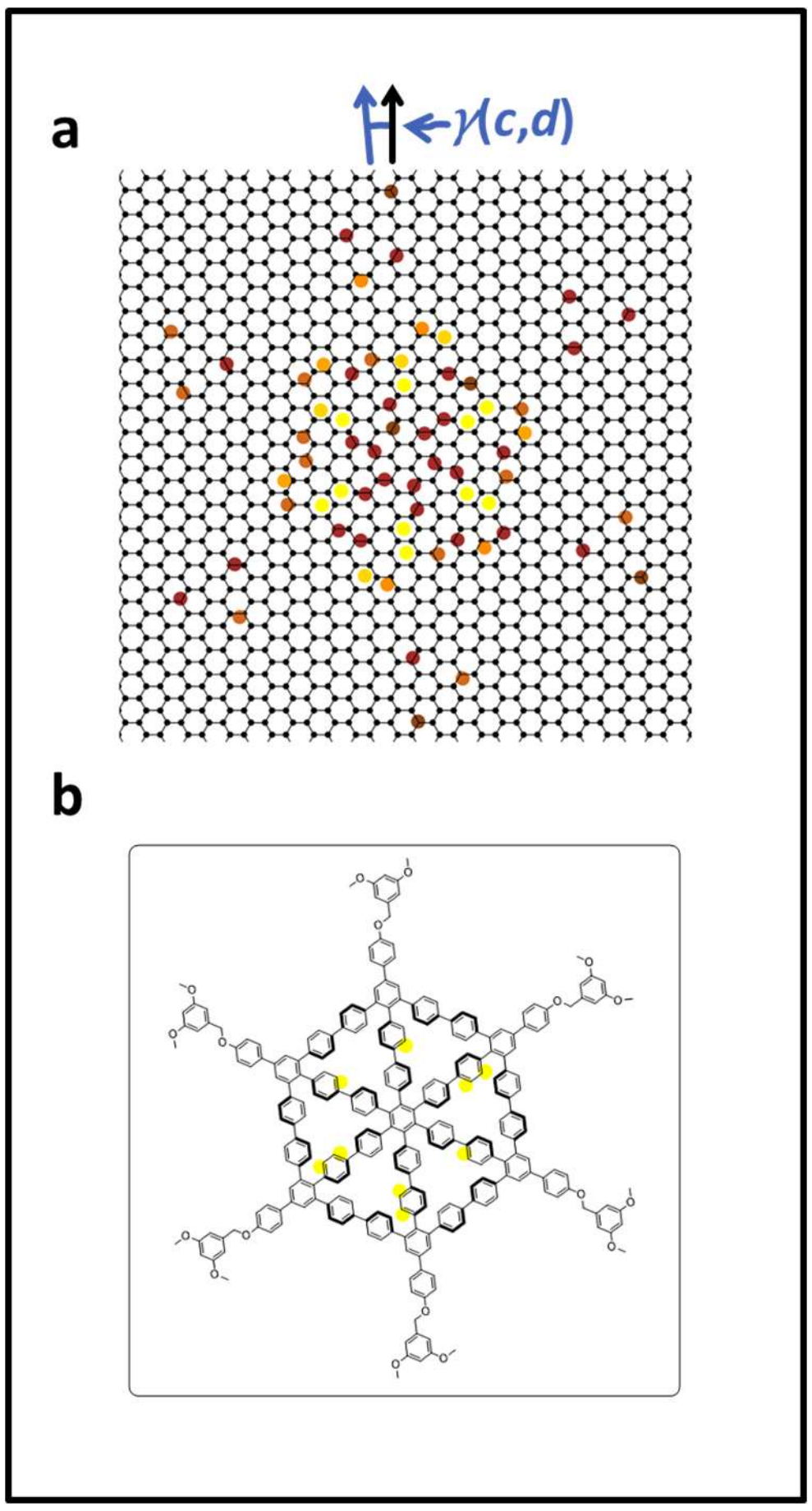

Figure S41 (a) 2D plot of an optimized molecular registry of the discussed **MSWa** on a graphene cutout; the color gradient (light to dark) indicates increasing distances between the hydrogen atoms and the centers of the hexagons with a_0 = 2.46 Å, *i.e.* the lattice vector of graphene; all intervals are left-closed, right-open; (b) Chemical structure of the absolute conformation of the geometry optimized backbone with registry positions highlighted in yellow (hydrogen-to-center distance ≤ 0.2 Å).

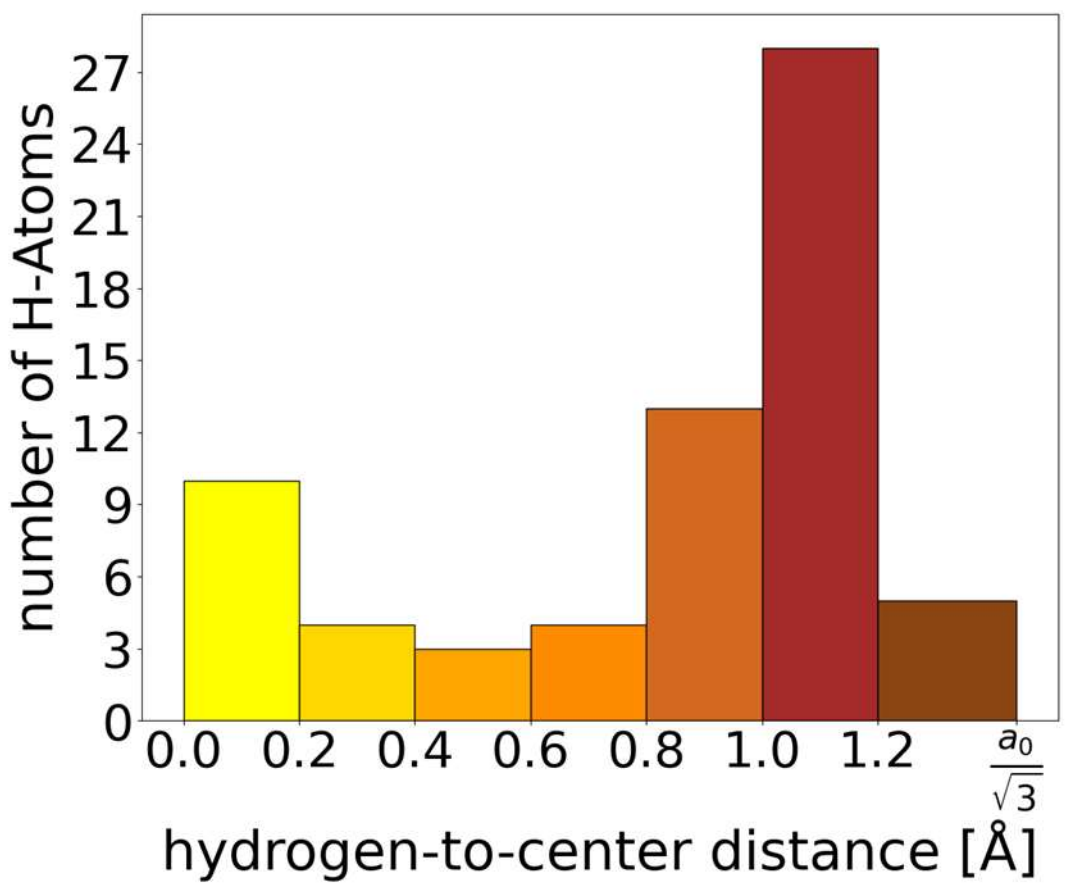

Figure S42 Histogram showing the distribution of the distances between the hydrogen atoms of the discussed **MSWa** and the centers of the hexagons in the graphene cutout.

7 Experimental vs. theoretical backbone/graphite orientations

The applied registry optimization workflow was employed to determine the absolute conformation of adsorbates at their preferred registry orientation on graphite.

Experimentally, the orientation of the molecular backbone relative to the graphite lattice – specifically with respect to the zigzag *d* and armchair *d*′ axes – is accessible from distinct contrast features in high resolution STM images.

Within the theoretical framework, the backbone/graphite orientation emerges as an output parameter associated with the highest registry score of hydrogen atom spikes detected at the graphite hexagon center pockets.

For multiple systems, the theoretically predicted backbone/graphite orientations were consistent with those observed by STM experiments. A quantitative comparison is provided in Table S4. In the case of MSW **3** and the literature MSWs (**MSWa**), we observed the packing of an individual atropisomer. However, the theoretical approach predicts orientations of opposite sign for two atropisomers, which are expected to adsorb on graphite in equal proportions. Experimentally, only one orientation was observed, likely due to limited STM data.

Table S4 Comparison of experimentally vs. theoretically obtained backbone/graphite orientations (*i.e*. the angles $\gamma(c,d)$ and $\gamma(c,d')$).

Adsorbate	Experiment		Theory	
	$\gamma(c,d)$ [°]	$\gamma(c,d')$ [°]	$\gamma(c,d)$ [°]	$\gamma(c,d')$ [°]
**1**	±(7 ± 2)	±(11 ± 2)	±(7 ± 2)	±(11 ± 2)
**2**	±(7 ± 2)	–	±(7 ± 2)	–
**3**	−(7 ± 2)	–	±(7 ± 2)	–
**MSWa** in Ref. 20	+(7 ± 2)	–	±(7 ± 2)	–
**MSWb** in Ref. 20*	+(14 ± 2)	+(2 ± 2)	±(7 ± 2)	–

* The alkoxy chain packing determines the orientation of the backbone.

8 Synthesis

8.1 Synthesis of 1

Figure S43 Reagents and conditions: a) KOH, EtOH, r.t., 4 d, 61 %; b) triphenylmethanol, acetic anhydride, HBF_4, 10 min, 60 °C, then 15 min, 120 °C, then 2 d, 100 °C, 82 % with impurities; c) 4-(trifluoromethyl)benzoic anhydride, 170 °C, 5 h, 28 %, d) $PdCl_2(PPh_3)_2$, PPh_3, CuI, CPD*i*PS-acetylene, THF:piperidine (1:2), r.t., 18 h, 91 %; e) **9**, $PdCl_2(PPh_3)_2$, PPh_3, CuI, TBAF, THF:piperidine (1:2), r.t., 18 h, 71 %; f) $Co_2(CO)_8$, toluene, 90 °C, 5 h, 54 %; g) $Ni(COD)_2$, bipyridine, THF, COD, microwave, 120 °C, 12 min, 60 %.

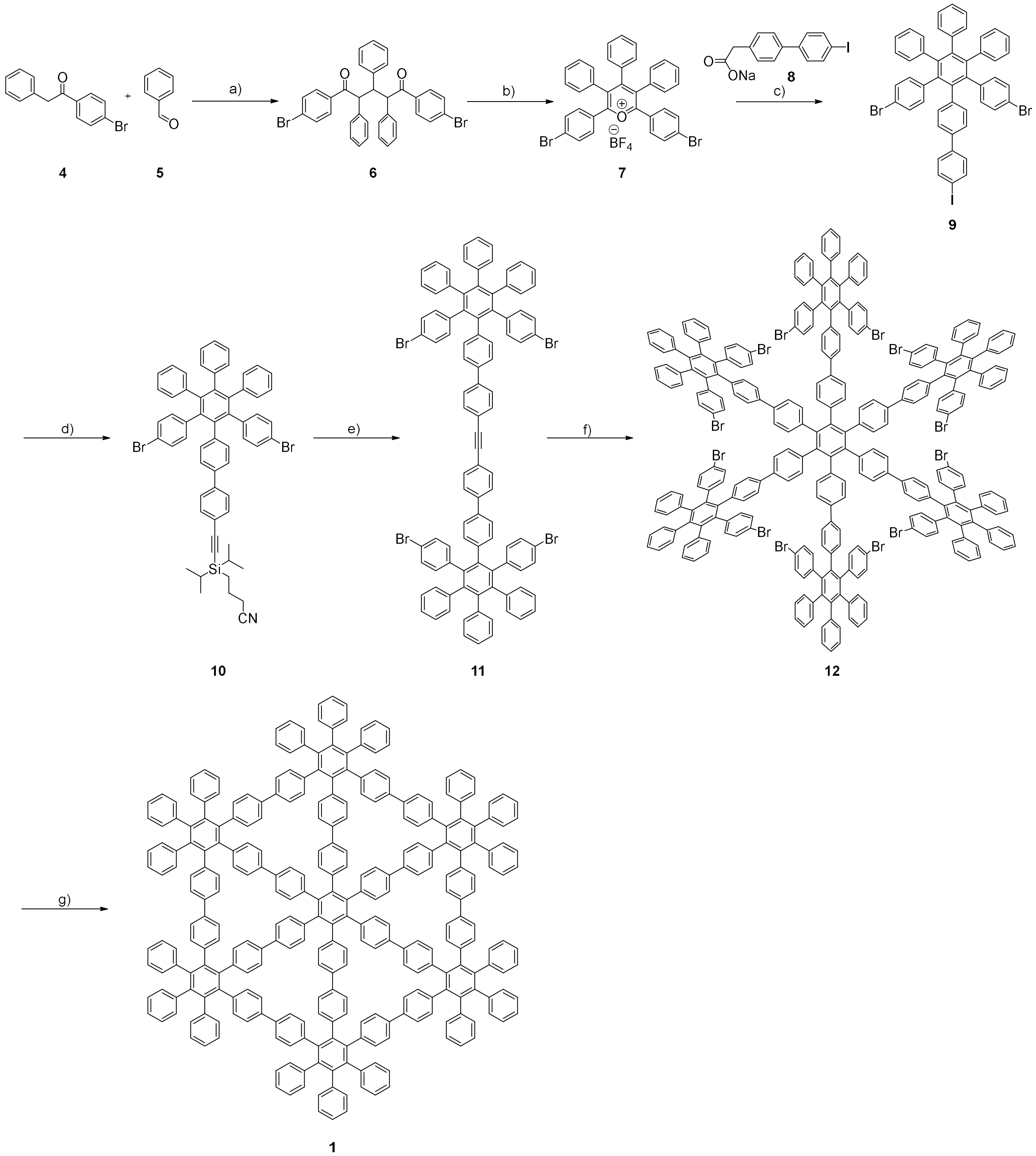

6

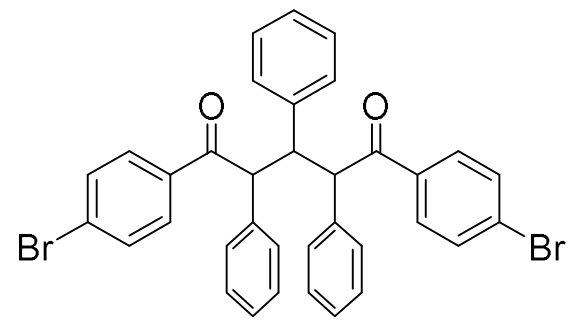

4 (3.31 g, 12.0 mmol) and KOH (1.69 g, 30.1 mmol) were added to EtOH (75 mL). Then, **5** (0.61 mL, 6.02 mmol) was added and the suspension was stirred at r.t. for 4 d. The suspension was filtered and the solid was washed with MeOH. **6** (2.35 g, 3.69 mmol, 61 %) was obtained as crème-colored solid.

Chemical formula: $C_{35}H_{26}Br_2O_2$. **^{1}H NMR** (500 MHz, $CDCl_3$, r.t.): δ [ppm] = 7.56 (d, J = 8.8 Hz, 2H), 7.44 (d, J = 8.8 Hz, 2H), 7.40–7.35 (m, 6H), 7.12 (t, J = 7.7 Hz, 2H), 7.07–7.04 (m, 3H), 7.02–6.94 (m, 7H), 6.92–6.88 (m, 1H), 5.14 (d, J = 10.4 Hz, 1H), 4.97 (d, J = 7.88 Hz, 1H), 4.84 (dd, J = 7.87 Hz, 1H). **^{13}C NMR** (125.8 MHz, $CDCl_3$, r.t.): δ [ppm] = 197.6, 197.3, 140.2, 136.5, 136.0, 135.9, 135.2, 131.8, 131.3, 130.3, 130.0, 129.9, 129.8, 129.1, 128.2, 128.1, 127.8, 127.6, 127.1, 126.4, 59.1, 56.2, 50.9. **MS** (ESI+), m/z (%) = 639.0 (6) $[M+H]^+$, 282.3 (100) $[C_{22}H_{18}]^+$, 273.2 (65) $[C_{21}H_{20}+H]^+$.

7

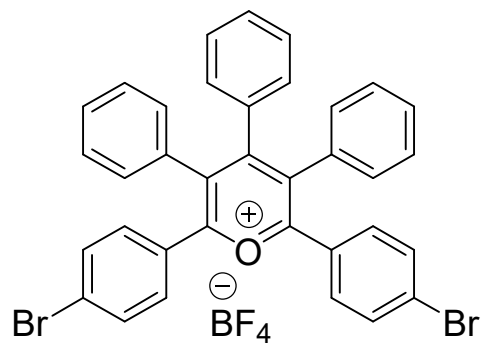

Under Ar atmosphere, **6** (2.36 g, 3.69 mmol), triphenylmethanol (1.16 g, 4.44 mmol) and acetic anhydride (12.3 mL) were placed in a dry Schlenk tube and heated to 60 °C for 10 min. After addition of HBF_4 (2.3 mL, 48 % aq. solution, 17.8 mmol), the reaction mixture was heated to 120 °C for 15 min and afterwards stirred at 100 °C for 2 d. The suspension was filtered and the filtrate was concentrated and added to diethylether (150 mL). The precipitated solid was filtered off and washed with diethylether. **7** (2.16 g, 3.08 mmol, 82 %, however with minor impurities) was obtained as orange solid.

Chemical formula: $C_{35}H_{23}Br_2OBF_4$. **^{1}H NMR** (500 MHz, DMSO-d_6, r.t.): δ [ppm] = 7.48–7.45 (m, 2H), 7.39–7.34 (m, 4H), 7.08–7.06 (m, 2H), 7.03–6.97 (m, 7H), 6.88–6.81 (m, 8H). **^{13}C NMR** (125.8 MHz, DMSO-d_6, r.t.): δ [ppm] = 144.8, 142.3, 137.6, 137.2, 136.8, 135.7, 134.6, 131.4, 130.2, 130.2, 130.1, 129.3, 127.4, 126.8, 121.0. **MS** (ESI+), m/z (%) = 619.0 (100) $[M-BF_4]^+$, 541.1 (4) $[M-BF_4-Br]^+$.

9

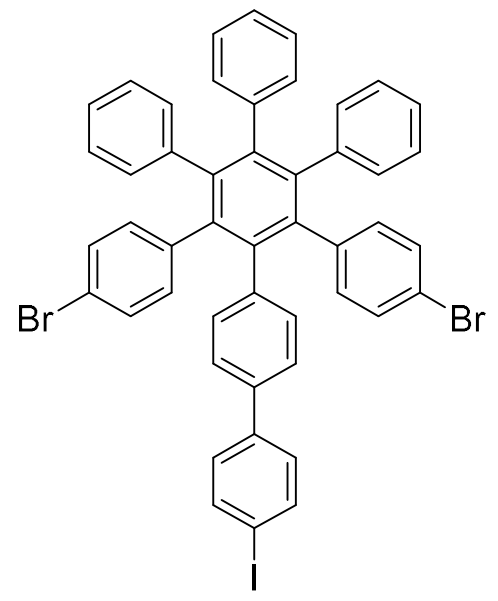

Under Ar atmosphere, 4-(trifluoromethyl)benzoic anhydride[64] (0.579 g, 1.59 mmol), **7** (0.161 g, 0.227 mmol) and **8**[20] (82.2 mg, 0.227 mmol) were stirred at 170 °C for 5 h. After the mixture was cooled to r.t., the residue was dissolved in DCM:CH (1:3) and purified by column chromatography (DCM:CH = 1:3; R_f = 0.45). **9** (56.4 mg, 63.1 µmol, 28 %) was obtained as white solid.

Chemical formula: $C_{48}H_{31}Br_2I$. **^{1}H NMR** (500 MHz, $CDCl_3$, r.t.): δ [ppm] = 7.69 (d, J = 8.5 Hz, 2H), 7.20 (d, J = 8.5 Hz, 2H), 7.13 (d, J = 8.4 Hz, 2H), 7.00 (d, J = 8.4 Hz, 4H), 6.91–6.83 (m, 11H), 6.81–6.77 (m, 6H), 6.70 (d, J = 8.5 Hz, 4H). **^{13}C NMR** (125.8 MHz, $CDCl_3$, r.t.): δ [ppm] = 141.0, 140.9, 140.3, 140.2, 140.0, 139.6, 139.5, 139.4, 139.3, 137.7, 137.0, 133.1, 132.0, 131.4, 131.3, 130.1, 128.7, 127.0, 126.7, 125.6, 125.5, 125.4, 119.7, 92.9. **MS** (MALDI-TOF pos, DCTB): m/z (%) = 893.9 (100) $[M]^{+\bullet}$.

10

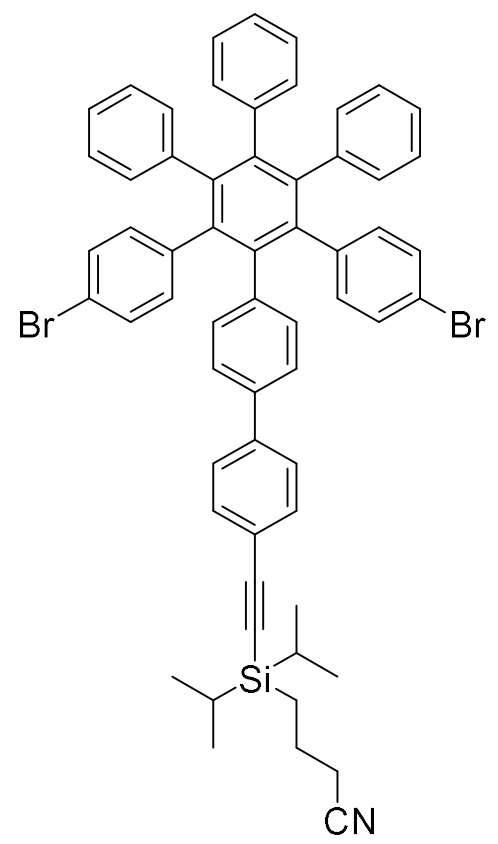

9 (129 mg, 0.144 mmol) was dissolved in THF (13 mL) and piperidine (26 mL) and purged with Ar for 1 h. $PdCl_2(PPh_3)_2$ (6.0 mg, 8.64 µmol), PPh_3 (6.8 mg, 25.9 µmol) and CuI (27.4 mg, 0.144 mmol) were added. Then, CPD*i*PS-acetylene (32.8 mg, 0.157 mmol)[65] was added slowly and the reaction mixture was stirred at r.t. for 18 h. The reaction mixture was diluted with DCM and HCl (10 %). After extraction of the aqueous layer with DCM, the combined organic phases were washed with water, aq. HCl (10 %) and brine, and dried over $MgSO_4$. The crude product was purified by column chromatography (DCM:CH = 2:1; R_f = 0.49). **10** (127 mg, 0.131 mmol, 91 %) was obtained as white solid.

Chemical formula: $C_{60}H_{51}Br_2NSi$. **^{1}H NMR** (500 MHz, $CDCl_3$, r.t.): δ [ppm] = 7.47–7.40 (m, 4H), 7.18 (d, J = 8.5 Hz, 2H), 7.00 (d, J = 8.5 Hz, 4H), 6.90–6.82 (m, 11H), 6.80–6.77 (m, 6H), 6.71 (d, J = 8.5 Hz, 4H), 2.42 (t, J = 7.0 Hz, 2H), 1.90–1.82 (m, 2H), 1.12–1.04 (m, 14H), 0.86–0.81 (m, 2H). **^{13}C NMR** (125.8 MHz, $CDCl_3$, r.t.): δ [ppm] = 141.0, 140.9, 140.3, 140.2, 139.8, 139.7, 139.4, 139.2, 137.1, 133.1, 132.5, 131.8, 131.4, 130.0, 127.0, 126.7, 126.6, 125.7, 125.6, 125.6, 121.8, 119.8, 119.7, 108.0, 90.1, 67.9, 21.3, 20.8,

18.4, 18.0, 11.8, 9.8. **MS** (MALDI-TOF pos, DCTB): *m/z* (%) = 996.2 (31) [M+Na]$^+$, 973.2 (2) [M]$^{+•}$, 936.0 (20) [M-C_3H_8+Li]$^+$, 930.2 (100) [M-C_3H_7]$^+$.

11

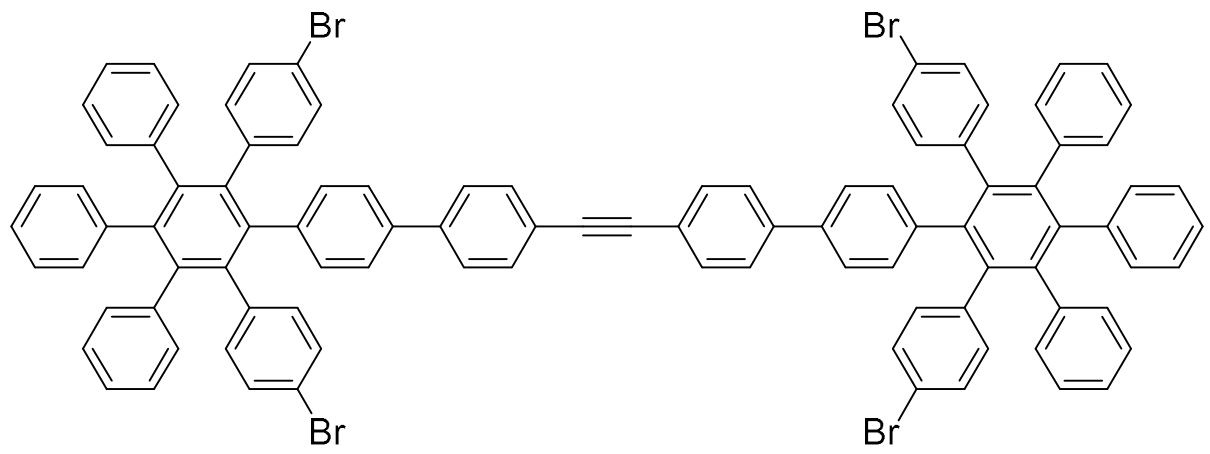

9 (223 mg, 0.248 mmol) and **10** (262 mg, 0.269 mmol) were dissolved in THF (8 mL) and piperidine (16 mL) and purged with Ar for 1 h. $PdCl_2(PPh_3)_2$ (10.5 mg, 14.9 µmol), CuI (33.1 mg, 0.175 mmol) and PPh_3 (11.8 mg, 44.9 µmol) were added. Then, TBAF (0.5 mL, 1 M in THF, 0.5 mmol) was added and the reaction mixture was stirred at r.t. for 18 h. The reaction mixture was diluted with DCM and aq HCl (10 %). After extraction of the aqueous layer with DCM, the combined organic phases were washed with water, HCl (10 %) and brine, and dried over $MgSO_4$. The crude product was precipitated by the addition of THF and was filtered off. Purification by column chromatography (DCM) gave the product **11** (0.275 mg, 0.175 mmol, 71 %) as white-yellow solid.

Chemical formula: $C_{98}H_{62}Br_4$. **^{1}H NMR** (500 MHz, CD_2Cl_2, r.t.): *δ* [ppm] = 7.54 (d, *J* = 8.5 Hz, 4H), 7.50 (d, *J* = 8.6 Hz, 4H), 7.25 (d, *J* = 8.5 Hz, 4H), 7.02 (d, *J* = 8.5 Hz, 8H), 6.94 (d, *J* = 8.4 Hz, 4H), 6.92–6.90 (m, 12H), 6.89–6.84 (m, 18H), 6.79 (d, *J* = 8.5 Hz, 8H). **^{13}C NMR** (125.8 MHz, CD_2Cl_2, r.t.): *δ* [ppm] = 141.2, 141.1, 140.8, 140.7, 140.5, 140.0, 140.0, 139.9, 139.6, 137.3, 133.5, 132.1, 131.6, 131.6, 131.5, 130.1, 127.2, 127.0, 127.0, 125.9, 125.8, 125.7, 122.4, 120.1, 90.2. **MS** (MALDI-TOF pos, DCTB): *m/z* (%) = 1558.2 (100) [M]$^{+•}$.

12

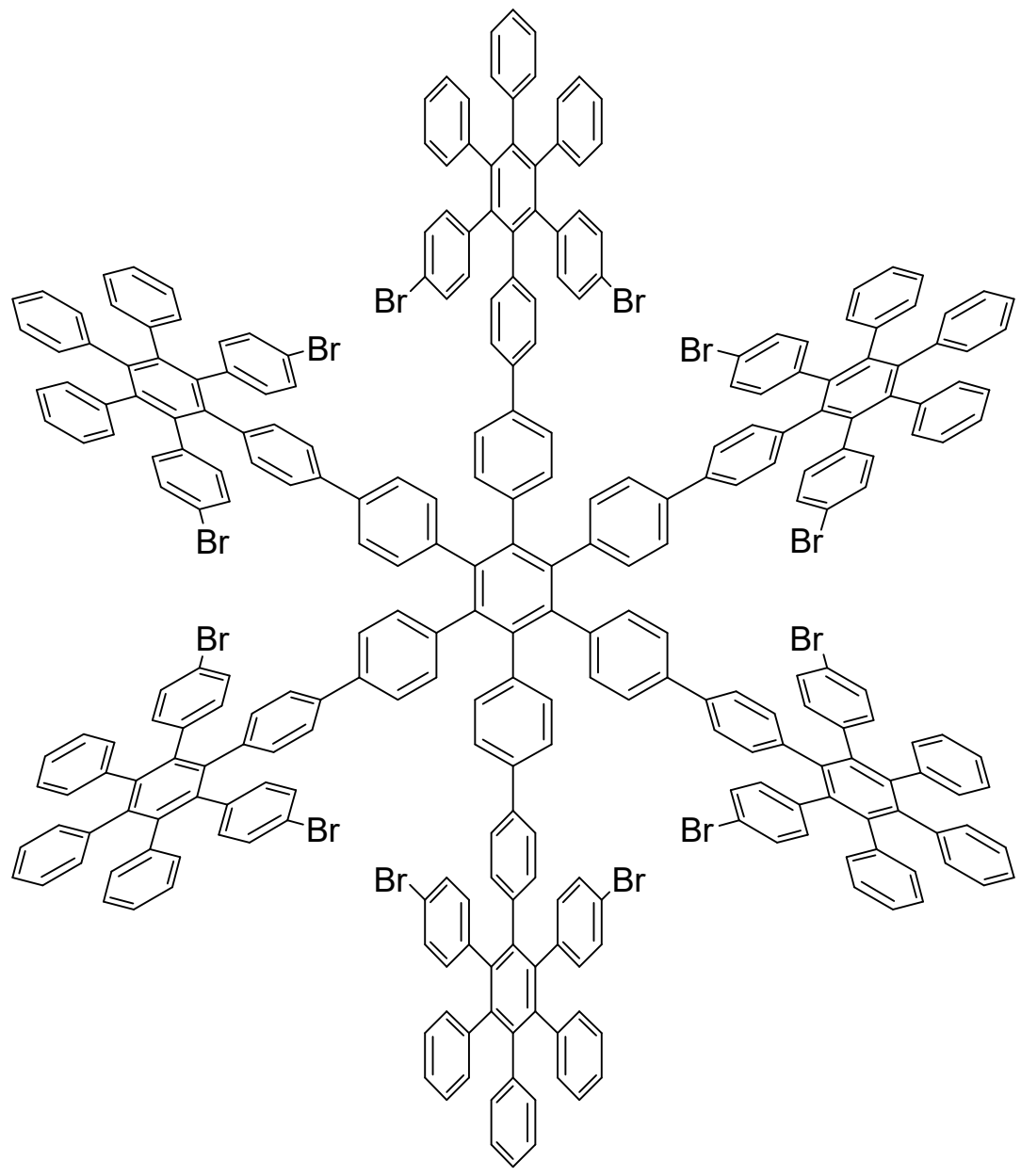

11 (275 mg, 0.175 mmol) was dissolved in toluene (12 mL) in a dry Schlenk tube and purged with Ar for 1 h. Then, $Co_2(CO)_8$ (12.0 mg, 35.1 µmol) was added and the reaction mixture was stirred at 90 °C for 5 h. After cooling to r.t. the reaction mixture was diluted with DCM and water. After extraction of the aqueous layer with DCM, the combined organic phases were washed with water, aq HCl (10 %) and brine, and dried over $MgSO_4$. The crude product was purified by column chromatography (DCM) and then precipitated and filtered by the addition of MeOH. The solid was then purified by recGPC and the **12** (146 mg, 31.5 µmol, 54 %) was obtained as white-yellow solid.

Chemical formula: $C_{294}H_{186}Br_{12}$. **^{1}H NMR** (700 MHz, $CDCl_3$, r.t.): *δ* [ppm] = 7.00–6.93 (m, 48H), 6.88–6.87 (m, 36H), 6.86–6.83 (m, 18H), 6.81–6.78 (m, 48H), 6.73 (d, *J* = 8.4 Hz, 12H), 6.67 (d, *J* = 8.5 Hz, 24H). **^{13}C NMR** (176 MHz, $CDCl_3$, r.t.): *δ* [ppm] = 140.7, 140.7, 140.4, 140.2, 140.2, 139.9, 139.7, 139.2, 138.4, 138.0, 137.2, 133.0, 131.8, 131.7, 131.4, 130.1, 127.0, 126.8, 125.6, 125.5, 125.5, 125.4, 125.2, 125.2, 119.6. **MS** (MALDI-TOF pos, DCTB): *m/z* (%) = 4716.5 (2) $[M+K]^+$, 4700.5 (1) $[M+Na]^+$, 4677.5 (100) $[M]^+$, 4598.6 (6) $[M-Br]^+$.

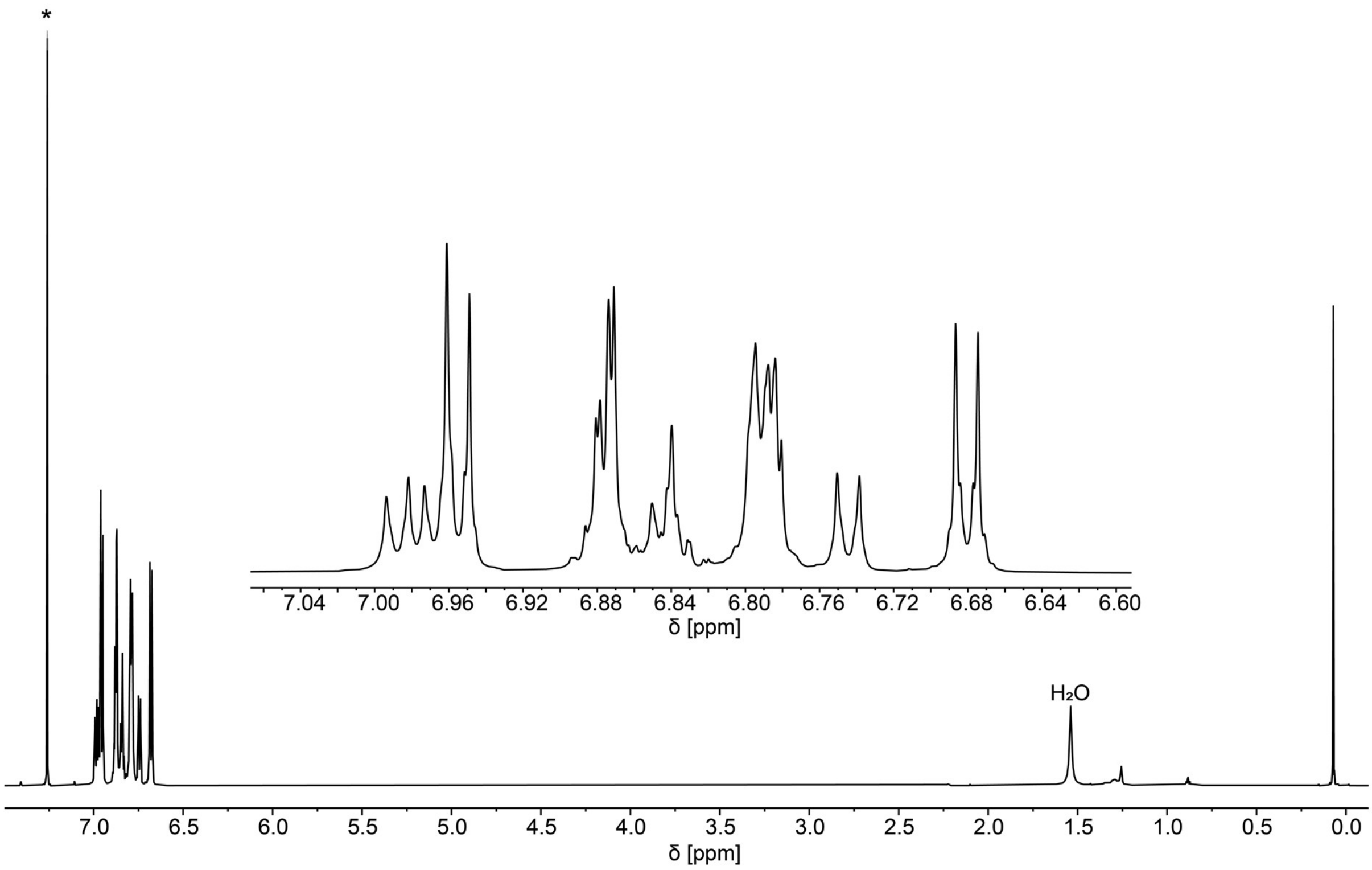

Figure S44 ^{1}H NMR spectra of **12** in $CDCl_3$*.

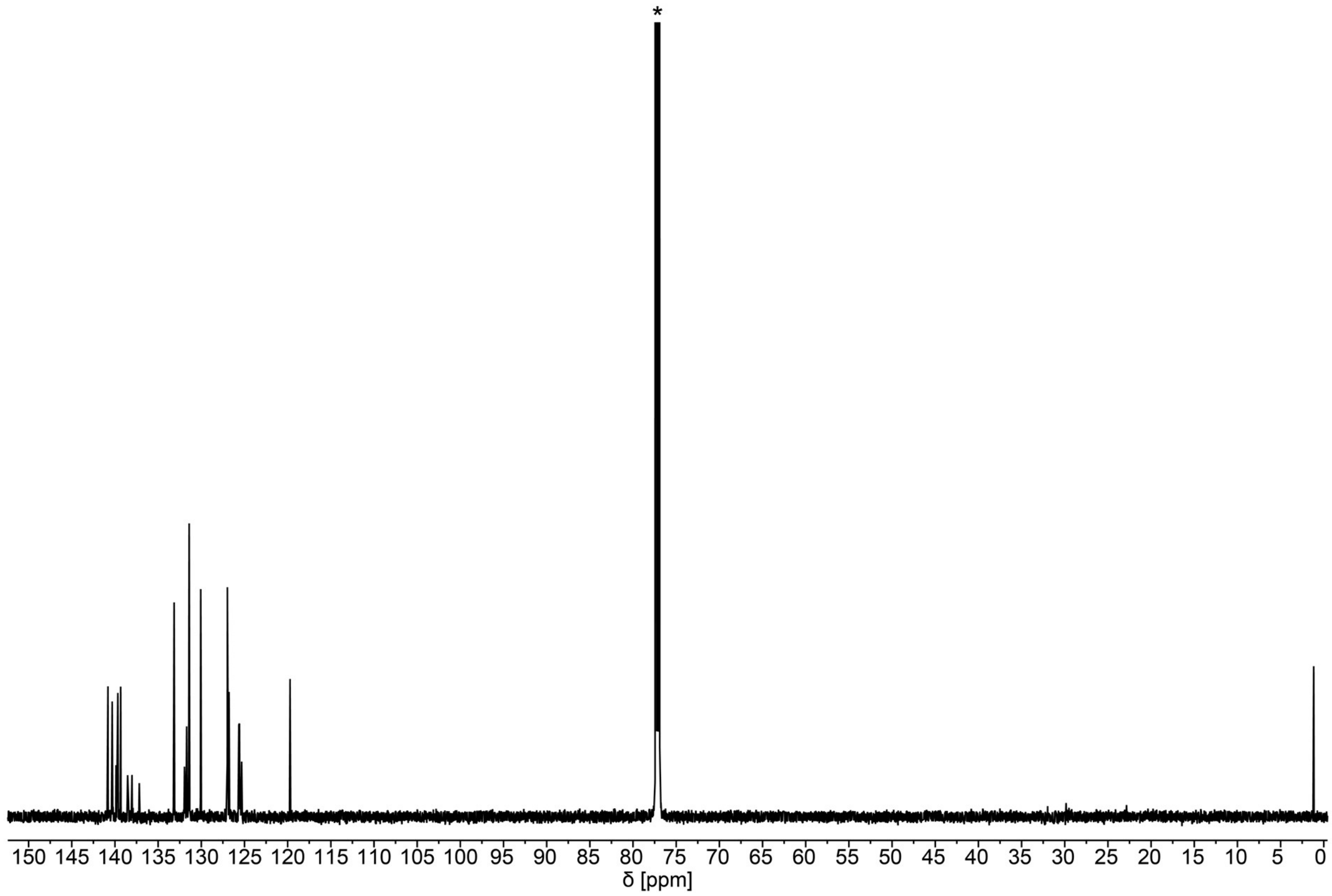

Figure S45 ^{13}C NMR spectra of **12** in $CDCl_3$*.

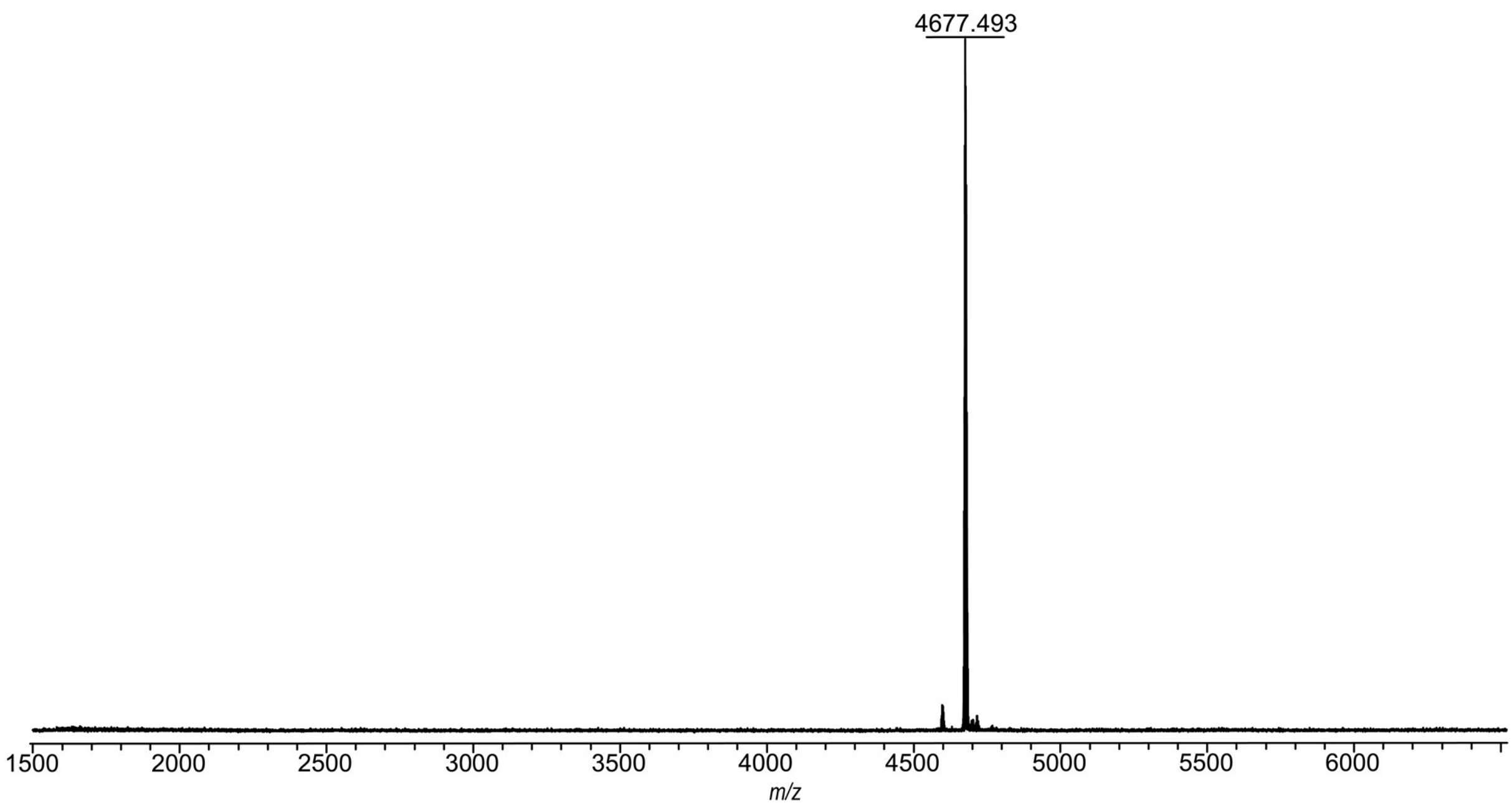

Figure S46 MALDI-TOF mass spectrum of **12** (matrix: DCTB).

1

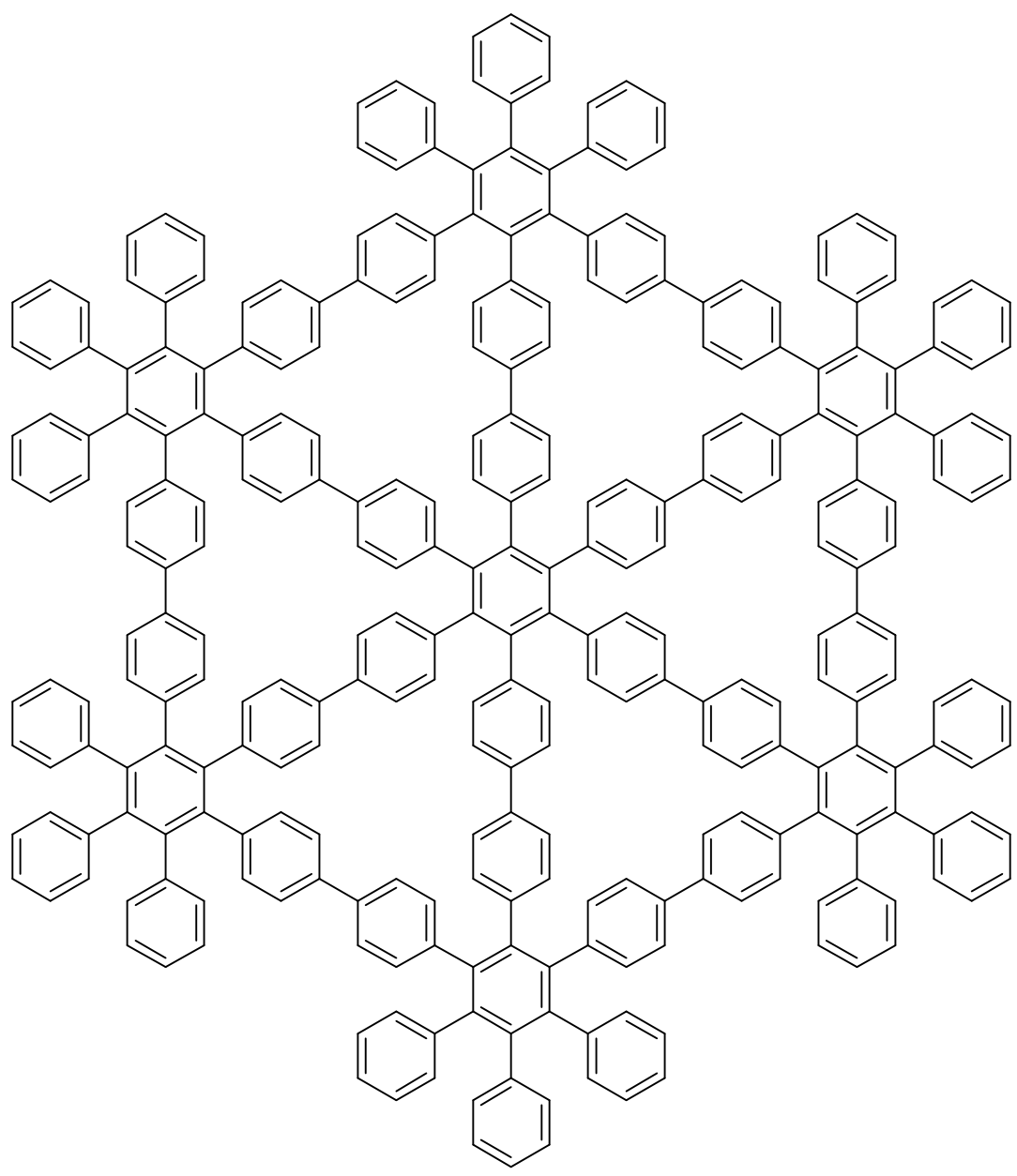

Under an Ar atmosphere, **12** (10.4 mg, 2.22 µmol), Ni(COD)$_2$ (12.0 mg, 43.5 µmol) and bipyridine (6.0 mg, 38.3 µmol) were dissolved in THF (8.00 mL) and COD (0.25 mL) in a microwave tube. The reaction mixture was heated in the microwave to 120 °C for 12 min. Then, the solvent was removed by reduced pressure and DCM and MeOH were added to the crude product. The suspension was filtered and the solid was washed with MeOH. 1,1,2,2-Tetrachloroethane was added to the solid and the suspension was refluxed for 3 h. The precipitate was filtered and the solvent of the filtrate was removed under reduced pressure. **1** (5.1 mg, 1.35 µmol, 60 %) was obtained as white-grey solid.

Chemical formula: $C_{294}H_{186}$. **^{1}H NMR** (500 MHz, $C_2D_2Cl_4$, 80 °C): *δ* [ppm] = 7.07–6.96 (m, 47H), 6.81–6.68 (m, 139H). **MS** (MALDI-TOF pos, DCTB): *m/z* (%) = 3719.5 (100) $[M+H]^+$, 3736.5 (14) $[M+NH_4]^+$. ^{13}C-measurement was not possible due to low solubility of **1**.

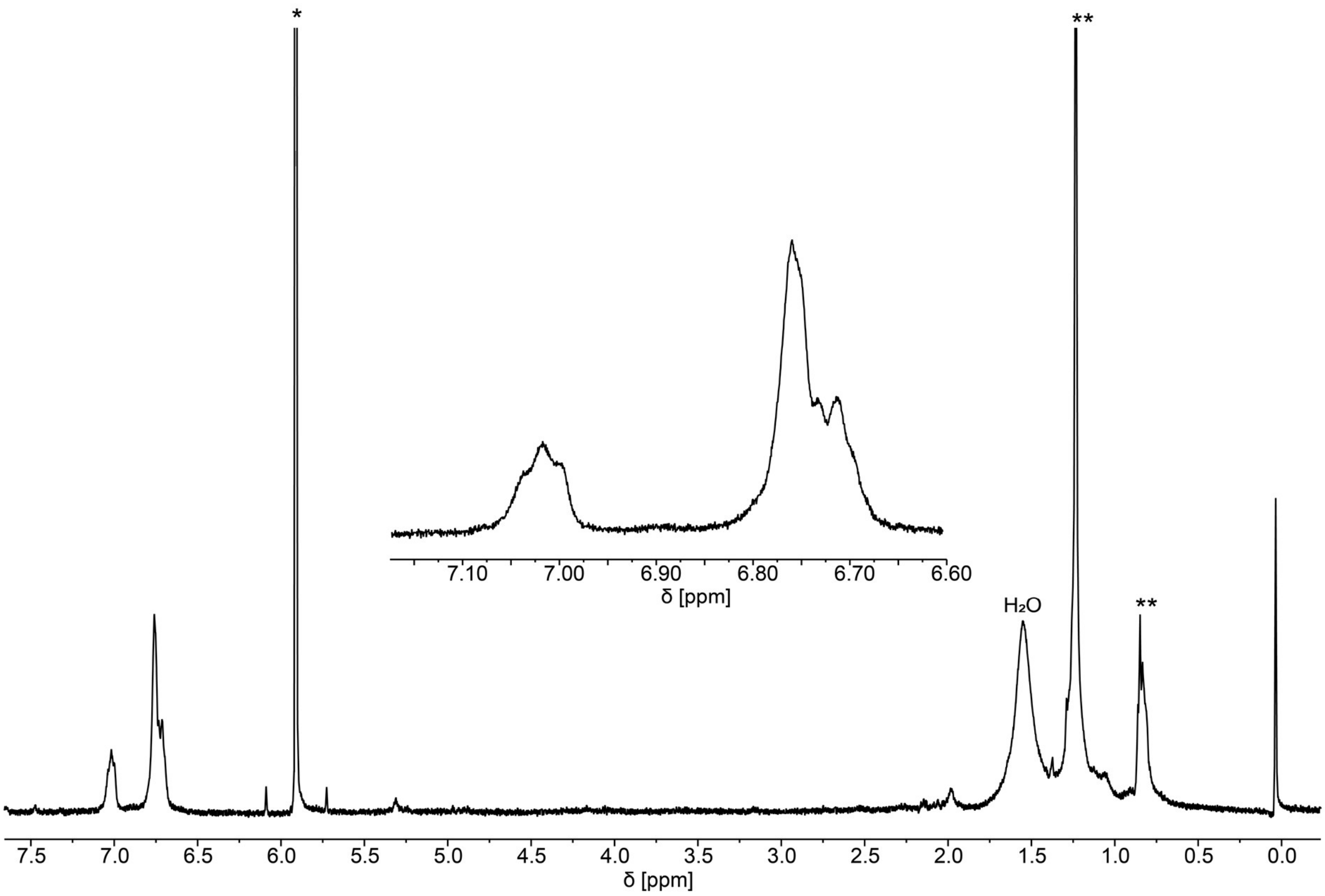

Figure S47 ^{1}H NMR spectra of **1** in $C_2D_2Cl_4$* (**H grease impurities).

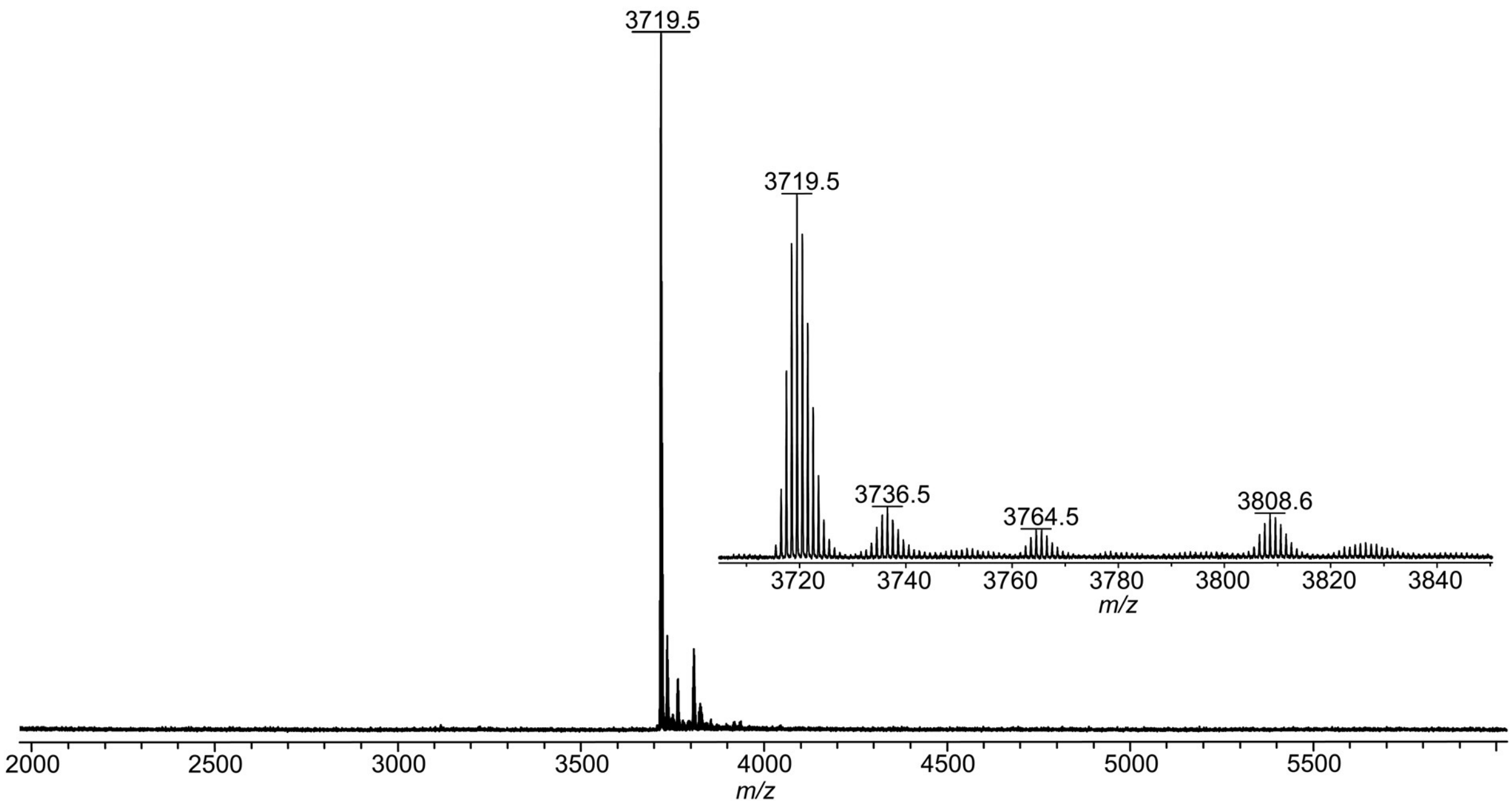

Figure S48 MALDI-TOF mass spectrum of **1** (matrix: DCTB).

8.2 Synthesis of 2

Figure S49 Reagents and conditions: a) $Pd(PPh_3)_4$, Cs_2CO_3, H_2O:EtOH:toluene (9:1:20), reflux, 42 h, 96 %; b) $BF_3{\bullet}OEt_2$, 1,2-dichlorethane, 80 °C, 3 h, 13 %; c) benzoic anhydride, 150 °C, 4 h, 46 %; d) $PdCl_2(PPh_3)_2$, PPh_3, CuI, TMS-acetylene, THF:triethylamine (1:2), r.t., 18 h, 96 %; e) K_2CO_3, MeOH:THF (1:2), r.t., 18 h, 97 %; f) **18**, $PdCl_2(PPh_3)_2$, PPh_3, CuI, THF:piperidine (1:2), r.t., 18 h, 92 %; g) $Co_2(CO)_8$, toluene, reflux, 18 h, 24 %; h) $Ni(COD)_2$, bipyridine, THF, COD, microwave, 120 °C, 12 min, 27 %.

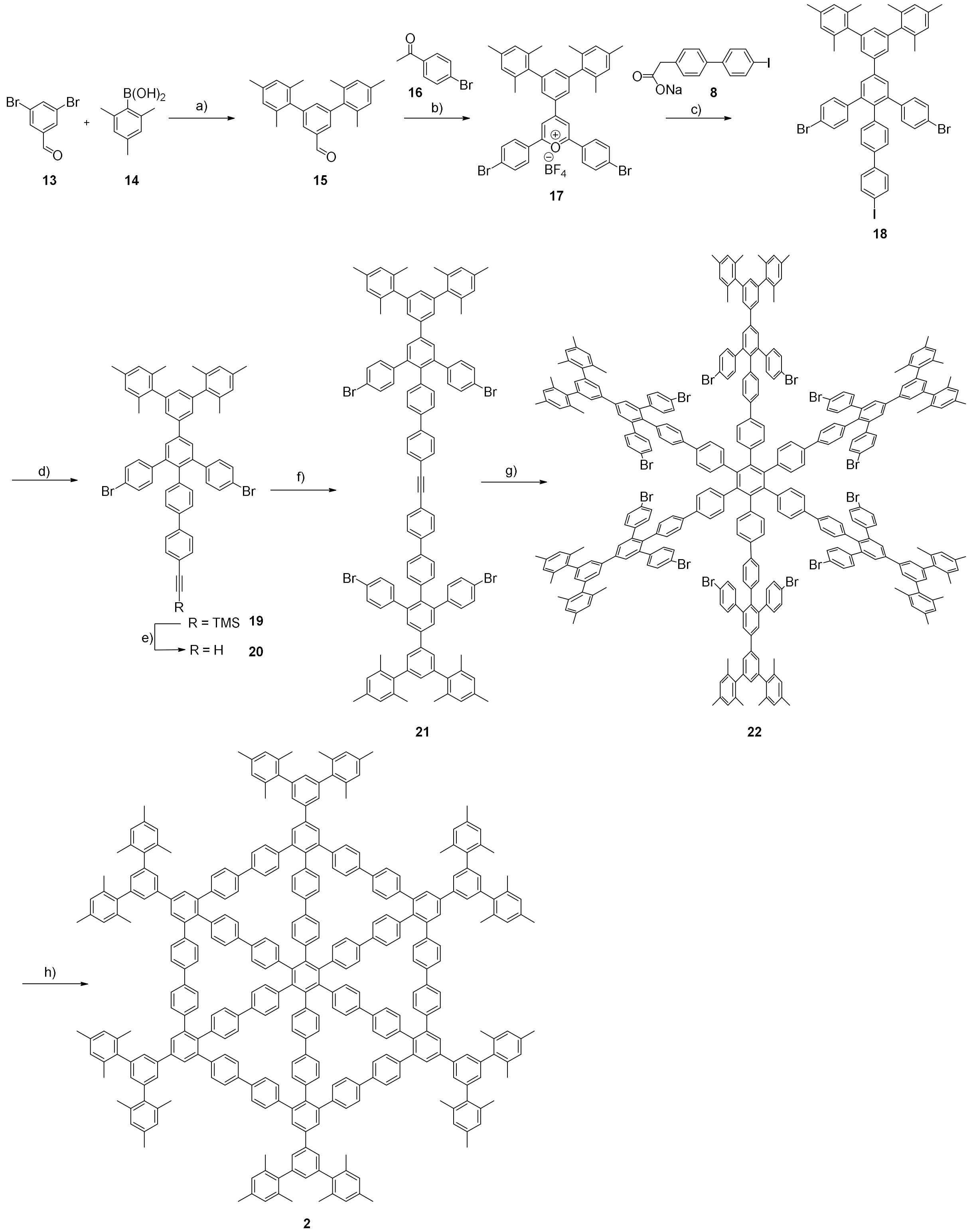

15

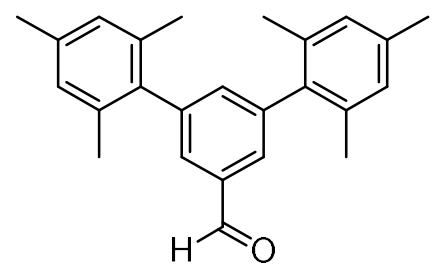

New:

Water/EtOH(9:1; 100 mL) an toluene (200 mL) were saturated with Ar and added to an Ar flushed mixture of **13** (6.70 g, 25.4 mmol), **14** (10.00 g, 61.00 mmol) and Cs_2CO3. After addition of $Pd(PPh_3)_4$ (2.35 g, 2.03 mmol) the mixture was heated under reflux for 42 h. After cooling to r.t., the reaction was diluted with aq HCl (10 %) and DCM and the aqueous layer was extracted with DCM. The combined organic phases were washed with water, HCl (10 %), water and NaOH (10 %) and brine, and dried over $MgSO_4$. The crude product was purified by column chromatography (CH:DCM = 1:1; R_f = 0.37) and **15** (8.37 g, 24.4 mmol, 96 %) was obtained as a white solid.

Chemical formula: $C_{25}H_{26}O$. **^{1}H NMR** (500 MHz, $CDCl_3$, r.t.): δ [ppm] = 10.09 (s, 1H), 7.66 (d, J = 1.7 Hz, 2H), 7.24 (t, J = 1.6 Hz, 1H), 6.96 (s, 4H), 2.34 (s, 6H), 2.05 (s, 12H). **^{13}C NMR** (126 MHz, $CDCl_3$, r.t.): δ [ppm] = 192.6, 142.5, 137.5, 137.3, 137.1, 136.9, 135.7, 129.2, 128.3, 27.1, 20.9. **MS** (EI, 70 eV) m/z (%) = 342.2 (90) $[M]^+$, 313.2 (23) $[M\text{-}CO]^+$, 224.1 (100) $[M\text{-}C_9H_{10}]^+$, 195.1 (37) $[M\text{-}C_9H_{10}CHO]^+$.

17

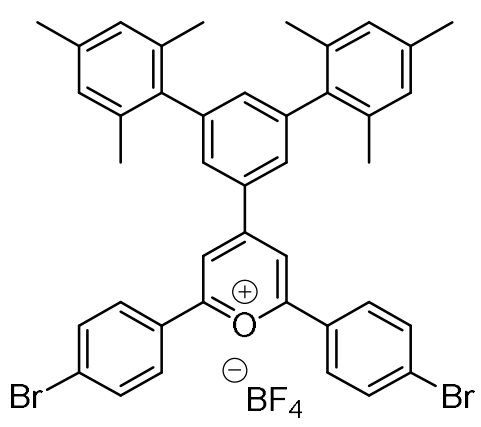

Under an Ar Atmosphere, **15** (3.45 g, 10.1 mmol) and **16** (4.02 g, 20.2 mmol) were dissolved in 1,2-dichlorethane (9 mL). Boron trifluoride diethyl etherate (3.30 mL, 25.3 mmol) was added and the reaction mixture was stirred at 80 °C for 3 h. After cooling to r.t., the solution was diluted with DCM and the product precipitated in diethylether (150 mL). The precipitate was filtered off and washed with diethyl ether. **17** (1.00 g, 1.26 mmol, 13 %) was obtained as a yellow solid.

Chemical formula: $C_{41}H_{35}BBr_2F_4O$. **^{1}H NMR** (700 MHz, DMSO-d_6, r.t.): δ [ppm] = 9.25 (s, 2H), 8.53–8.51 (m, 6H), 7.99–7.96 (m, 4H), 7.35 (s, 1H), 7.03 (s, 4H), 2.31 (s, 6H), 2.06 (s, 12 H). **^{13}C NMR** (176 MHz, DMSO-d_6, r.t.): δ [ppm] = 169.3, 166.1, 164.7, 142.7, 137.1, 136.6, 135.2, 132.7, 130.7, 129.5, 129.2, 128.3, 128.1, 115.4, 20.5, 20.4. **MS** (ESI+) m/z (%) = 703.1 (100) $[M]^{+\bullet}$, 585.0 (9) $[M\text{-}Br_2\text{-}C_3H_9]^+$.

18

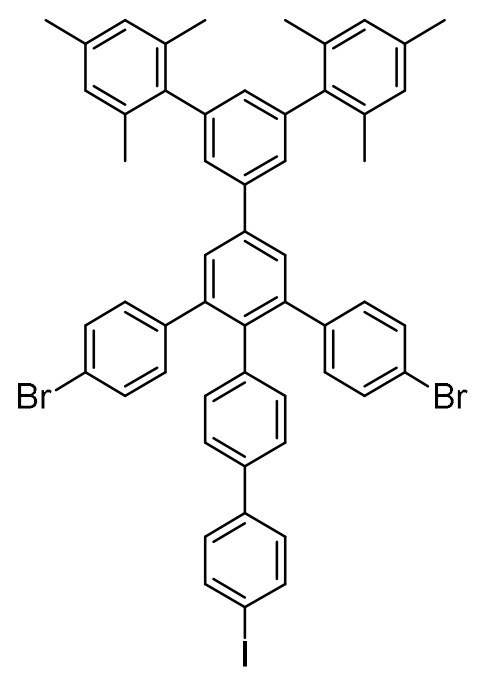

Benzoic anhydride (10.0 g, 44.2 mmol), **17** (1.00 g, 1.26 mmol) and **8** (0.91 g, 2.54 mmol) were purged with Ar and stirred at 150 °C for 4 h. After cooling the mixture to r.t., it was dissolved in DCM:CH (1:1) and purified by column chromatography (CH:DCM = 1:1; R_f = 0.90). **18** (0.57 g, 0.58 mmol, 46 %) as a white solid.

Chemical formula: $C_{54}H_{43}Br_2I$. **^{1}H NMR** (500 MHz, $CDCl_3$, r.t.): δ [ppm] = 7.72 (d, J = 8.4 Hz, 2H), 7.68 (s, 2H), 7.49 (d, J = 1.54 Hz, 2H) 7.33–7.26 (m, 8H), 7.00 (d, J = 8.5 Hz, 4H), 6.97 (s, 5H), 6.90 (d, J = 8.4 Hz, 2H), 2.35 (s, 6H), 2.10 (s, 12H). **^{13}C NMR** (126 MHz, $CDCl_3$, r.t.): δ [ppm] = 142.2, 141.5, 140.6, 140.3, 140.1, 139.9, 138.9, 138.2, 137.8, 137.7, 137.4, 136.9, 136.0, 132.2, 131.6, 131.1, 129.9, 128.9, 128.6, 128.3, 126.2, 126.1, 121.0, 93.1, 21.2, 20.9. **MS** (MALDI pos) m/z (%) = 978.0 (100) $[M]^+$.

19

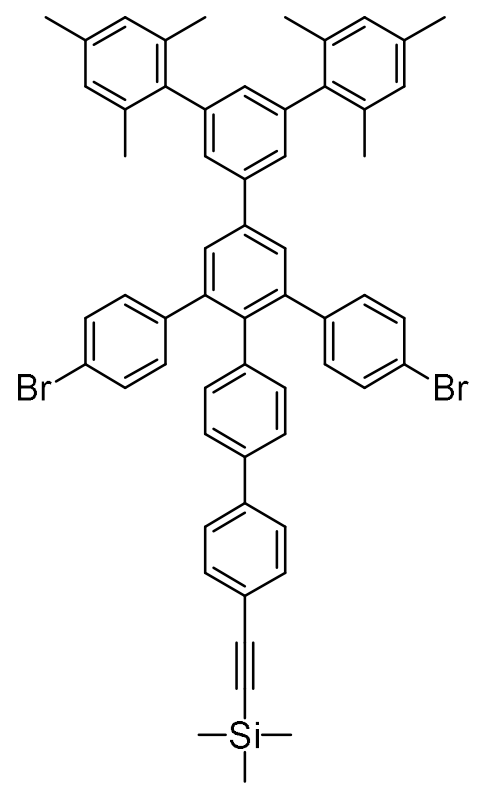

18 (395 mg, 0.40 mmol), $PdCl_2(PPh_3)_2$ (17.0 mg, 24.2 µmol), PPh_3 (12.7 mg, 48.4 µmol) and CuI (160 mg, 0.84 mmol) were dissolved in THF (10 mL) and triethylamine (20 mL) in a dry Schlenk tube and purged with Ar for 1 h. TMS-acetylene (0.26 mL, 1.80 mmol) was added slowly to the solution, which was then stirred at r.t. for 18 h. The solution was diluted with DCM and HCl (10 %) and the aqueous phase was extracted three times with DCM. The combined organic phases were washed with water and brine and dried over $MgSO_4$. The crude product was purified by column chromatography (CH:DCM = 2:1; R_f = 0.78). **19** (368 mg, 0.39 mmol, 96 %) was obtained as yellow solid.

Chemical formula: $C_{59}H_{52}Br_2Si$. **^{1}H NMR** (500 MHz, CD_2Cl_2, r.t.): δ [ppm] = 7.71 (s, 2H), 7.53–7.45 (m, 6H), 7.35–7.29 (m, 6H), 7.07–7.02 (m, 4H), 6.97–6.92 (m, 7H), 2.31 (s, 6H), 2.09 (s, 12H), 0.25 (s, 9H). **^{13}C NMR** (126 MHz, CD_2Cl_2, r.t.): δ [ppm] = 142.7, 142.0, 141.4, 140.6, 139.3, 138.8, 138.2, 138.0, 137.3,

136.3, 132.8, 132.6, 132.1, 131.4, 130.4, 128.8, 128.6, 127.1, 126.6, 126.4, 122.6, 121.2, 105.3, 95.5, 88.4, 86.6, 21.2, 21.1, 0.3. **MS** (MALDI pos) *m*/*z* (%) = 948.2 (100) $[M]^{+\bullet}$.

20

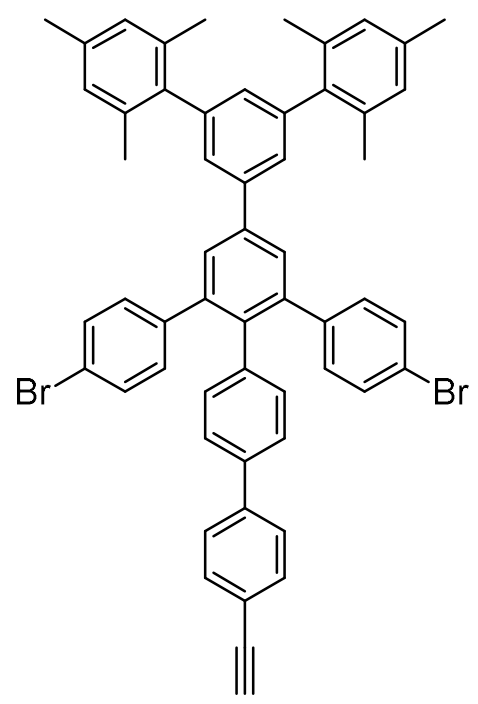

Under an Ar atmosphere, **19** (348 mg, 0.37 mmol) and K_2CO_3 (508 mg, 3.67 mmol) were dissolved in THF (18 mL) and MeOH (9 mL) in a dry Schlenk tube and the reaction mixture was stirred for at r.t. 18 h. The reaction was diluted with water and DCM and the aqueous phase was extracted three times with DCM. The combined organic phases were washed with water and dried over $MgSO_4$. The crude product was purified with column chromatography (CH:DCM = 2:1; R_f = 0.69). **20** (274 mg, 0.31 mmol, 97 %) was obtained as a white solid.

Chemical formula: $C_{56}H_{44}Br_2$. **^{1}H NMR** (500 MHz, CD_2Cl_2, r.t.): *δ* [ppm] = 7.71 (s, 2H), 7.54–7.49 (m, 6H), 7.35–7.29 (m, 6H), 7.07–7.03 (m, 4H), 6.98–6.91 (m, 7H), 3.18 (s, 1H), 2.31 (s, 6H), 2.09 (s, 12H). **^{13}C NMR** (126 MHz, CD_2Cl_2, r.t.): *δ* [ppm] = 142.7, 142.0, 141.3, 141.2, 140.7, 140.5, 139.3, 139.0, 138.3, 138.0, 137.3, 136.3, 133.0, 132.6, 132.1, 131.4, 130.4, 128.8, 128.6, 127.1, 126.6, 126.4, 121.5, 121.2, 95.5, 83.8, 78.3, 27.5, 21.1. **MS** (MALDI pos) *m*/*z* (%) = 876.2 (100) $[M]^+$.

21

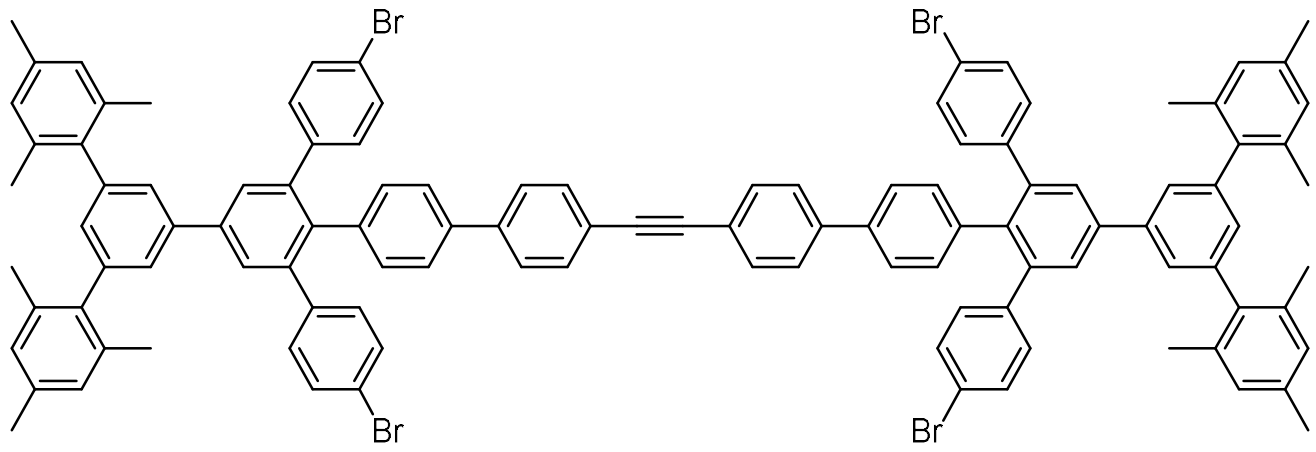

20 (264 mg, 0.27 mmol) and PPh_3 (14.3 mg, 0.05 mmol) were dissolved in a dry Schlenk tube in THF (12 mL) and piperidine (20 mL) and purged with Ar for 1 h. CuI (102 mg, 0.54 mmol), $PdCl_2(PPh_3)_2$ (10.8 mg, 0.02 mmol) and **18** (281 mg, 0.32 mmol) were added and the reaction mixture was stirred at r.t. for 18 h. The reaction was diluted with aq HCl (10 %) and DCM. The aqueous layer was extracted with DCM and the combined organic phases were washed with water and brine, and dried over $MgSO_4$. The crude product was dissolved in DCM and purified by precipitation in methanol. **21** (428 mg, 0.25 mmol, 92 %) was obtained as a yellow solid.

Chemical formula: $C_{110}H_{86}Br_4$. **^{1}H NMR** (500 MHz, CD_2Cl_2, r.t.): δ [ppm] = 7.72 (s, 4H), 7.57 (s, 8H), 7.50 (d, J = 1.56, 4H), 7.38–7.30 (m, 12H), 7.07–7.04 (m, 8H), 6.98–6.92 (m, 14H), 2.32 (s, 12H), 2.09 (s, 24H). **^{13}C NMR** (126 MHz, CD_2Cl_2, r.t.): δ [ppm] = 142.7, 142.0, 141.3, 140.7, 140.6, 140.5, 139.3, 138.8, 138.4, 138.0, 137.3, 136.3, 132.6, 132.5, 132.1, 131.4, 130.4, 128.9, 128.6, 127.3, 126.6, 126.4, 122.7, 121.1, 90.6, 21.2, 21.2. **MS** (MALDI pos) m/z (%) = 1727.4 (97) $[M]^+$, 1611.3 (4) $[M\text{-}Mes]^+$.

22

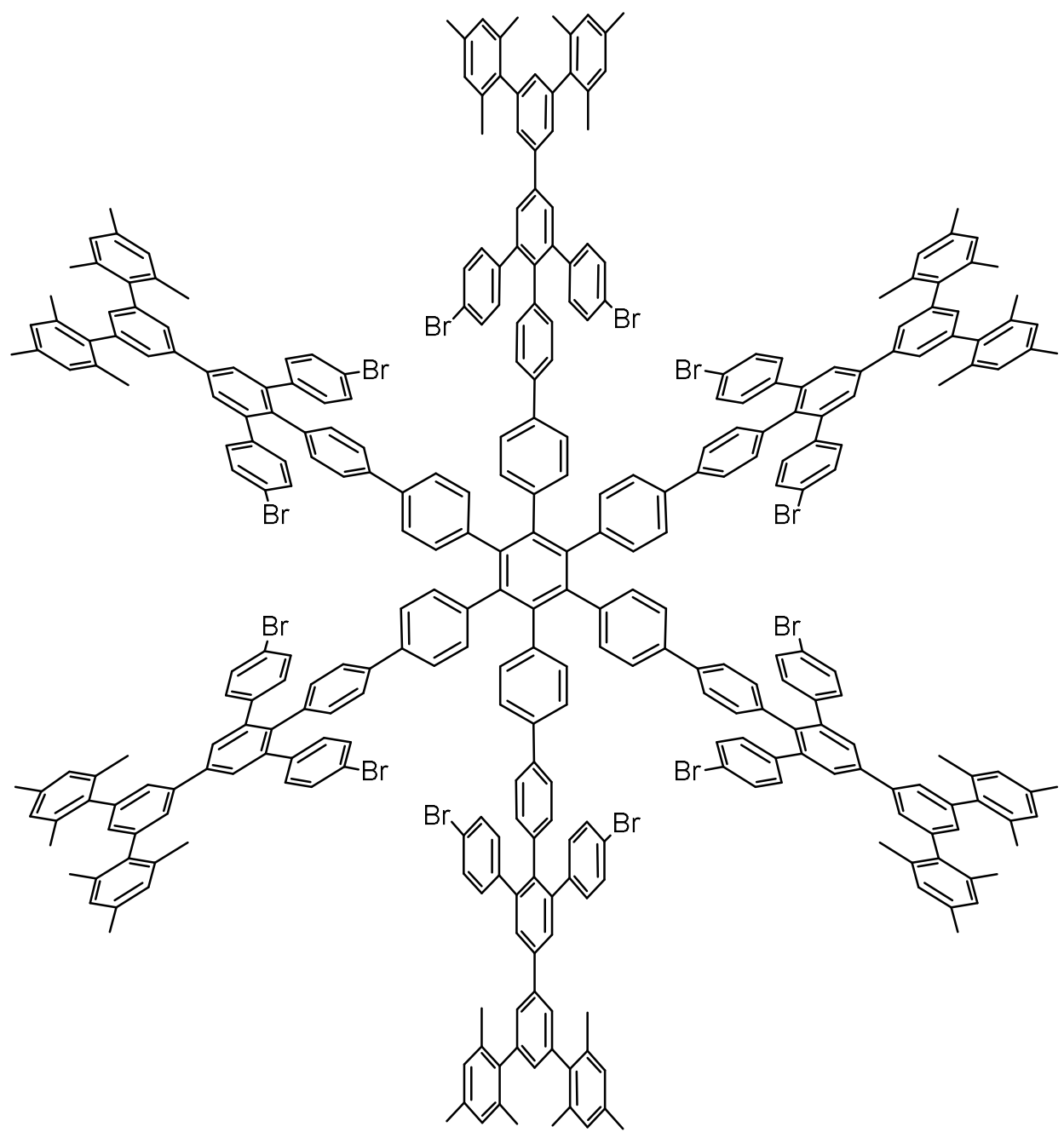

21 (397 mg, 229 µmol) was placed in a dry Schlenk tube and then toluene (15 mL), which was purged with Ar, and $Co_2(CO)_8$ (15.7 mg, 45.9 µmol) were added. The mixture was stirred under reflux for 18 h. After cooling to r.t., the reaction was diluted in water and DCM and the aqueous layer was extracted with DCM. The combined organic phases were washed with water, aq HCl (10 %) and brine., and was dried over $MgSO_4$. The crude product was purified by column chromatography (DCM) and recGPC, and **22** (85.5 mg, 16.5 µmol, 24 %) was obtained as white solid.

Chemical formula: $C_{330}H_{258}Br_{12}$. **^{1}H NMR** (700 MHz, CD_2Cl_2, r.t.): δ [ppm] = 7.65 (s, 12H), 7.45 (s, 12H), 7.24 (d, J = 8.1 Hz, 24H), 7.18–7.07 (m, 24H) 6.99 (d, J = 8.3 Hz, 24H), 6.97–6.88 (m, 42H), 6.82 (d, J = 8.3 Hz, 12H), 2.31 (s, 36H), 2.06 (s, 72H). **^{13}C NMR** (176 MHz, CD_2Cl_2, r.t.): δ [ppm] = 142.5, 142.0, 141.3, 140.6, 140.5, 140.4, 140.4, 139.2, 138.6, 138.6, 138.1, 138.1, 137.1, 136.2, 132.5, 132.3, 132.2, 131.4, 130.2, 128.8, 128.4, 126.7, 126.1, 125.5, 121.2, 21.2, 21.2. **MS** (MALDI pos) m/z (%) = 5285.1 (4) $[M+Ag]^+$, 5207.5 (16) $[M+Na]^+$, 5182.3 (100) $[M]^+$, 5101.9 (6) $[M-Mes+K]^+$. **GPC** (THF, *vs*. PS) $M_p = 4.4\cdot10^3$ g/mol.

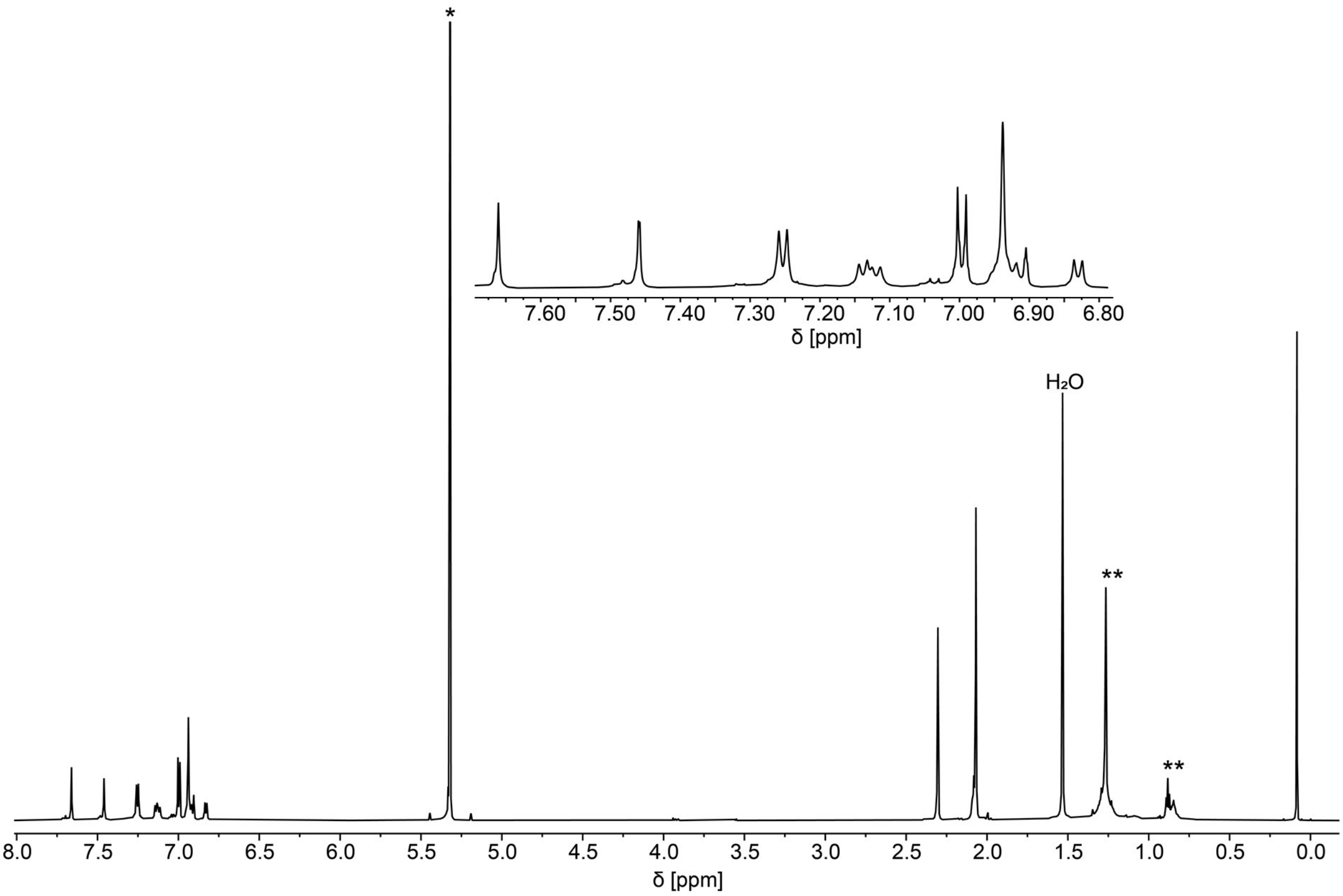

Figure S50 ^{1}H NMR spectra of **22** in CD_2Cl_2* (**H grease impurities).

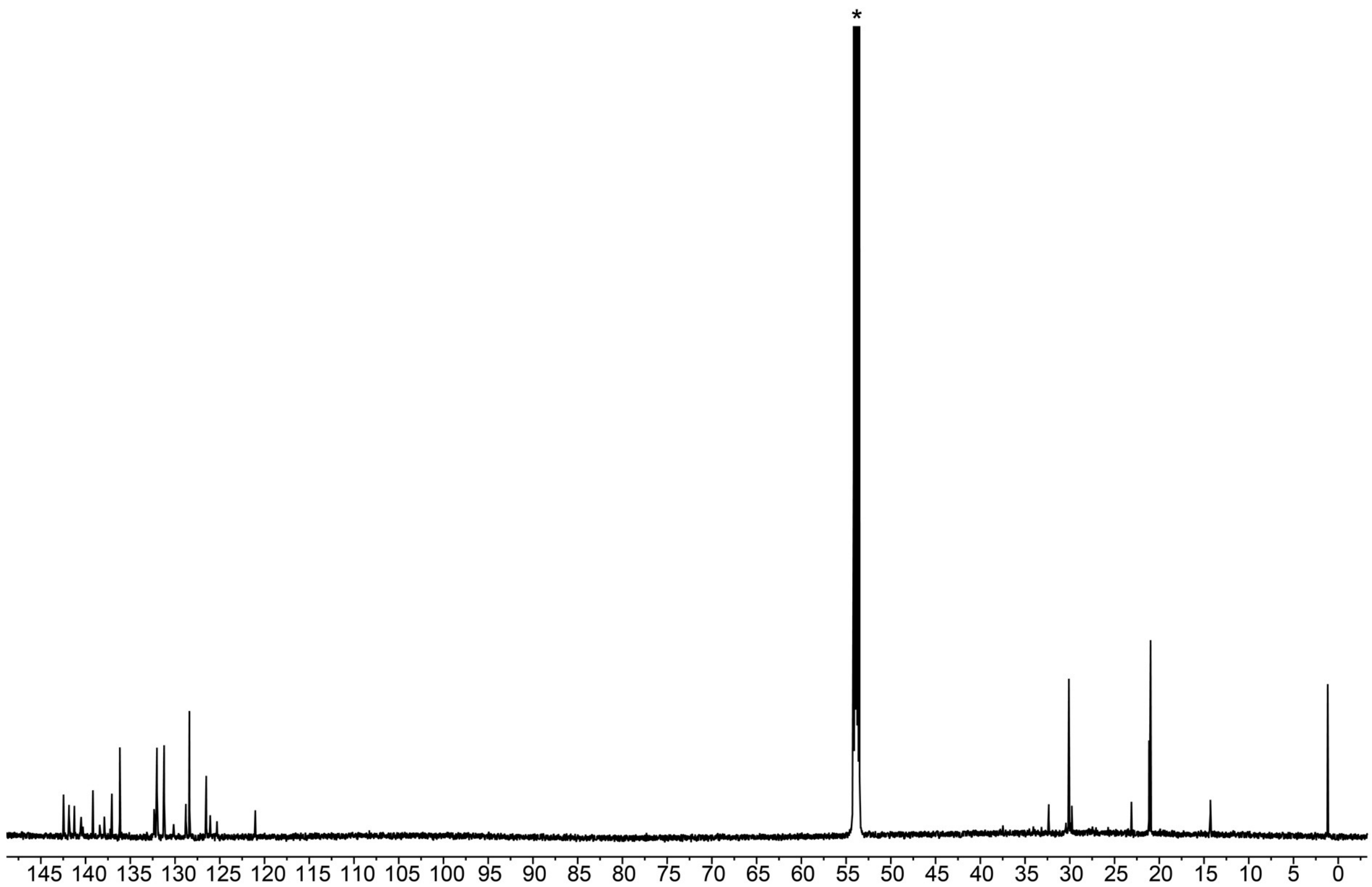

Figure S51 ^{13}C NMR spectra of **22** in CD_2Cl_2*.

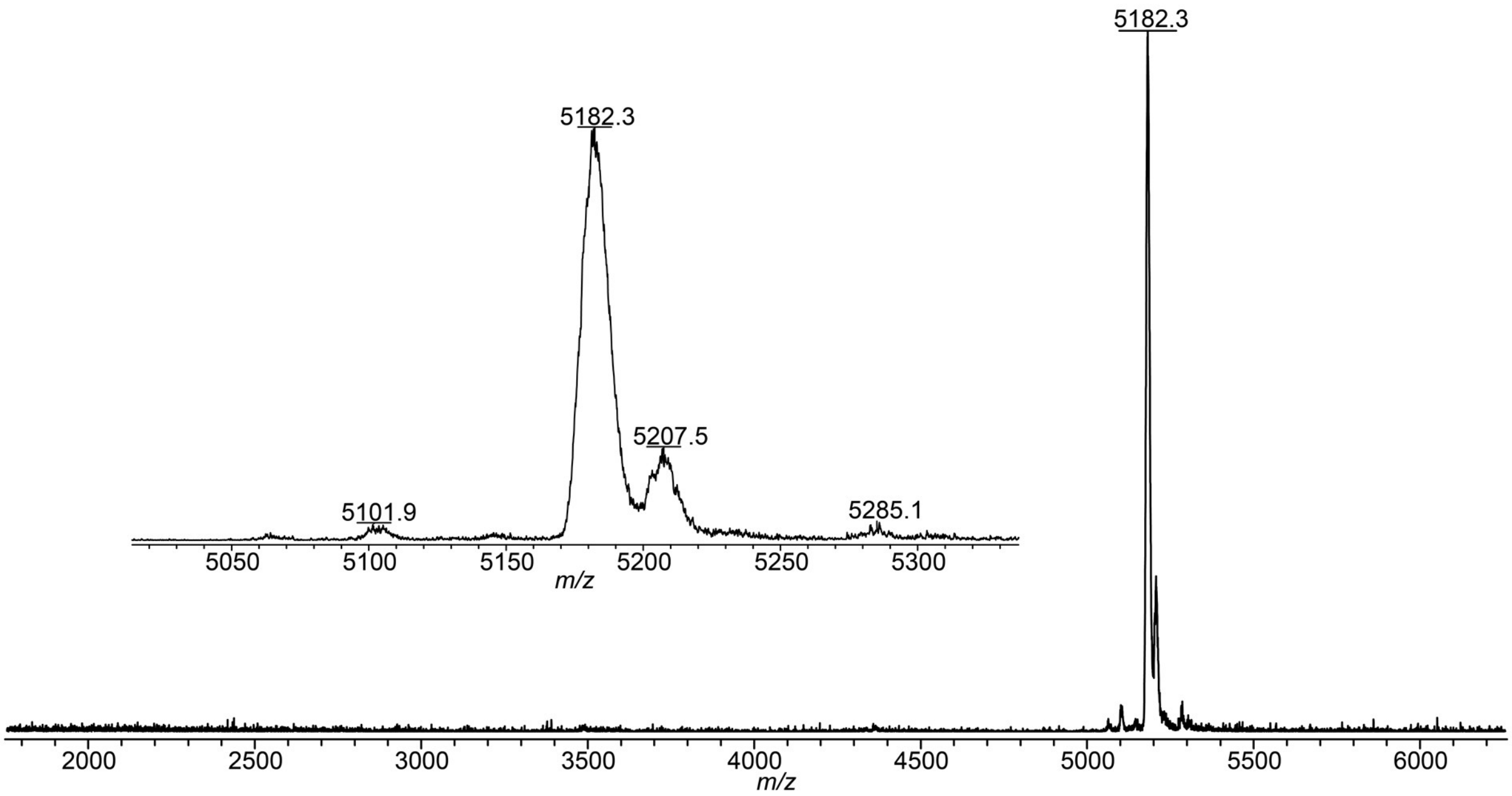

Figure S52 MALDI-TOF mass spectrum of **22** (matrix: DCTB).

2

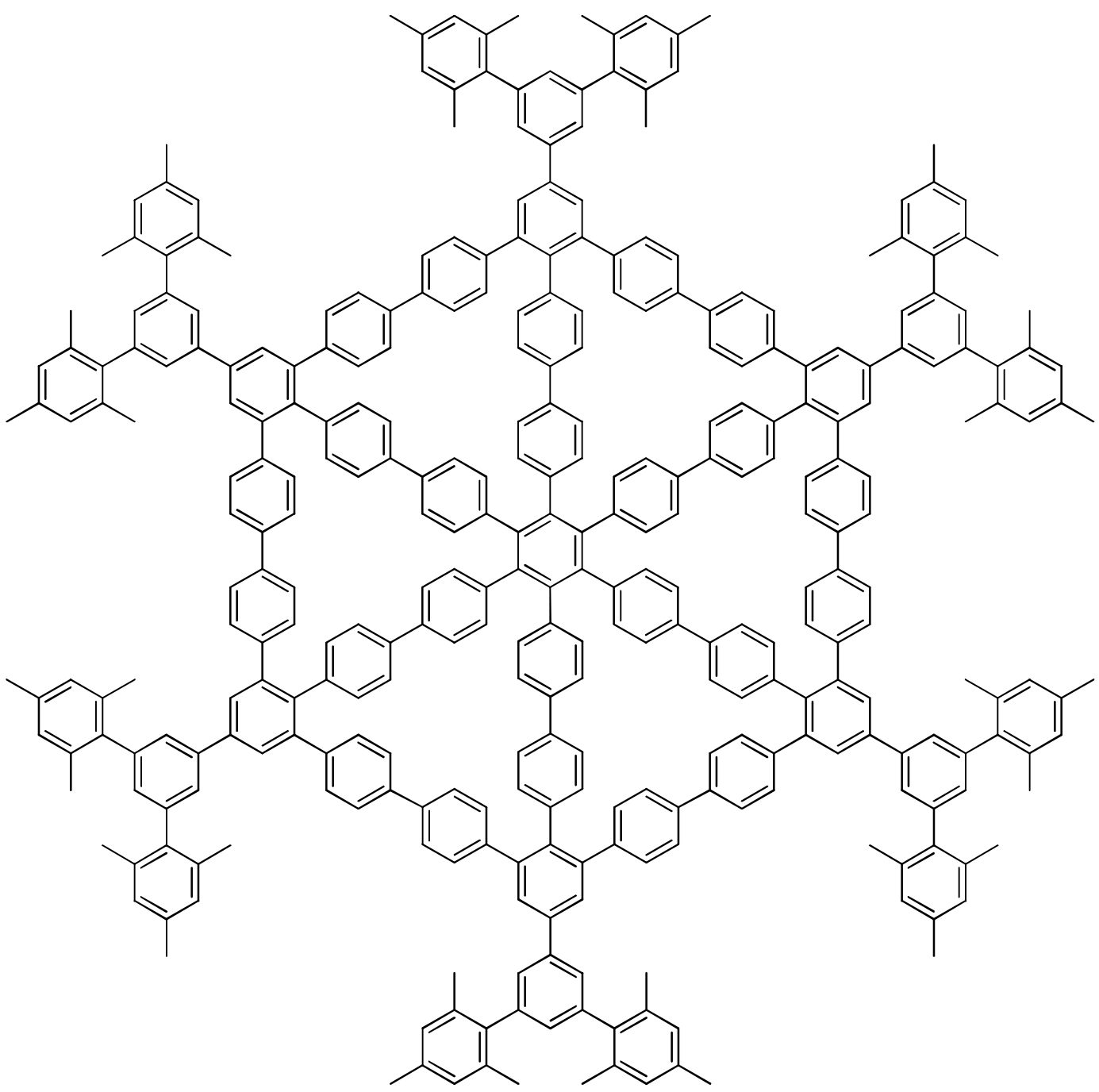

Under an Ar atmosphere, **22** (10.7 mg, 2.05 µmol), Ni(COD)$_2$ (10.0 mg, 36.4 µmol) and bipyridine (5.00 mg, 32.0 µmol) were dissolved in THF (8 mL) and COD (0.25 mL) in a microwave tube. The reaction mixture was stirred in a microwave at 120 °C for 12 min and afterwards the solvent was removed under reduced pressure. The crude product was purified by column chromatography (DCM) and recrystallized from 1,1,2,2-tetrachloroethane. Removal of the solvent gave **2** (2.31 mg, 0.547 µmol, 27 %) as white solid.

Chemical formula: $C_{330}H_{258}$. **^{1}H NMR** (700 MHz, CD_2Cl_2, r.t.): δ [ppm] = 7.75 (s, 12H), 7.50 (d, J = 1.4 Hz, 12H), 7.43 (d, J = 8.1 Hz, 24H), 7.18–7.08 (m, 48H), 6.93 (s, 24H), 6.92–6.89 (m, 18H), 6.81 (d, J = 8.3 Hz, 12H), 2.30 (s, 36H), 2.09 (s, 72H). **^{13}C NMR** (176 MHz, CD_2Cl_2, r.t.): δ [ppm] = 142.6, 142.6, 141.8, 141.4, 140.1, 139.3, 139.0, 137.8, 137.2, 136.2, 132.3, 132.0, 130.7, 130.7, 130.3, 130.2, 130.0, 129.0, 128.4, 128.1, 127.9, 126.7, 126.6, 126.3, 125.9, 21.2, 21.1. **MS** (MALDI pos) m/z (%) = 4223.3 (100) $[M]^{+\bullet}$, 4105.0 (22) $[M\text{-}Mes]^{+}$. **GPC** (THF, *vs*. PS) $M_p = 3.8 \cdot 10^3$ g/mol.

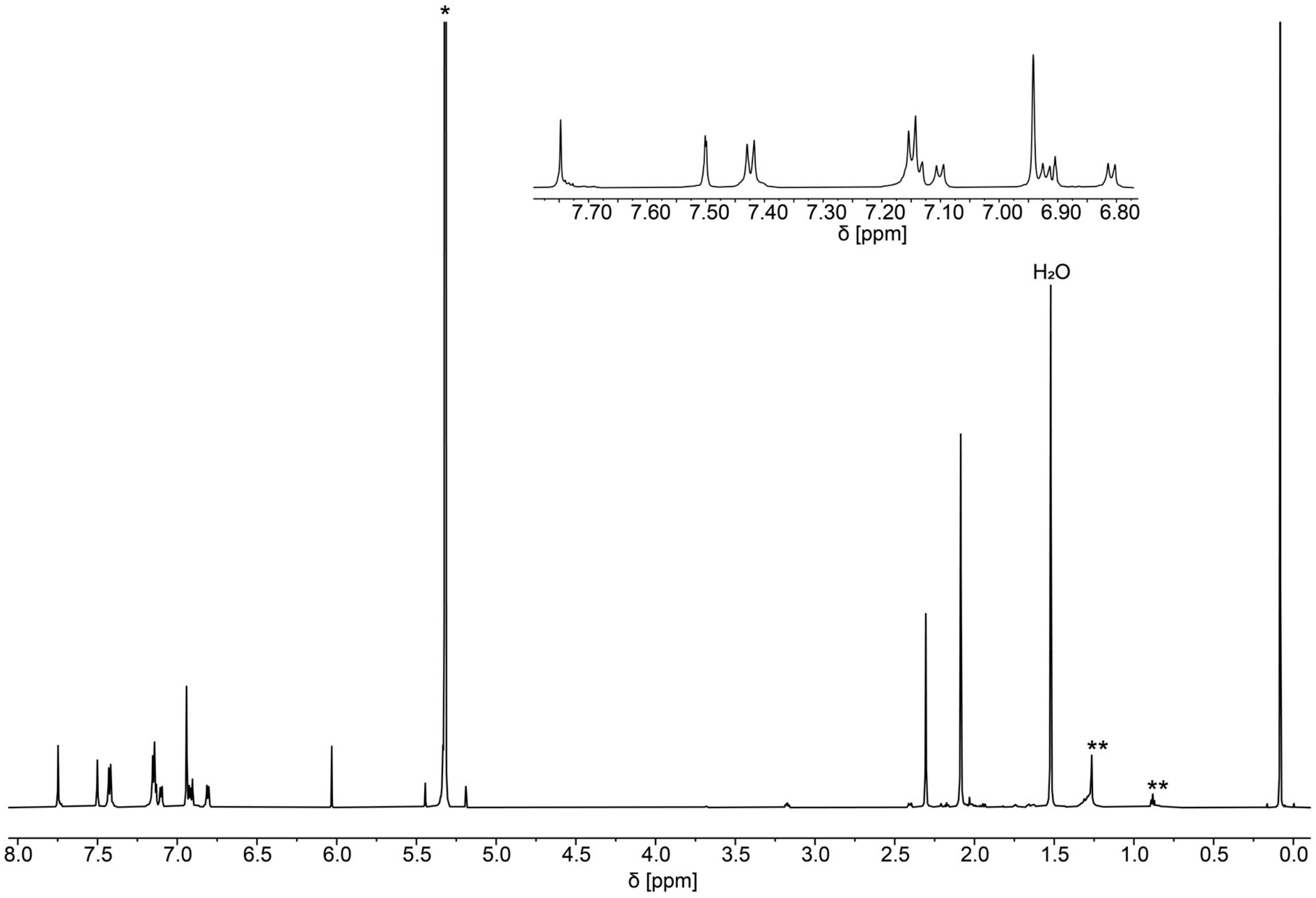

Figure S53 ^{1}H NMR spectra of **2** in CD_2Cl_2* (**H grease impurities).

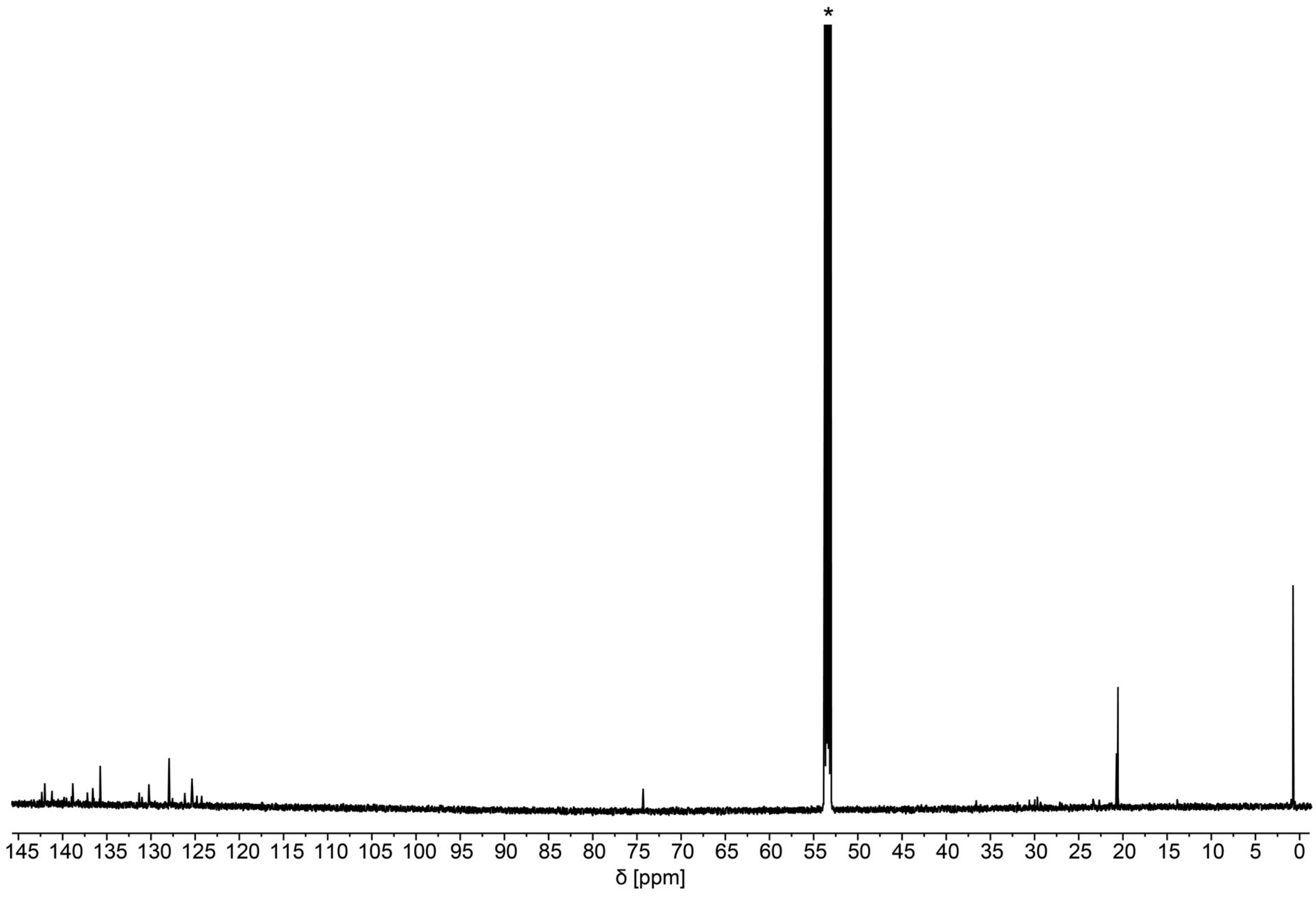

Figure S54 ^{13}C NMR spectra of **2** in CD_2Cl_2*.

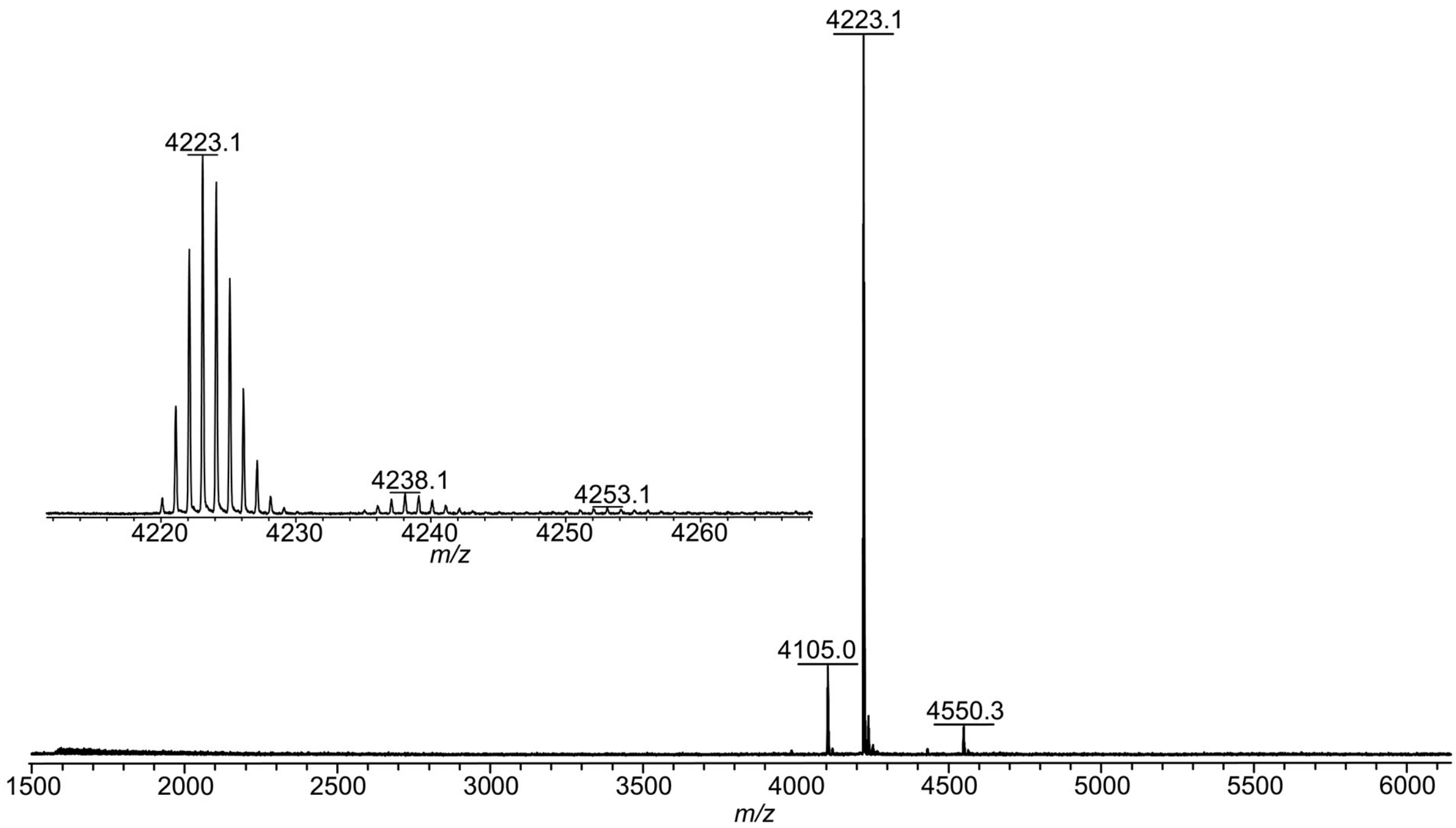

Figure S55 MALDI-TOF mass spectrum of **2** (matrix: DCTB).

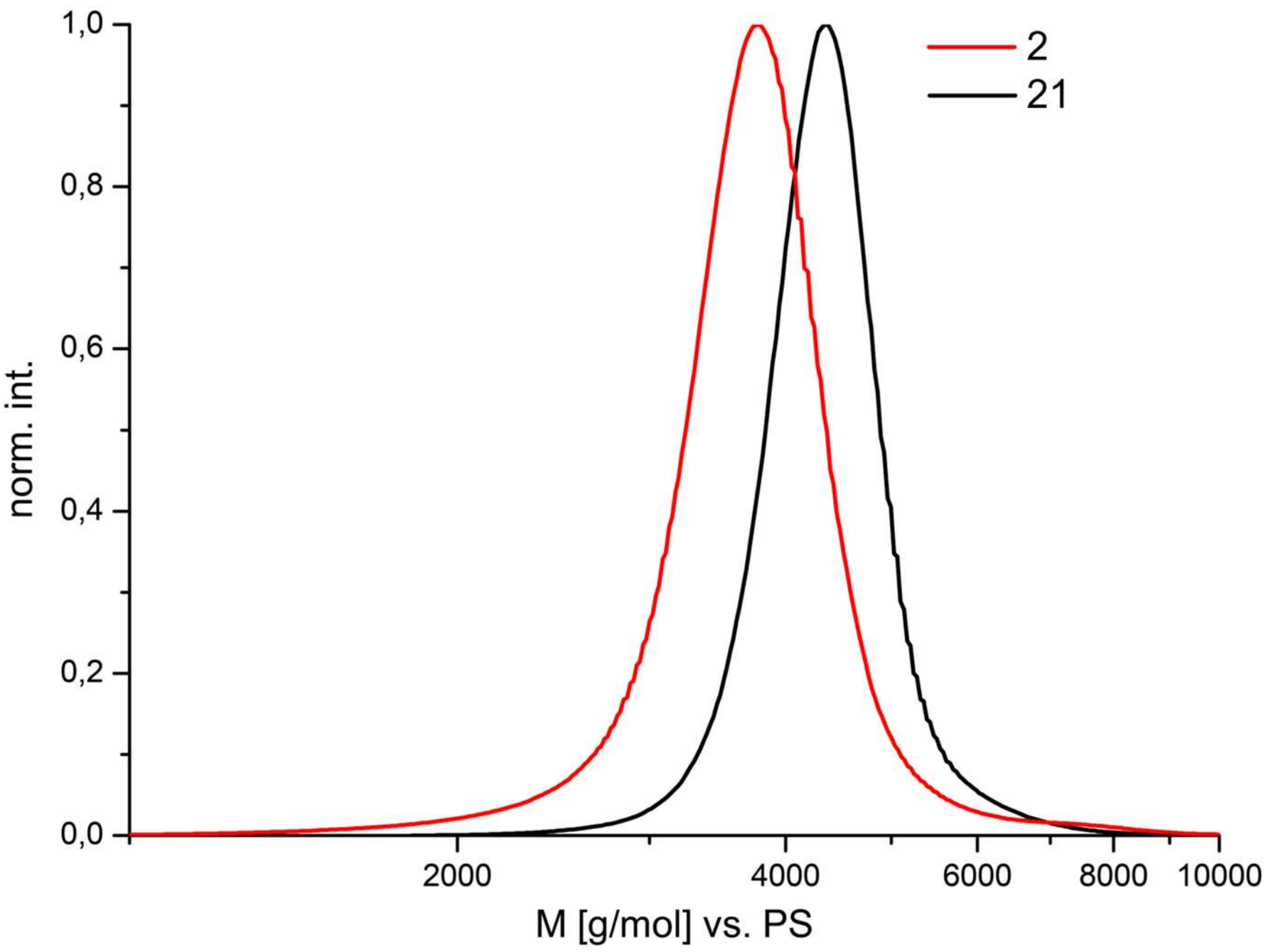

Figure S56 Molar mass distribution of the GPC analyses of **22** (black; M_p = 4371 g/mol) and **2** (red; M_p = 3775 g/mol).

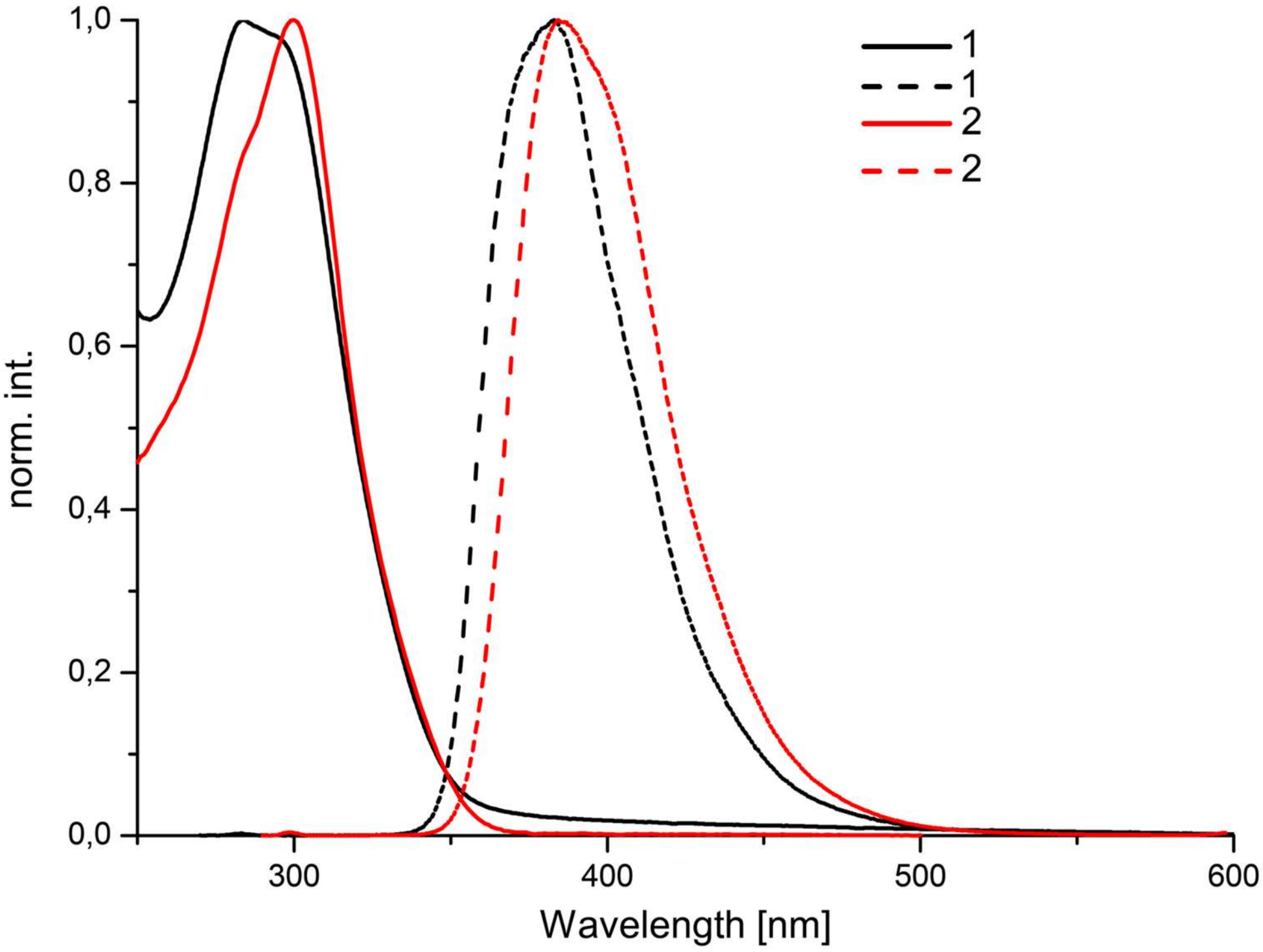

Figure S57 Normalized UV/vis spectra (solid) and fluorescence spectra (dashed) of **1** (black; maxima at UV/vis: 283.8 nm, F: 383 nm) and **2** (red; maxima at UV/vis: 300 nm, F: 384.5 nm) in $CHCl_3$ at r.t. in air.

8.3 Synthesis of 3

Figure S58 Reagents and conditions: a) BF_3•OEt_2, 1,2-dichlorethane, 80 °C, 3 h, 26 %; b) benzoic anhydride, 150 °C, 4 h, 26 %; c) BBr_3, DCM, -78 °C to r.t., 18 h; 75 %; d) 3-(boc-amino)-propyl bromide, KI, Cs_2CO_3, acetone, reflux, 18 h, 95 %; e) $PdCl_2(PPh_3)_2$, PPh_3, CuI, TMS-acetylene, THF:piperidine (1:2), r.t., 18 h, 94 %; f) K_2CO_3, MeOH:THF (1:2), r.t., 18 h, 94 %; g) BF_3•OEt_2, 1,2-dichlorethane, 80 °C, 3 h, 11 %; h) benzoic anhydride, 150 °C, 4 h, 13 %; i) BBr_3, DCM, -78 °C to r.t., 18 h; 71 %; j) hexadecyl bromide, KI, Cs_2CO_3, acetone, reflux, 18 h, 99 %;

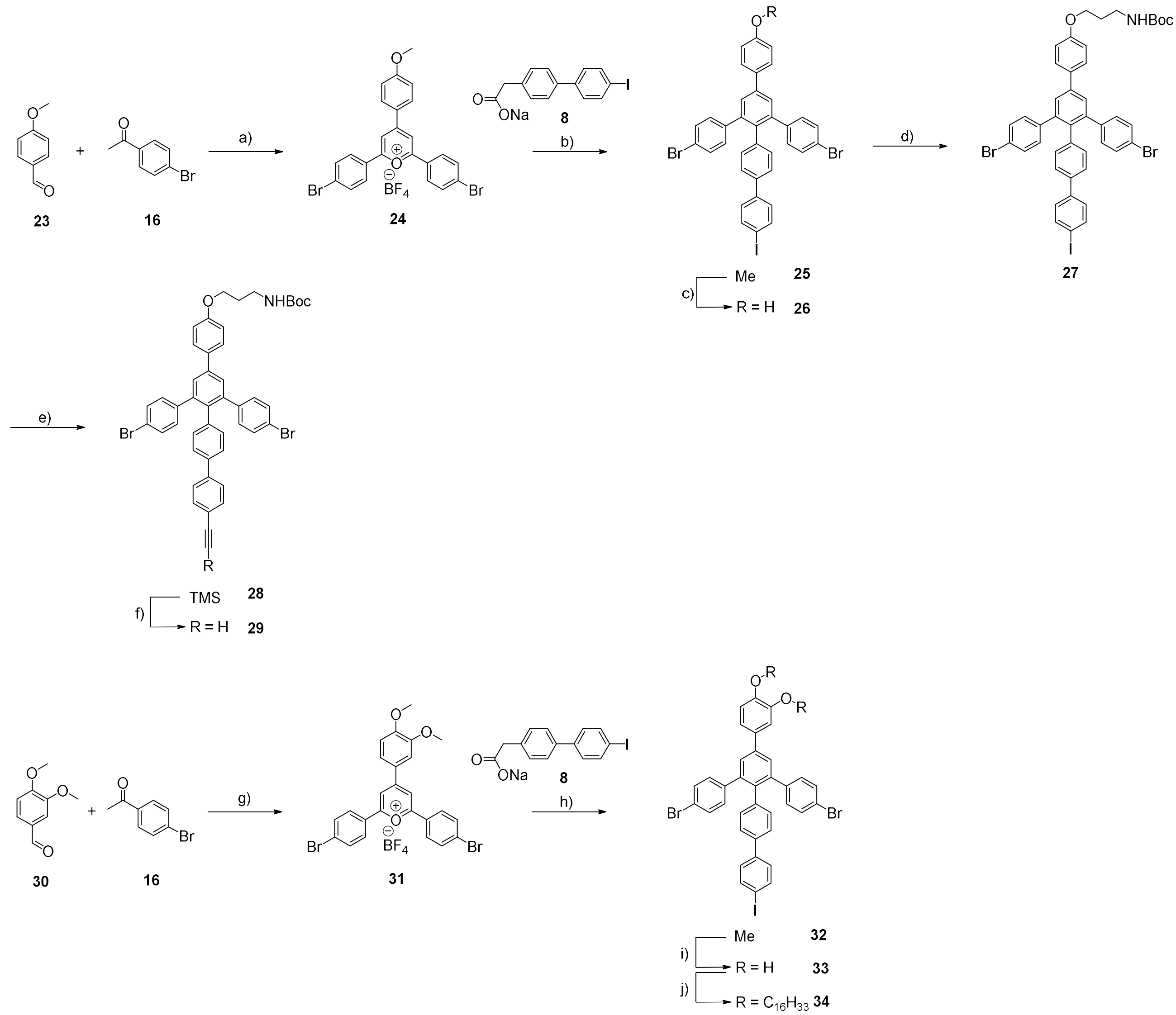

Figure S59 Reagents and conditions: a) $PdCl_2(PPh_3)_2$, PPh_3, CuI, THF:piperidine (1:2), r.t., 18 h, 75 %; b) $Co_2(CO)_8$, toluene, reflux, 18 h, 10 %; h) $Ni(COD)_2$, bipyridine, THF, COD, microwave, 120 °C, 12 min, 64 %.

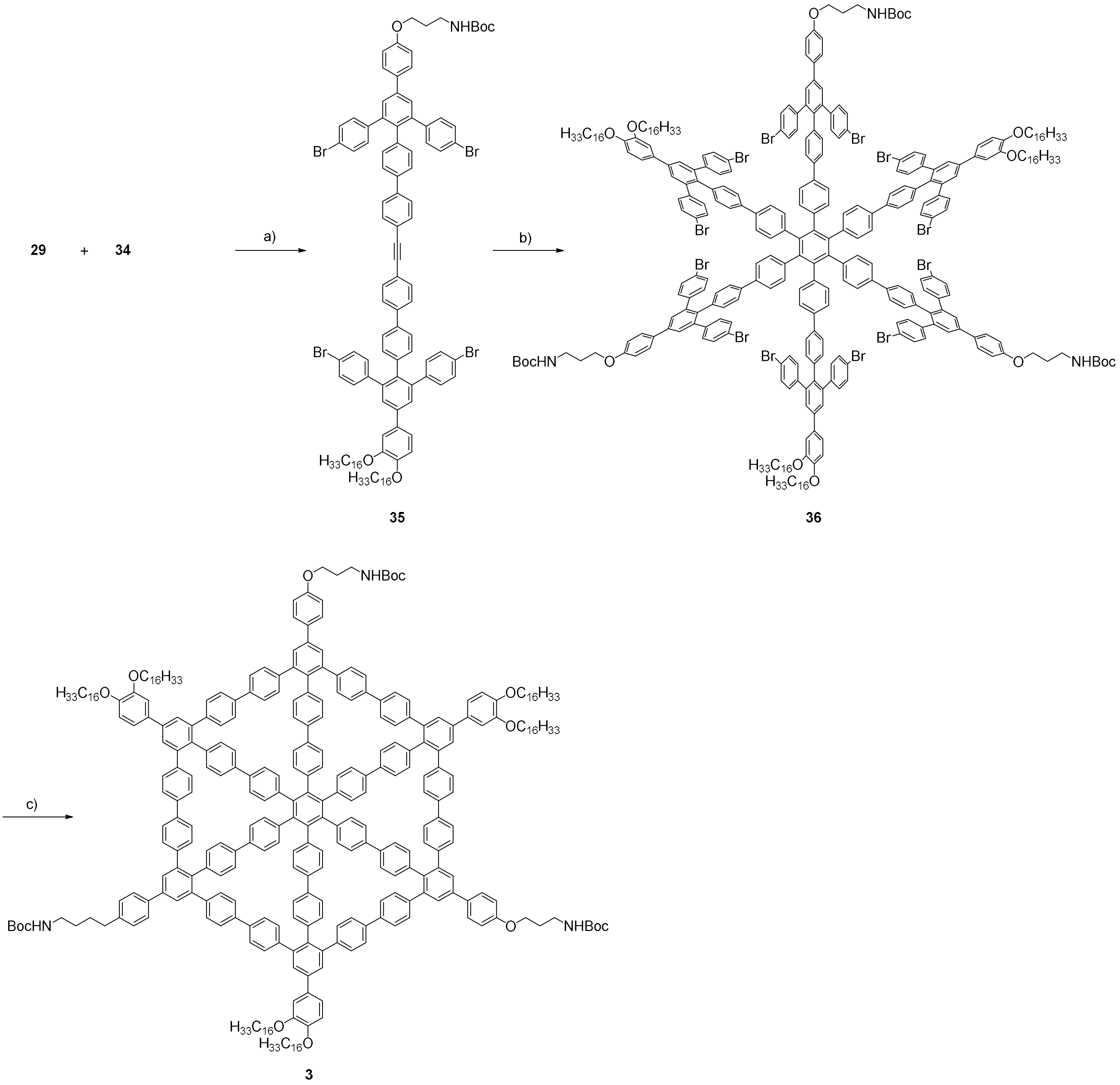

24

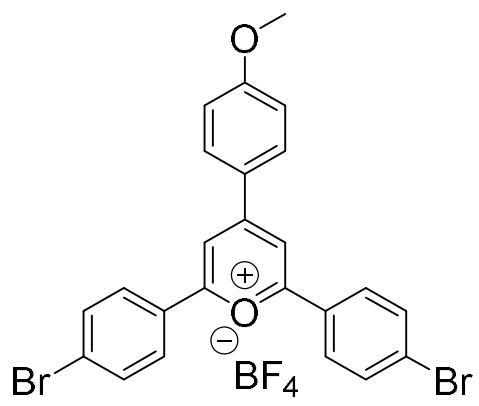

Under an Ar atmosphere, **23** (10.0 g, 73.5 mmol) and **16** (29.2 g, 147 mmol) were dissolved in 1,2-dichlorethane (25 mL) in a dry Schlenk tube. Boron trifluoride diethyl etherate (23.3 mL, 184 mmol) was added and the reaction mixture was stirred at 80 °C for 3 h. After cooling to r.t., the solution was diluted with DCM and the crude product precipitated in ethylacetate (200 mL). The precipitate was filtered off and washed with ethylacetate. **24** (11.1 g, 19.0 mmol, 26 %) was obtained as an orange solid.

Chemical formula: $C_{24}H_{17}BBr_2F_4O_2$. **^{1}H NMR** (500 MHz, DMSO-*d*$_6$, r.t.): *δ* [ppm] = 9.06 (s, 2H), 8.70 (d, *J* = 9.1 Hz, 2H), 8.46 (d, *J* = 8.8 Hz, 4H), 7.99 (d, *J* = 8.9 Hz, 4H), 7.32 (d, *J* = 9.1 Hz, 2H), 4.00 (s, 3H). **^{13}C NMR** (126 MHz, DMSO-*d*$_6$, r.t.): *δ* [ppm] = 168.0, 166.2, 163.7, 133.3, 132.7, 130.3, 129.1, 128.5, 124.3, 115.6, 113.6, 56.2. **MS** (ESI+) *m/z* (%) = 496.9 (100) [M - BF_4]$^+$.

25

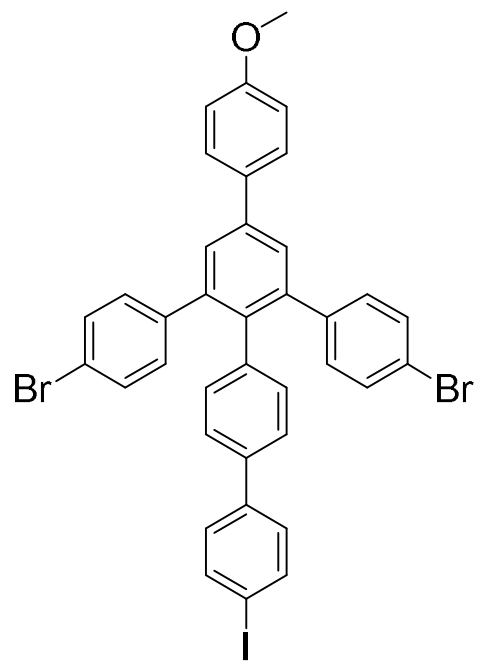

Benzoic anhydride (60.2 g, 266 mmol), **24** (11.1 g, 19.0 mmol) and **8** (6.84 g, 19.0 mmol) were purged with Ar and stirred at 150 °C for 4 h. After cooling to r.t., the reaction mixture was dissolved in DCM:CH (1:1) and was purified by column chromatography (CH:DCM = 1:1; R_f = 0.73). Recrystallization from DCM gave **25** (3.83 g, 4.96 mmol, 26 %) as a white solid.

Chemical formula: $C_{37}H_{25}Br_2IO$. **^{1}H NMR** (500 MHz, $CDCl_3$, r.t.): *δ* [ppm] = 7.71 (d, *J* = 8.5 Hz, 2H), 7.62 (d, *J* = 8.8 Hz, 2H), 7.59 (s, 2H), 7.31–7.27 (m, 8H), 7.01–6.98 (m, 6H), 6.88 (d, *J* = 8.4 Hz, 2H), 3.87 (s, 3H). **^{13}C NMR** (126 MHz, $CDCl_3$, r.t.): *δ* [ppm] = 159.6, 141.4, 140.7, 140.4, 140.0, 138.4, 137.8, 137.6, 136.8, 132.6, 132.3, 131.6, 131.1, 128.7, 128.2, 128.1, 126.1, 120.9, 114.4, 93.0, 55.5. **MS** (MALDI pos) *m/z* (%) = 771.9 (100) [M]$^+$.

26

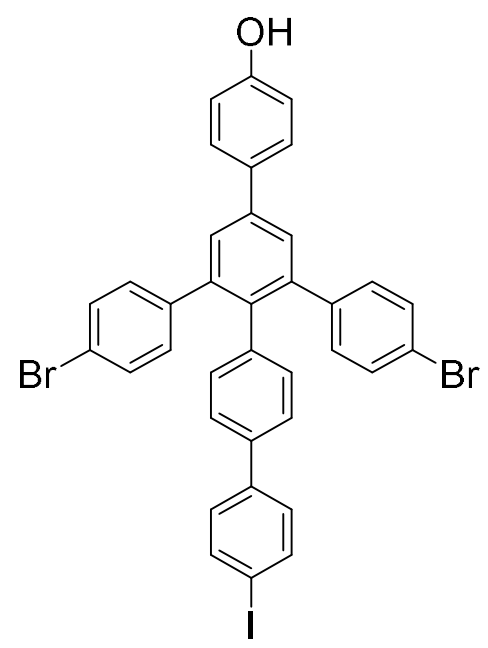

Under an Ar atmosphere, **25** (3.83 g, 4.96 mmol) wa stirred in DCM (150 mL) at -78°C. BBr_3 (10 mL, 10 mmol, 1 M in DCM) was slowly added and the mixture was stirred at r.t. for 18 h. The mixture was slowly diluted with DCM and water. The aqueous phase was extracted with DCM and the combined organic phase was washed with water, aq HCl (10 %) and brine, and dried over $MgSO_4$. The crude product was purified by column chromatography (DCM; R_f = 0.48) to give **26** (2.82 g, 7.72 mmol, 75 %) as a white solid.

Chemical formula: $C_{36}H_{23}Br_2IO$. **^{1}H NMR** (500 MHz, $CDCl_3$, r.t.): δ [ppm] = 7.71 (d, J = 8.5 Hz, 2H), 7.57 (s, 2H), 7.57 (d, = 8.6 Hz, 2H), 7.32–7.25 (m, 8H), 7.00 (d, Z = 8.5 Hz, 4H), 6.92 (d, J = 8.6 Hz, 2H), 6.88 (d, J = 8.4 Hz, 2H) **^{13}C NMR** (126 MHz, $CDCl_3$, r.t.): δ [ppm] = 155.7, 141.4, 140.8, 140.3, 140.0, 138.2, 137.8, 137.6, 136.7, 132.9, 132.3, 131.5, 131.1, 128.7, 128.6, 128.2, 126.1, 121.0, 116.0, 93.1. **MS** (EI+) m/z (%) = 757.8 (100) $[M]^+$.

27

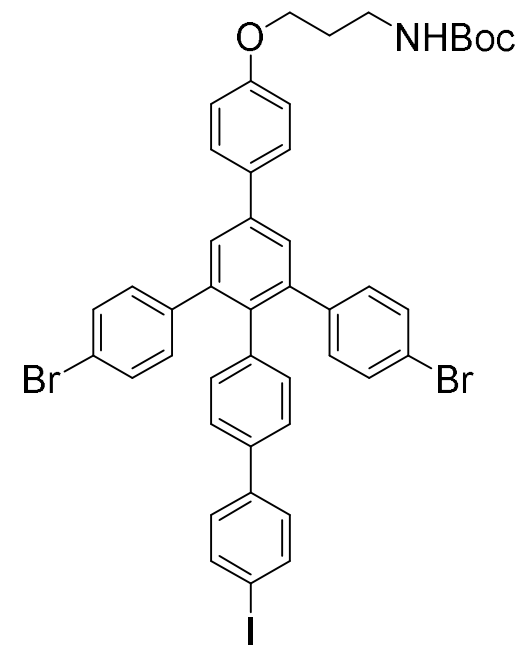

26 (500 mg, 0.658 mmol), Cs_2CO_3 (644 mg, 1.98 mmol), KI (3.3 mg, 19.8 μmol) and 3-(boc-amino)-propyl bromide (344 mg, 1.45 mmol) were stirred in acetone (70 mL) under reflux for 18 h. After the mixture was cooled to r.t., the reaction mixture was diluted with DCM and water. The aqueous phase was extracted with DCM and the combined organic phase was washed with water and brine, and dried over $MgSO_4$. The was purified by column chromatography (DCM ; R_f = 0.55). Recrystallization from MeOH gave **27** (575 mg, 0.627 mmol, 95 %) as a white solid.

Chemical formula: $C_{44}H_{38}Br_2INO_3$. **^{1}H NMR** (500 MHz, $CDCl_3$, r.t.): δ [ppm] = 7.71 (d, J = 8.5 Hz, 2H), 7.61 (d, J = 8.8 Hz, 2H), 7.58 (s, 2H), 7.30 (d, J = 8.5 Hz, 4H), 7.30–7.25 (m, 4H), 7.01–6.98 (m, 6H), 6.90 (d, J = 8.5 Hz, 2H), 4.77 (br, 1H), 4.08 (t, J = 5.8 Hz,2 H), 3.40–3.30 (m, 2H), 2.00 (t, J = 6.1 Hz, 2H), 1.44 (s, 9H). **^{13}C NMR** (126 MHz, $CDCl_3$, r.t.): δ [ppm] = 158.9, 156.2, 141.4, 140.8, 140.2, 140.0, 138.2, 137.8,

137.6, 136.8, 132.6, 132.1, 131.5, 131.1, 128.7, 128.2, 128.2, 126.0, 121.0, 115.1, 93.1, 38.2, 31.6, 29.6, 28.6. **MS** (MALDI pos) *m/z* (%) = 913.1 (38) $[M]^+$, 814.1 (91) $[M - Boc]^+$, 756.0 (100) $[M - C_3H_6NHBoc]^+$.

28

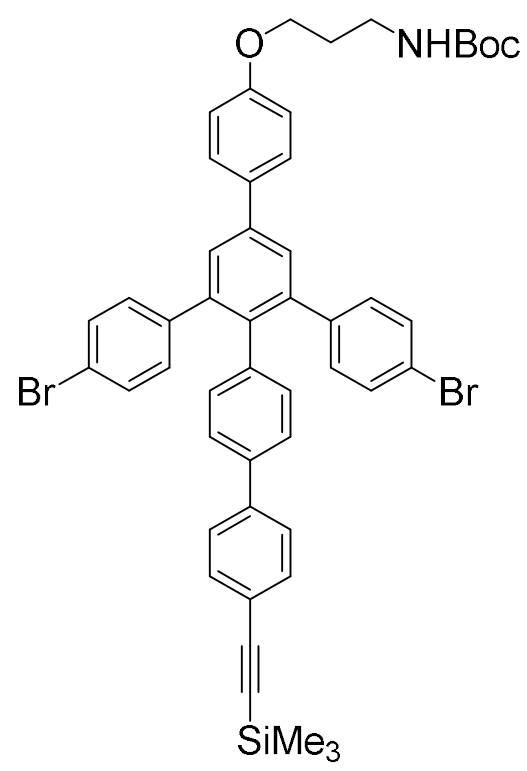

27 (200 mg, 0.22 mmol), $PdCl_2(PPh_3)_2$ (9.3 mg, 13.1 µmol), PPh_3 (10.3 mg, 39.3 µmol) and CuI (83.2 mg, 0.44 mmol) were dissolved in THF (10 mL) and piperidine (20 mL) in a dry Schlenk tube and purged with Ar for 1 h. TMS-acetylene (40.0 µL, 0.26 mmol) was added slowly to the solution, which was then stirred at r.t. for 18 h. The solution was diluted with DCM and water and the aqueous phase was extracted with DCM. After the combined organic phases were washed with water and brine, and dried over $MgSO_4$, the crude product was purified by column chromatography (DCM ; R_f = 0.50). **28** (183 mg, 0.21 mmol, 94 %) was obtained as white solid.

Chemical formula: $C_{49}H_{47}Br_2NO_3Si$. **^{1}H NMR** (500 MHz, $CDCl_3$, r.t.): δ [ppm] = 7.62–7.58 (m, 4H), 7.58–7.44 (m, 4H), 7.33–7.27 (m, 6H), 7.02–6.96 (m, 6H), 6.93–6.88 (m, 2H), 4.75 (br, 1H), 4.08 (t, *J* = 6.0 Hz, 2H), 3.38–3.32 (m, 2H), 2.00 (t, *J* = 6.1 Hz, 2H), 1.44 (s, 9H), 0.07 (s, 9H). **^{13}C NMR** (126 MHz, $CDCl_3$, r.t.): δ [ppm] = 158.9, 156.2, 141.4, 140.7, 138.5, 137.9, 136.7, 132.6, 132.5, 132.1, 132.2, 131.5, 131.1, 128.2, 128.1, 126.7, 126.7, 126.1, 120.9, 115.1, 95.0, 29.6, 28.5, 1.2. **MS** (MALDI pos) *m/z* (%) = 883.2 (100) $[M]^+$, 784.2 (40), 949.5 (25) $[M – (TMS\text{-}acetylene)]^+$, $[M - Boc]^+$, 726.2 (60) $[M - C_3H_6NHBoc]^+$.

29

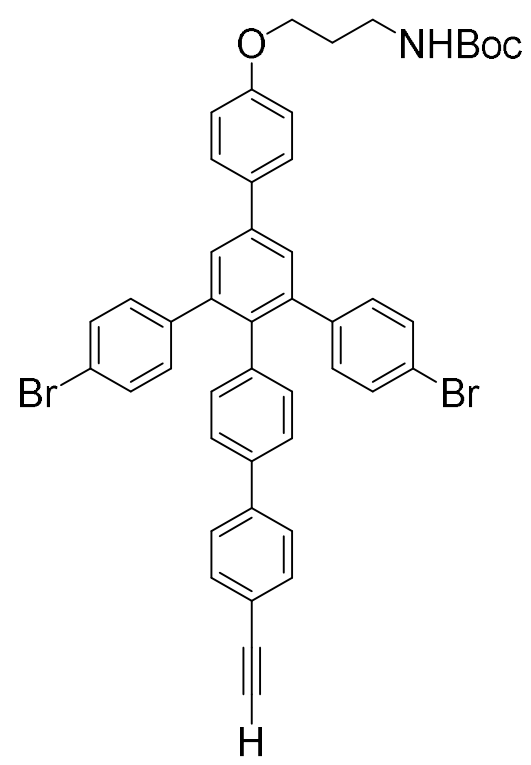

28 (183 mg, 0.21 mmol) and K_2CO_3 (228 mg, 1.64 mmol) were dissolved in THF (12 mL) and MeOH (6 mL) in a dry Schlenk tube and the reaction mixture was stirred under Ar at r.t. for 18 h. The reaction was diluted with water and DCM and the aqueous phase was extracted with DCM. After the combined organic phases were washed with water and dried over $MgSO_4$, the crude product was purified with column chromatography (DCM, R_f = 0.60). **29** (158 mg, 0.19 mmol, 94 %) was obtained as a white solid.

Chemical formula: $C_{46}H_9Br_2NO_3$. **^{1}H NMR** (500 MHz, $CDCl_3$, r.t.): δ [ppm] = 7.62–7.57 (m, 4H), 7.53–7.46 (m, 4), 7.33–7.29 (m, 6H), 7.03–6.97 (m, 6H), 6.92–6.86 (d, J = 8.4 Hz, 2H), 4.77 (br, 1H), 4.07 (t, J = 6.1 Hz, 2H), 3.38–3.31 (m, 2H), 3.11 (s, 1H), 2.06–1.96 (m, 2H), 1.44 (s, 9H). **^{13}C NMR** (126 MHz, $CDCl_3$, r.t.): δ [ppm] = 162.1, 158.9, 156.2, 141.4, 140.8, 140.3, 138.4, 137.9, 137.7, 132.4, 132.1, 131.5, 131.1, 128.2, 128.2, 126.7, 126.1, 121.1, 120.9, 115.1, 83.6, 77.8, 38.2, 31.2, 29.9, 28.6. **MS** (MALDI pos) m/z (%) = 811.1 (100) $[M]^+$, 712.1 (49) $[M - Boc]^+$, 654.1 (74) $[M - C_3H_6NHBoc]^+$.

31

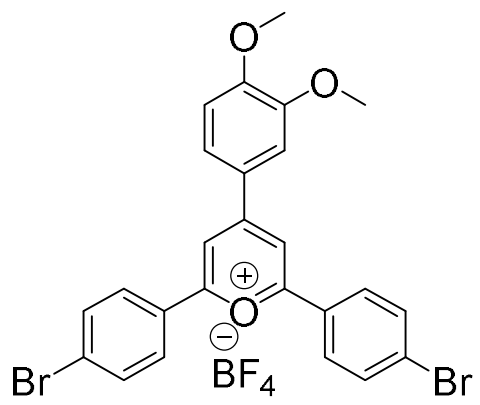

Under an Ar atmosphere, **30** (12.1 g, 73.4 mmol) and **16** (29.2 g, 146 mmol) were dissolved in 1,2-dichlorethane (25 mL) in a dry Schlenk tube. Boron trifluoride diethyl etherate (23.3 mL, 184 mmol) was added and the reaction mixture was stirred at 80 °C for 3 h. The solution was diluted with DCM and then precipitated in ethylacetate (200 mL). The precipitate was filtered off and washed with ethylacetate. **31** (5.08 g, 8.27 mmol, 11 %) was obtained as a red solid.

Chemical formula: $C_{25}H_{19}BBr_2F_4O_3$. **^{1}H NMR** (500 MHz, DMSO-d_6, r.t.): δ [ppm] = 9.03 (s, 2H), 8.55 (d, J = 8.7 Hz, 1H), 8.4 (d, J = 8.7 Hz, 1H), 8.5 (d, J = 8.9 Hz, 4H), 8.3 (dd, J = 8.7 Hz, J = 2.3 Hz, 1H), 8.01 (d, J = 8.9 Hz, 4 H), 4.02 (s, 3H), 4.00 (s, 3H). **^{13}C NMR** (126 MHz, DMSO-d_6, r.t.): δ [ppm] = 167.8, 163.8, 156.4, 149.8, 133.0, 132.8, 130.2, 129.0, 128.4, 126.6, 124.5, 113.7, 112.4, 56.5, 56.5. **MS** (ESI+) m/z (%) = 526.9 (100) $[M - BF_4]^+$.

32

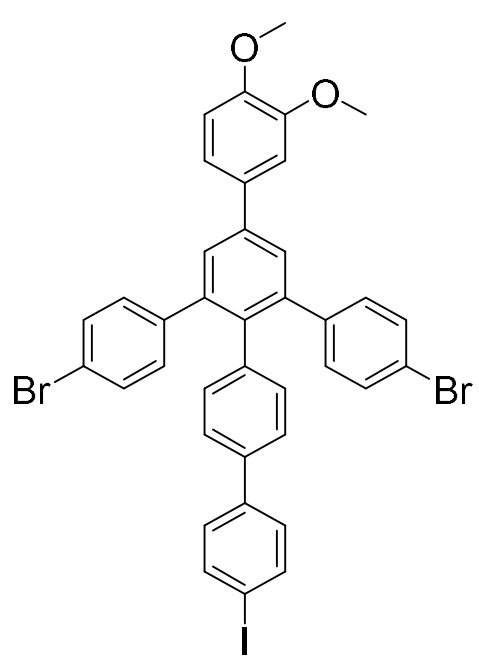

Benzoic anhydride (18.7 g, 82.7 mmol), **31** (5.08 g, 8.27 mmol) and **8** (2.98 g, 8.27 mmol) were purged with Ar and stirred at 150 °C for 4 h. After cooling to r.t,. the reaction mixture was dissolved in DCM:CH (1:1) and was purified by column chromatography (CH:DCM = 1:1; R_f = 0.32). Recrystallization from DCM gave **32** (0.881 g, 1.09 mmol, 13 %) as a white solid.

Chemical formula: $C_{38}H_{27}Br_2IO_2$. **^{1}H NMR** (500 MHz, $CDCl_3$, r.t.): δ [ppm] = 7.73 (d, J = 8.5 Hz, 2H), 7.60 (s, 2H), 7.31 (d, J = 8.4 Hz, 4H), 7.30–7.27 (m, 4H), 7.22 (dd, J = 8.4 Hz, J = 2.1 Hz, 1H), 7.17 (d, J = 2.1 Hz, 1H), 7.02 (J = 8.5 Hz, 4H), 6.96 (d, J = 8.4 Hz, 1H), 6.88 (d, J = 8.5 Hz, 2H), 3.94 (s, 3H), 3.93 (s, 3H). **^{13}C NMR** (126 MHz, $CDCl_3$, r.t.): δ [ppm] = 149.5, 149.1, 141.4, 140.8, 140.5, 140.0, 138.2, 137.8, 137.0, 133.1, 132.1, 131.5, 131.0, 128.7, 128.7, 126.1, 121.0, 119.7, 111.6, 110.5, 93.0, 56.2, 56.1. **MS** (MALDI POS) m/z (%) = 801.9 (100) $[M]^+$, 676.1 (20) $[M - I]^+$.

33

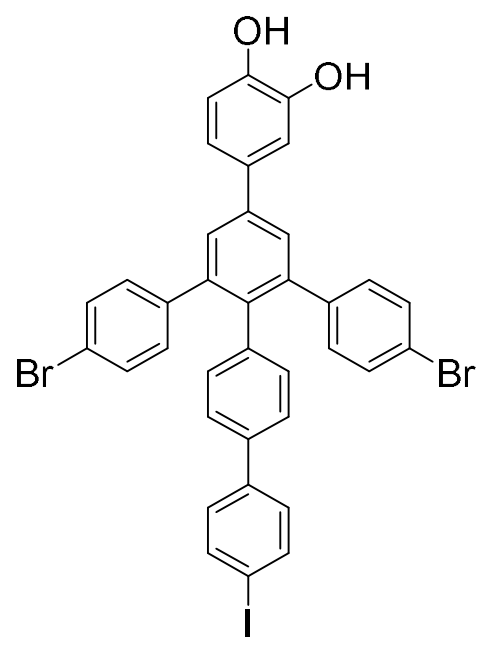

32 (823 mg, 1.03 mmol) in DCM (30 mL) is stirred under Ar at -78°C. BBr_3 (2 mL, 2 mmol, 1 M in DCM) was slowly added and the mixture was stirred at r.t. for 18 h. The mixture was slowly diluted with DCM and water. The aqueous phase was extracted with DCM and the combined organic phase was washed with water, aq HCl (10 %) and brine, and dried over $MgSO_4$. The crude product was purified by column chromatography (DCM; R_f = 0.12) to give **33** (565 mg, 0.79 mmol, 71 %) as a white solid.

Chemical formula: $C_{36}H_{23}Br_2IO_2$. **^{1}H NMR** (500 MHz, CD_2Cl_2, r.t.): δ [ppm] = 7.71 (d, J = 8.5 Hz, 2H), 7.57 (s, 2H), 7.31 (d, J = 8.6 Hz, 4H), 7.30–7.24 (m, 4H), 7.22 (d, J = 1.9 Hz, 1H), 7.13 (dd, J = 8.2 Hz, J = 1.9 Hz, 1H), 7.00 (d, J = 8.6 Hz, 4H), 6.96 (d, J = 8.2 Hz, 1H), 6.89 (d, J = 8.4 Hz, 2H). **^{13}C NMR** (126 MHz, CD_2Cl_2, r.t.): δ [ppm] = 144.1, 143.7, 141.5, 140.6, 140.2, 140.0, 138.2, 137.9, 137.6, 137.0, 133.6, 132.1, 131.5, 131.1, 128.7, 128.7, 126.1, 121.0, 120.0, 116.0, 114.4, 93.0. **MS** (MALDI pos) m/z (%) = 1023.1 (100) $[M+DCTB]^+$, 772.0 (84) $[M]^+$.

34

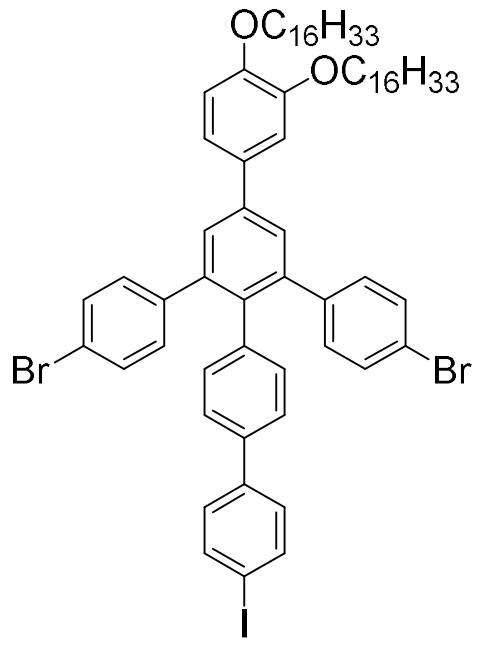

33 (200 mg, 0.257 mmol), Cs_2CO_3 (253 mg, 0.775 mmol), KI (1.29 mg, 7.75 μmol) and hexadecyl bromide (346 mg, 1.14 mmol) were stirred in acetone (30 mL) under reflux for 18 h. After the mixture was cooled to r.t., it was diluted with DCM and water. The aqueous phase was extracted with DCM and the combined organic phase was washed with water, aq HCl (10 %) and brine, and dried over $MgSO_4$. The solution was concentrated and a white solid precipitated after the addition of MeOH. The solid was collected and washed with MeOH, and then purified by column chromatography (CH/DCM (1:1); R_f = 0.88). **34** (314 mg, 0.257 mmol, 99 %) was obtained as a white solid.

Chemical formula: $C_{68}H_{87}Br_2IO_3$. **^{1}H NMR** (500 MHz, $CDCl_3$, r.t.): δ [ppm] = 7.74–7.71 (m, 2H), 7.60–7.54 (m, 2H), 7.34–7.27 (m, 8H), 7.22–7.15 (m, 2H), 7.02–6.95 (m, 4H), 6.94 (d, *J* = 1.0 Hz, 1H), 6.93–6.87 (m, 2H), 4.08–3.97 (m, 4H), 1.87–1.78 (m, 4H), 1.50–1.44 (m, 4H), 1.30–1.20 (m, 48H), 0.90–0.85 (m, 6H). **^{13}C NMR** (126 MHz, $CDCl_3$, r.t.): δ [ppm] = 149.7, 149.5, 141.5, 140.7, 140.6, 140.6, 140.0, 138.4, 137.8, 136.7, 133.0, 132.1, 131.7, 131.6, 131.1, 128.7, 128.3, 120.9, 119.9, 114.1, 113.2, 93.0, 69.5, 69.4, 52.4, 32.1, 29.9, 29.8, 29.7, 29.7, 29.5, 29.5, 29.5, 29.4, 29.4, 26.2, 26.2, 26.1, 22.9, 14.3. **MS** (MALDI pos) *m/z* (%) = 1222.4 (100) $[M]^+$.

35

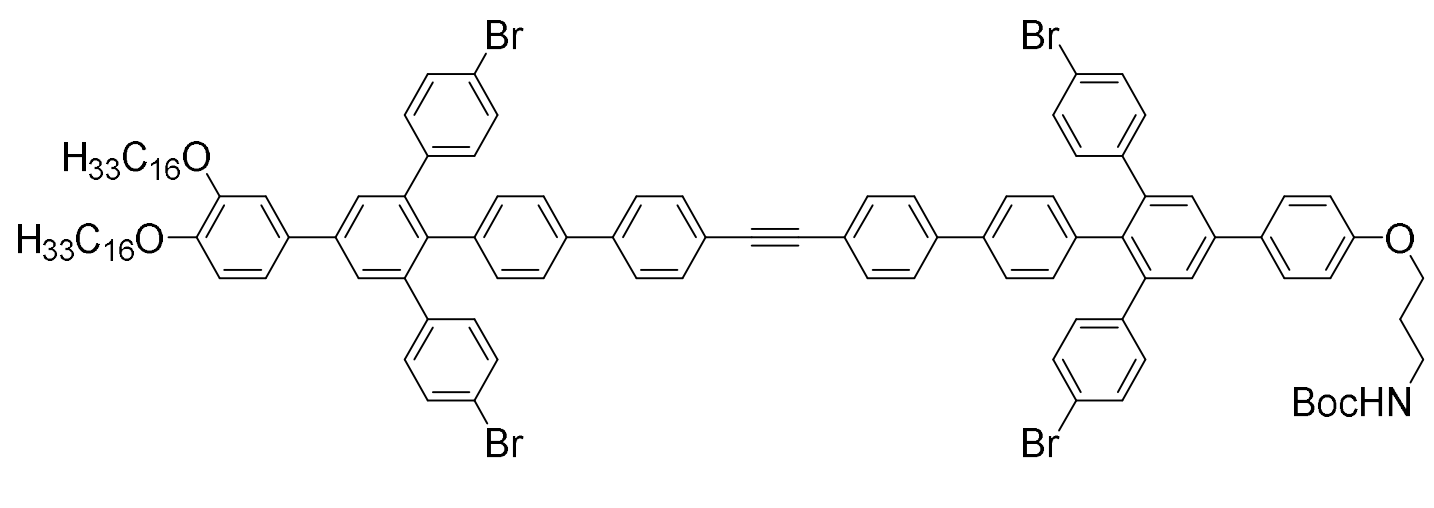

29 (100 mg, 81.9 μmol), $PdCl_2(PPh_3)_2$ (3.4 mg, 4.91 μmol), CuI (31.1 mg, 0.164 mmol) and PPh_3 (3.86 mg, 14.7 μmol) were dissolved in a dry Schlenk tube in piperidine (8 mL) and purged with Ar for 1 h. **34** (70.0 mg, 86.1 μmol) in dry and Ar saturated THF (4 mL) was added and the reaction mixture was stirred at r.t. for 18 h. The reaction was diluted with water and DCM. The aqueous layer was extracted with DCM and the combined organic phases were washed with water and brine, and dried over $MgSO_4$. The crude product was purified by column chromatography (DCM; R_f = 0.68) to give **35** (117 mg, 86.1 μmol, 75 %) as a white solid.

Chemical formula: $C_{114}H_{125}Br_4NO_5$. **^{1}H NMR** (500 MHz, $CDCl_3$, r.t.): δ [ppm] = 7.63–7.52 (m, 14H), 7.36–7.30 (m, 12H), 7.22–7.17 (m, 2H), 7.01–6.96 (m, 11H), 6.94–6.89 (m, 4H), 4.76 (br, 1H), 4.11–4.02 (m,

6H), 3.40–3.32 (m, 2H) 2.02 (t, J = 6.2 Hz, 2H), 1.89–1.78 (m, 4H), 1.52–1.47 (m, 4H), 1.46 (s, 9H), 1.34–1.24 (m, 48H), 0.89–0.86 (m, 6H). **^{13}C NMR** (126 MHz, $CDCl_3$, r.t.): δ [ppm] = 158.9, 156.2, 149.5, 149.4, 141.5, 141.4, 140.7, 140.7, 140.7, 140.3, 140.2, 138.3, 138.3, 137.9, 136.9, 136.9, 133.1, 132.6, 132.1, 132.0, 131.7, 131.7, 131.1, 128.2, 128.2, 128.1, 126.7, 126.2, 122.2, 120.9, 119.9, 115.1, 114.1, 113.2, 90.3, 90.3, 69.6, 69.4, 32.1, 29.9, 29.7, 29.7, 29.5, 29.5, 29.5, 29.4, 29.4, 28.6, 26.1, 22.9, 14.3. **MS** (MALDI pos) m/z (%) = 1908.7 (100) $[M]^+$.

36

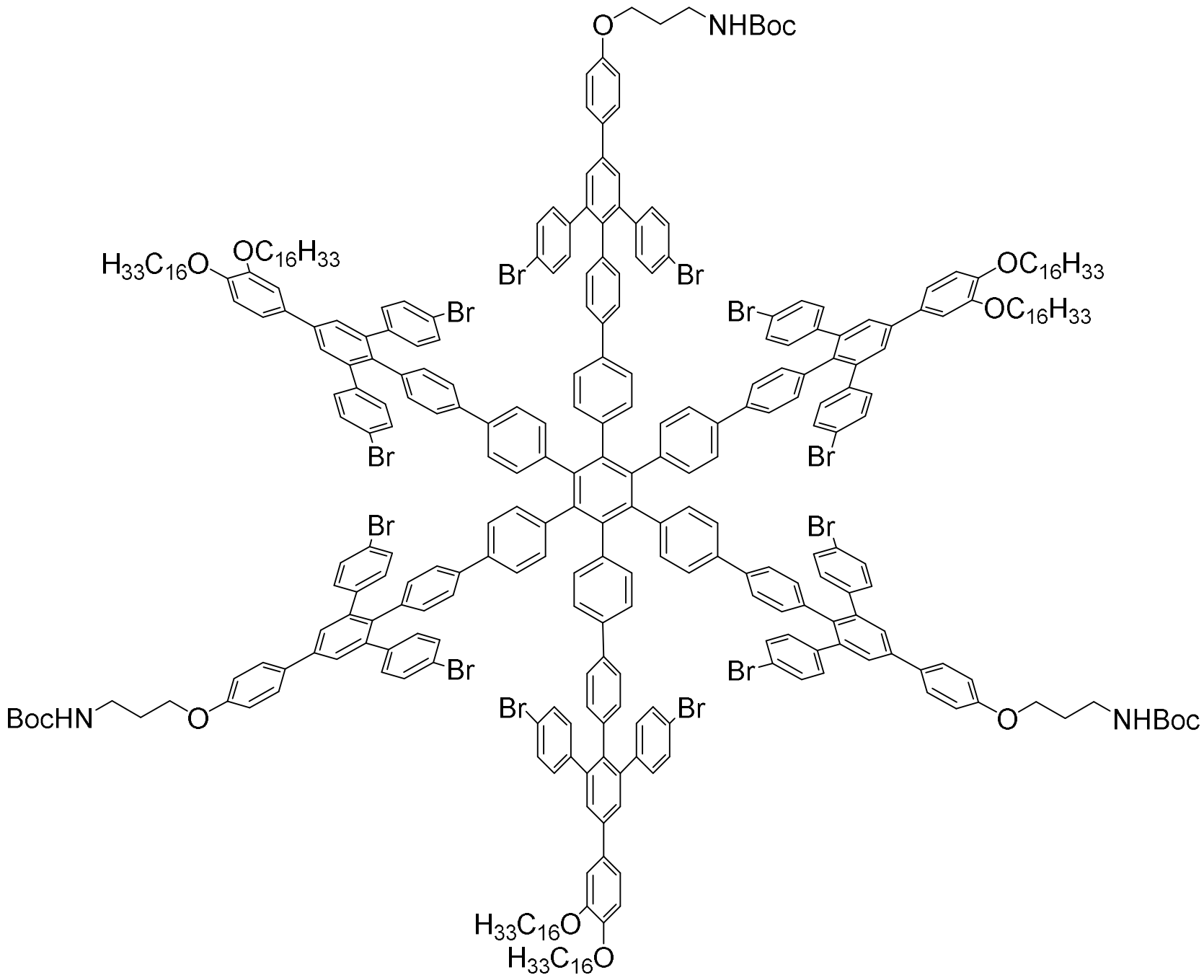

35 (117 mg, 61.2 µmol) was placed in a dry Schlenk tube and toluene (6 mL), which was purged with Ar, and $Co_2(CO)_8$ (4.20 mg, 12.3 µmol) were added. The mixture was stirred under reflux for 18 h. After cooling to r.t., the reaction was diluted in water and DCM. The aqueous layer was extracted with DCM and the combined organic phases were washed with water and brine, and dried over $MgSO_4$. The crude product was purified by recGPC and column chromatography (DCM, R_f = 0.56) and **36** (10.8 mg, 1.89 µmol, 10 %) was obtained as white solid along with the respective 1,2,4-isomer (DCM, R_f = 0.14, 19.5 mg, 3.41 µmol, 19 %).

Chemical formula: $C_{342}H_{375}Br_{12}N_3O_{15}$. **^{1}H NMR** (500 MHz, $CDCl_3$, r.t.): δ [ppm] = 7.63–7.53 (m, 15H), 7.32–7.27 (m, 18H), 7.25–7.04 (m, 36H), 7.04–6.82 (m, 48H), 6.82–6.68 (m, 12H), 4.75 (br, 3H), 4.10–4.01 (m, 18H), 3.38–3.31 (m, 6H), 2.05–1.96 (m, 6H), 1.89–1.76 (m, 12H), 1.53–1.46 (m, 12H), 1.45 (s, 27H), 1.39–1.33 (m, 12H), 1.27–1.22 (m, 132H), 0.89–0.86 (m, 18H). **^{13}C NMR** (126 MHz, $CDCl_3$, r.t.): δ [ppm] = 158.7, 156.2, 149.6, 149.4, 141.5, 141.4, 140.9, 140.4, 140.1, 139.7, 138.7, 137.3, 136.9, 133.1, 132.8, 132.0, 131.7, 131.0, 128.2, 128.1, 126.0, 125.5, 120.9, 119.7, 115.0, 114.2, 113.1, 69.7, 69.5, 32.1, 29.9, 29.7, 29.5, 29.5, 29.4, 28.7, 26.1, 22.8, 14.3. **MS** (MALDI pos) m/z (%) = 5759.9 (4) $[M+K]^+$, 5725.9 (100) $[M]^{+\bullet}$, 5647.0 (10) $[M-Br+H]^+$. **GPC** (THF, *vs* PS) M_p = $7.1\cdot10^3$ g/mol.

3

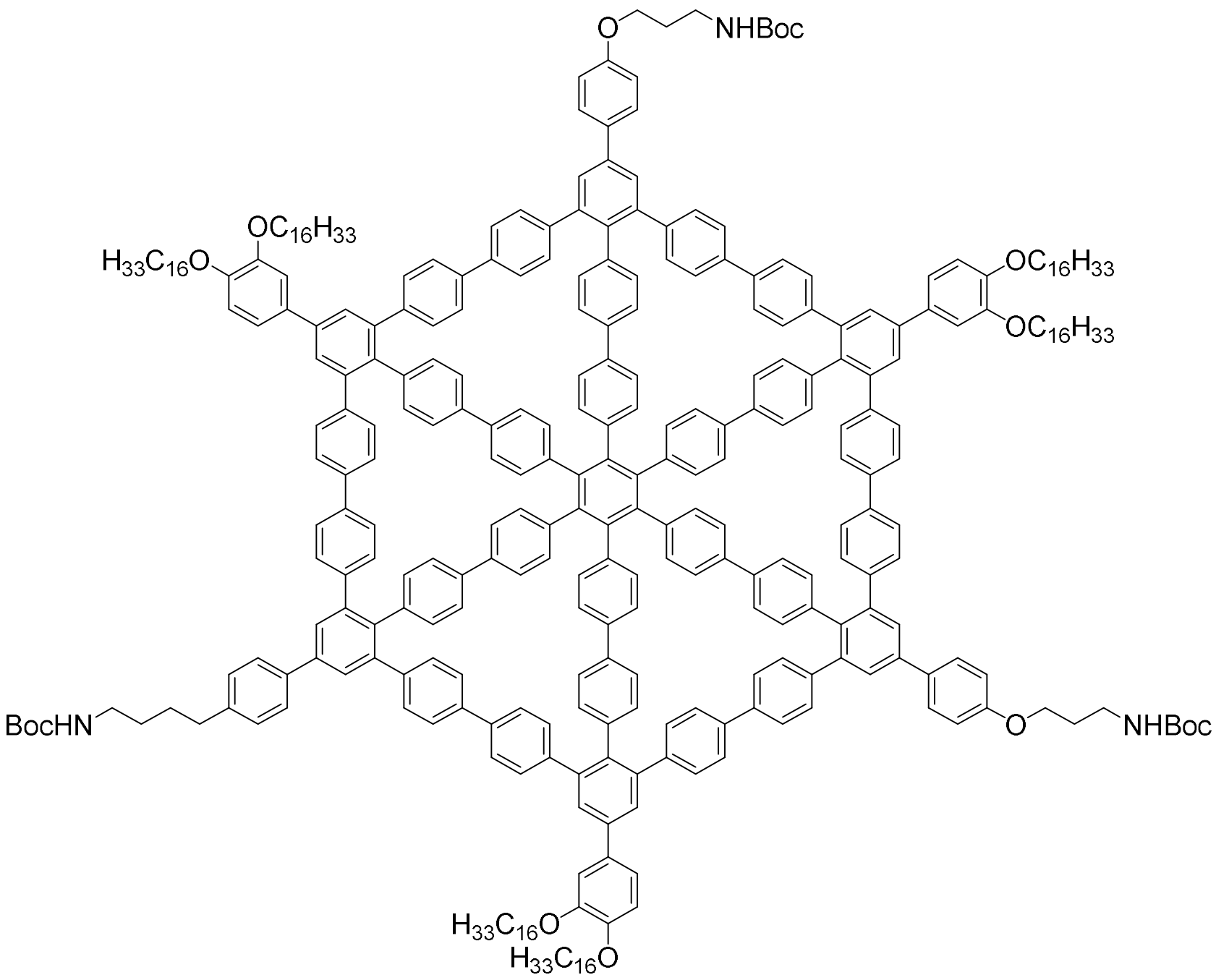

Under an Ar atmosphere, **36** (10.0 mg, 1.75 µmol), Ni(COD)$_2$ (10.0 mg, 36.4 µmol) and bipyridine (5.00 mg, 32.0 µmol) were dissolved in THF (8 mL) and COD (0.25 mL) in a microwave tube. The reaction mixture was stirred in a microwave at 120 °C for 12 min, and afterwards the solvent was removed under reduced pressure. The crude product was purified by column chromatography (DCM) and recGPC. Removal of the solvent gave **3** (5.34 mg, 1.12 µmol, 64 %) as creme-colored solid.

Chemical formula: $C_{342}H_{375}N_3O_{15}$. **^{1}H NMR** (700 MHz, CDCl$_3$, r.t.): δ [ppm] = 7.71–7.69 (m, 12H), 7.66–7.63 (m, 6H), 7.50–7.45 (m, 24H), 7.24–7.23 (m, 6H), 7.19–7.14 (m, 48H), 7.02–6.90 (m, 21H), 6.80–6.73 (m, 12H), 4.78 (br, 3H), 4.10–4.03 (m, 18H), 3.39–3.34 (m, 6H), 2.03–2.00 (m, 6H), 1.88–1.80 (m, 12H), 1.51–1.47 (m, 12H), 1.46 (s, 27H), 1.38–1.33 (m, 12H), 1.30–1.25 (m,132 H), 0.88 (q, J = 6.5 Hz, 18H). **^{13}C NMR** (176 MHz, CDCl$_3$, r.t.): δ [ppm] = 158.7, 156.2, 149.6, 149.2, 142.2, 142.2, 141.2, 140.1, 140.0, 139.7, 139.5, 138.3, 138.1, 137.3, 137.2, 136.4, 135.9, 133.6, 133.3, 131.7, 131.3, 130.5, 130.3, 128.4, 127.4, 125.4, 125.0, 124.3, 119.8, 115.0, 114.3, 113.1, 79.4, 69.6, 69.5, 66.1, 38.2, 32.1, 32.1, 29.9, 29.9, 29.8, 29.8, 29.8, 29.8, 29.8, 29.6, 29.5, 29.5, 29.5, 29.5, 28.6, 26.2, 26.2, 22.8, 22.8, 14.3.. **MS** (MALDI-pos) m/z (%) = 4784.9 (39) [M+NH$_4$]$^+$, 4767.9 (100) [M]$^+$. **GPC** (THF, *vs* PS) $M_p = 6.7\cdot10^3$ g/mol.

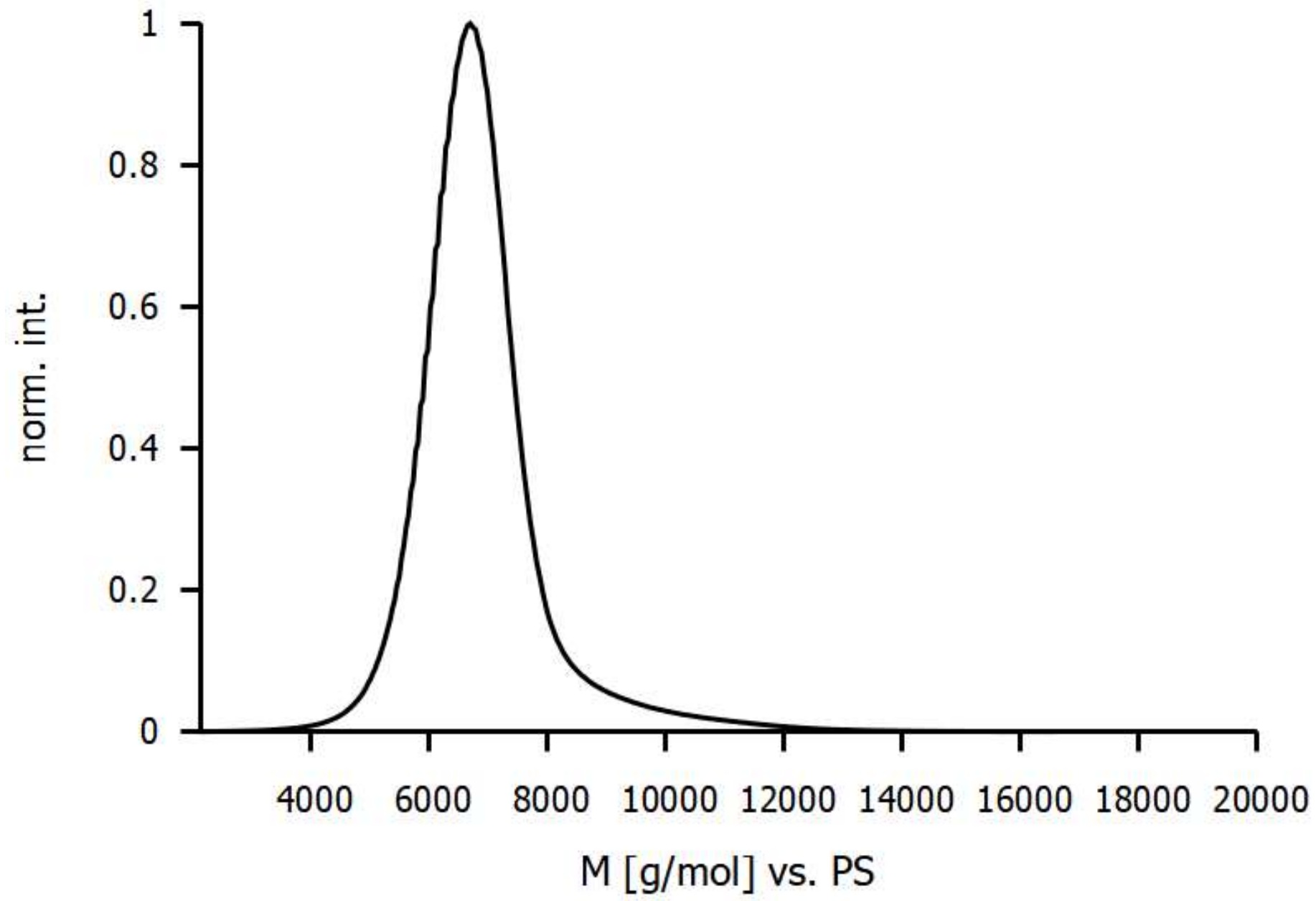

Figure S60 Molar mass distribution of the GPC analysis of **3** (M_p = 6824 g/mol).

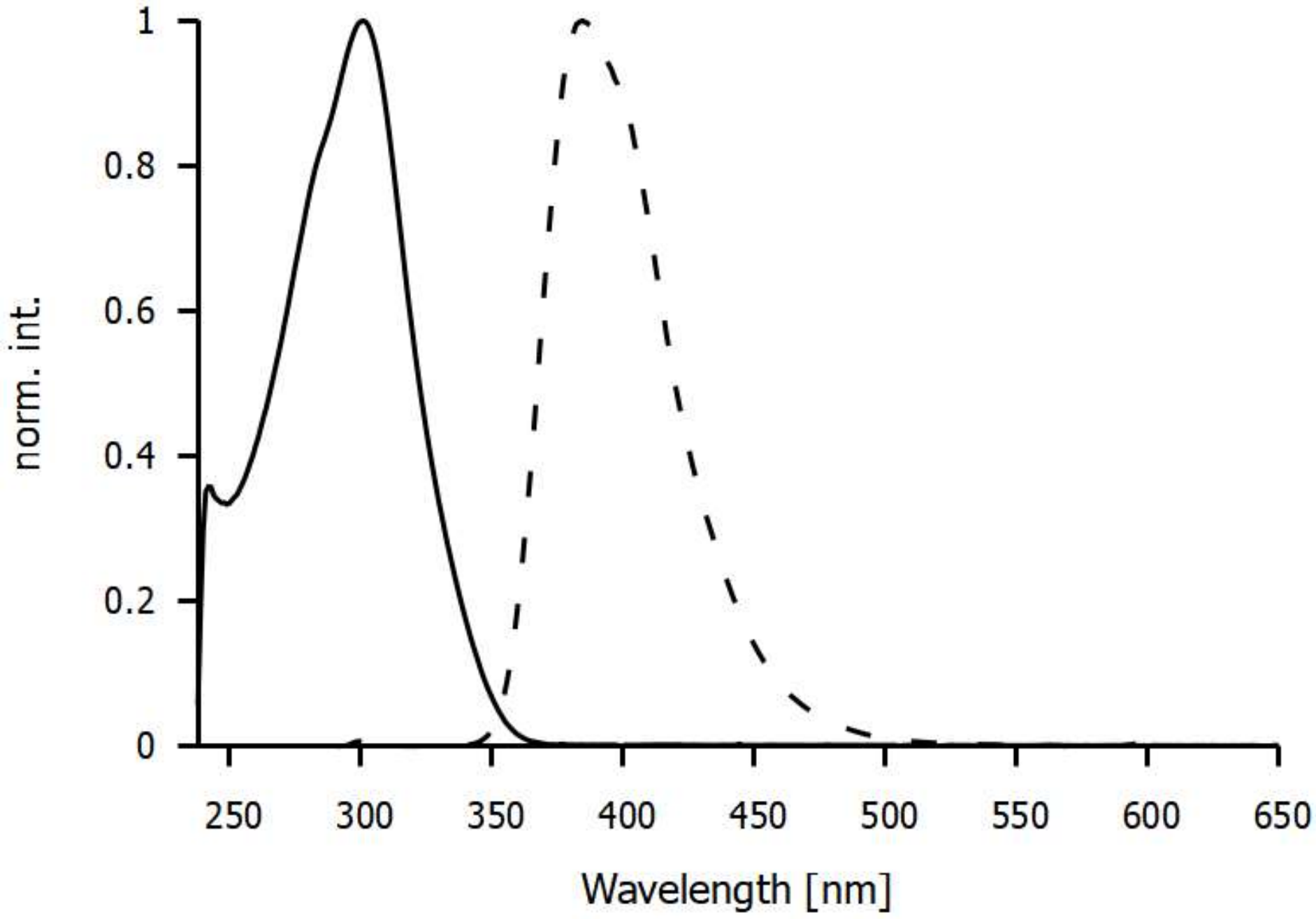

Figure S61 Normalized UV/vis spectra (solid) and fluorescence spectra (dashed) of **3** (maxima at UV/vis: 301 nm, F: 384.5 nm) in $CHCl_3$ at r.t. in air.
